# Existing evidence related to soil retention of phosphorus from on-site wastewater treatment systems in boreal and temperate climate zones: a systematic map

**DOI:** 10.1186/s13750-023-00300-7

**Published:** 2023-04-03

**Authors:** Ida Envall, Fritjof Fagerlund, Lena Johansson Westholm, Arvid Bring, Magnus Land, Charlotte Åberg, Neal R. Haddaway, Jon Petter Gustafsson

**Affiliations:** 1 The Swedish Research Council for Environment, Agricultural Sciences and Spatial Planning (Formas), Box 1206, 111 82 Stockholm, Sweden; 2 Department of Earth Sciences, Uppsala University, Villavägen 16, 752 36 Uppsala, Sweden; 3 School of Business, Society and Technology, Mälardalen University, P.O. Box 883, 721 23 Västerås, Sweden; 4 Leibniz-Centre for Agricultural Landscape Research (ZALF), Eberswalder Str. 84, 15374 Müncheberg, Germany; 5 Department of Soil and Environment, Swedish University of Agricultural Sciences, Box 7014, 750 07 Uppsala, Sweden

**Keywords:** OWS, OWTS, Septic systems, Septic tanks, Drainfields, Soil treatment, Adsorption, Precipitation, Phosphorus removal, Infiltration, Eutrophication, Sweden

## Abstract

**Background:**

In Sweden there are nearly one million soil-based on-site wastewater treatment systems (OWTSs). OWTSs may contribute to eutrophication of surface waters, due to the discharge of phosphorus (P). Hence, in certain cases, a high P removal rate (up to 90%) of OWTSs is required by Swedish authorities. Since these requirements may have costly consequences to property owners, it is debated whether they are too strict. In this debate, it is often claimed that the soil retention of P occurring in the natural environments may be underestimated by authorities. Soil retention is the inhibition of the transport of P through the ground, due to different chemical, physical and biological processes occurring there. These processes make the P transport slower, which may reduce the unwanted impact on receiving water bodies. However, the efficiency of soil retention of P remains unclear. The objective of this systematic map was to collect, code, organise and elucidate the relevant evidence related to the topic, to be able to guide stakeholders through the evidence base, and to support future research synthesising, commissioning, and funding. The systematic map was carried out in response to needs declared by the Swedish Agency for Marine and Water Management but the conclusions should be valid for a wider range of countries across boreo-temperate regions.

**Methods:**

Searches were made for peer-reviewed and grey literature using bibliographic databases, search engines, specialist websites, and stakeholder contacts. The references were screened for relevance according to a predefined set of eligibility criteria. A detailed database of the relevant studies was compiled. Data and metadata that enable evaluation and discussion of the character and quality of the evidence base were extracted and coded. Special focus was placed on assessing if existing evidence could contribute to policy and practice decision making. Descriptive information about the evidence base was presented in tables and figures. An interactive evidence atlas and a choropleth were created, displaying the locations of all studies.

**Review findings:**

234 articles out of 10,797 screened records fulfilled the eligibility criteria. These articles contain 256 studies, performed in the field or in the laboratory. Six different study types were identified, based on where the measurements were conducted. Most studies, including laboratory studies, lack replicates. Most field studies are observational case studies.

**Conclusions:**

It is not possible to derive valid generic measures of the efficiency of soil retention of P occurring in the natural soil environment from available research. Neither does the evidence base allow for answering the question of the magnitude of the potential impact of OWTSs on the P concentration in recipients on a general basis, or under what conditions OWTSs generally have such an impact. A compilation of groundwater studies may provide examples of how far the P may reach in x years, but the number of groundwater studies is insufficient to draw any general conclusions, given the complexity and variability of the systems. Future research should strive for replicated study designs, more elaborate reporting, and the establishment of a reporting standard.

**Supplementary Information:**

The online version contains supplementary material available at 10.1186/s13750-023-00300-7.

## Background

### On-site wastewater treatment systems

On-site wastewater treatment systems (OWTSs or OWSs) are facilities used for the disposal of wastewater from properties that are not connected to a public (municipal) wastewater treatment plant. OWTSs are common throughout the world, primarily in rural areas. In many countries, at least a quarter of the population is served by an OWTS [1].

Typically, an OWTS consists of a septic tank in which sludge and pathogens are removed, and a subsequent soil treatment area, where the wastewater is further purified from, e.g., phosphorus (P), nitrogen and pathogens.^1^ The total P concentration in household wastewater (toilet wastewater and greywater^2^ combined) entering a soil treatment system is usually quite high [2, 3], in Sweden often between 5 and 15 mg P L^−1^ [4], but part of this P may be retained by the soil material within the system and in the natural soil between the system and receiving surface water [5].

The release of P to surface waters is a global environmental concern, due to the risk of eutrophication. Source apportionment models show that municipal wastewater treatment plants and agricultural fields are the most important anthropogenic sources of P to European surface waters [6], but there are others as well. For example, OWTSs are suspected to contribute to the problem. It has been estimated that 13% of the total Swedish anthropogenic P discharge to the Baltic proper may be of such origin [7]. However, the estimates are inherently uncertain, partly due to the unclear extent of the retention of P caused by different processes occurring in the ground along the flow path between the facilities and adjacent surface water bodies [8].

In Sweden there are nearly one million OWTSs [9]. The authorisation and supervision of those are managed through the environmental and health authorities at the local municipalities. However, the Swedish Agency for Marine and Water Management (SwAM) is responsible for supervisory guidance, providing general recommendations related to OWTSs [4]. SwAM recommends that OWTSs, under certain conditions, shall remove up to 90% of the P entering the systems. Hence, municipal authorities may require this high level of P removal of specific facilities. To upgrade a facility or to connect a property to the municipal wastewater system, which sometimes is an alternative, may be costly to private individuals and households. Accordingly, there is an ongoing public debate in Sweden, as to whether the recommendations and requirements are too strict. In this debate, it is often claimed that the retention of P occurring in the natural soil environment between the facility and the receiving surface water may be significant, and possibly underestimated by authorities. For this reason, the Swedish Agency for Marine and Water Management has expressed a need for a scrutiny of all available evidence that might contribute to clarification of the matter.

This systematic map is aimed at surveying existing evidence related to retention of P in natural soil as wastewater released from OWTSs infiltrates the ground and migrates through the soil and groundwater system towards recipient surface waters. Soil retention in any artificial or amended materials within OWTSs is not considered. The objectives of the map are described in more detail in the section "Objective of the systematic map".

### The processes involved in soil retention

**Fig. 1:**
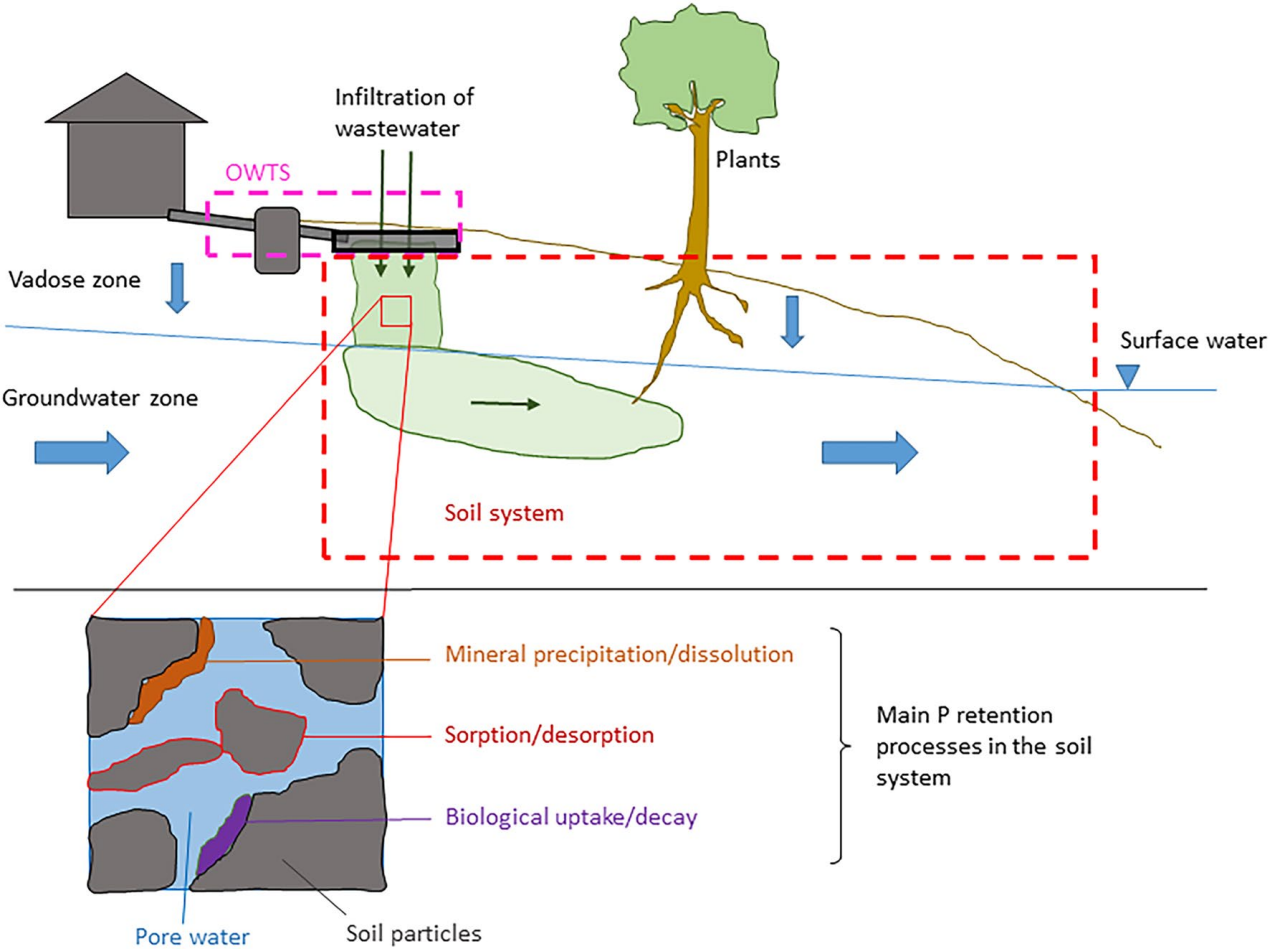
Conceptual model of the natural soil system where P retention is considered, and the main P retention processes in this system

A conceptual model of the system in focus, including the most important retention processes that take place there, is shown in Fig. 1. P in wastewater from an OWTS first migrates vertically through the vadose zone, i.e., the unsaturated soil zone that extends down to the groundwater table. Once it reaches the groundwater zone, P may migrate laterally with the groundwater until it reaches a recipient surface water. In this conceptual model there are, thus, two main zones—the vadose zone and the groundwater zone—where soil retention of P may occur. The main retention processes in these two zones are sorption to soil particle surfaces, precipitation of P to form solid mineral phases, and immobilisation of organic P. Sorption of P is expected to occur mostly to aluminium (Al) and iron (Fe) (hydr)oxides that may either be inherently present in the soil, or deposited over time as a result of transport of Al and Fe by the wastewater [10, 11]. Further, depending on the chemical conditions of the soil and wastewater, a range of Fe, Al and Ca phosphates may form in the soil [12, 13]. Finally, immobilisation of P into soil organic matter should not be overlooked as a possible additional retention mechanism. For example, in the 5–15 cm layer of three sand filters that had been loaded with septic tank effluent for about 20 years, organic P ranged from 35 to 43% of total P [11]. In addition, P may be assimilated by plants, for example grass, growing on the drainfield [14]. However, all these processes can be considered reversible through desorption, mineral dissolution and biological decomposition, respectively.

In the public debate, it is often claimed that P is “removed” by the retention mechanisms occurring in the soil. However, this is not entirely true, partly due to the reversibility of the P retention processes. Instead, these processes delay the transport of P through the soil, causing it to flow slower than the water. Nevertheless, the efficiency of soils to retain P is crucially important. Although most of the P will eventually reach surface waters, the more efficient the soil retention is, the less P will reach the surface water per unit of time, thus reducing its possible impact on surface water. The time scale of the transport and retention is hence important for the actual impact of P released from OWTSs.

### Stakeholder engagement

The systematic map was proposed by the Swedish Agency for Marine and Water Management, but a larger group of stakeholders was identified, and attempts were made to engage with them for the purpose of this evidence synthesis. Hence, the Swedish research council Formas arranged a stakeholder meeting to which representatives from the Swedish Agency for Marine and Water Management, municipalities, county administrative boards, research institutes, associations representing the interests of property owners, and consultancies within the wastewater treatment domain were invited to discuss, e.g., possible sources of evidence, study inclusion criteria and potential effect modifiers.

Initially the ambition was to conduct a systematic review. However, during the subsequent development of the protocol it became obvious that the complexity of the phenomenon and heterogeneity of the research field called for a systematic map rather than a systematic review, at least to begin with. At this stage, it was challenging to identify a stakeholder-relevant outcome that had been addressed in research to a satisfactory degree and in a way suitable for syntheses. In other words, although it could be concluded that a large number of studies dealt with the question of soil retention, most studies appeared not to have been designed in a way that enables the type of conclusions stakeholders primarily call for (i.e., quantitative estimates of the retention of P occurring along the passage between the facility and the recipient). Nevertheless, all those studies are relevant pieces of the puzzle, and should be considered and evaluated in the light of the overarching question. Systematic mapping was therefore selected as an appropriate evidence synthesis process for the purposes of cataloguing and collating a disparate and diverse body of evidence, to better understand the range, volume, and character of studies available, prior to any more in-depth full synthesis (e.g., using meta-analysis).

### Objective of the systematic map

The objective of the systematic map was to collect, code, organise and elucidate the relevant evidence related to soil retention of phosphorus from on-site wastewater treatment systems. The systematic map was carried out in response to needs declared by the Swedish Agency for Marine and Water Management; hence the conclusions should be valid for a Swedish context. Accordingly, the mapped evidence is confined to studies performed in soils that are not highly weathered, and (as regards field studies) in boreal and temperate climate zones.

The primary question for the systematic map was *What evidence exists related to soil retention of P from on-site wastewater treatment systems in boreal and temperate climate zones?*

Adopting a systematic mapping approach ruled out the possibility to *answer* the question of the effectiveness of soil retention of P from onsite wastewater treatment systems. Rather, the aim was to guide stakeholders through the evidence base, and to support future research synthesising, commissioning, and funding. To guide stakeholders, we discuss how and to what extent the evidence may contribute to policy and practice decision-making. To support future research synthesising, commissioning, and funding, we identify knowledge clusters where systematic reviews may be feasible, and knowledge gaps where more research may be needed.

### Definitions of the question components

In order to systematically map literature on a topic, it is necessary to precisely define eligibility criteria, i.e., criteria that a study need to fulfil to be included. This is done to ensure that all studies are assessed on the same grounds, and the aim is to avoid a biased collection of studies. The eligibility criteria are based on the components of the focal question. The components of the question of this systematic map are:

*Subject*: Phosphorus in primary or secondary treated domestic wastewater.

*Intervention*: Infiltration and flow of wastewater through natural (i.e., non-amended as regards P affinity) soils that are not highly weathered, in boreal and temperate climate zones.

*Comparator*: Depending on outcome. Examples are concentration of P in wastewater before infiltration, concentration of P in unaffected soil, or velocity of groundwater not affected by wastewater.

*Outcome*: Any outcome that can be used to evaluate the efficiency of the soil retention of P.

The eligibility criteria are described in more detail below.

## Methods

This systematic map was conducted according to a previously published protocol [15]. It follows the Collaboration for Environmental Evidence Guidelines [16] and uses the ROSES reporting standards [17] (see Additional file 1).

### Deviations from the protocol

During the screening process we realised that one area of research, focused on controlled application of wastewater to vegetated land surfaces, “irrigation systems”, may contain studies that fulfill our eligibility criteria. However, our original search strings may not have captured this type of study. Hence, we adapted the search strings to include this literature and conducted an updated search for articles. The modified search strings were designed to capture the irrigation system articles that were possibly missed by the first search, as well as any articles published after the original search. The search strings are presented in Additional file 2.

In the protocol it is stated that the screening at title/abstract level would be performed by one single reviewer, and that the reviewer would have three decision options during screening at this stage: include (1), exclude (2), or maybe (3). All titles/abstracts of the articles falling into category (3) would, according to the protocol, be screened by all authors, followed by a consensus decision. Instead all articles falling into this category (3) were screened in full text, by two reviewers (with blinded decisions).

According to the protocol we would include field studies as well as laboratory studies based on soil column experiments. However, another type of laboratory studies is also undoubtedly relevant, i.e., experimental studies performed in pilot-scale systems in laboratory environments. Accordingly, these studies were also included in the systematic map.

There is an area of research aimed at evaluating the P retention/removal efficiency in systems that are dependent on harvesting of vegetation that grows on the ground at the OWTS site. This is not the usual management practice. Obviously, P that has been assimilated by vegetation that is harvested and brought away is removed from the system. Consequently, measurements of P retention in such systems are not generalisable to systems that are managed in a conventional manner (i.e., without harvesting). These systems are to be regarded as “enhanced”. Hence, such studies have been excluded. This is not a deviation from the protocol but should be clarified since it was expressed somehow vaguely in the protocol.

Studies on soils developed in volcanic ash have been excluded, although this was not stated in the protocol. This type of soil is of no relevance to Swedish conditions.

The number of coded parameters has been reduced. Initially our expectation was that it would be possible to take one more step and perform a systematic review based on an appropriate fraction of research identified in this systematic map. In that case, it could have been a wise strategy to extract data that would be useful while synthesising results, already in the systematic mapping stage. However, during the work with the systematic map it appeared obvious that there is no fraction of research evidently suitable for a systematic review. Hence, we decided to reduce the parameter list to those most relevant at the mapping stage. Accordingly, we have focused on the parameters that are needed to enable evaluation and discussion of the character of the evidence base per se.

### Search for articles

An extensive search for academic articles and grey literature was conducted in bibliographic databases, search engines, websites of relevant organisations and through stakeholder contacts. A list of benchmark studies (see Additional file 3) was used during the development of the search strings and to test the comprehensiveness of the search.

#### Bibliographic databases

An initial search was performed in December 2019 in eight bibliographic databases listed in Table 1.

**Table 1:** Bibliographic databases used to search for articles

Database/platform	Search field	Language of search terms	Subscription information
Scopus	Title, Abstract, Keywords	English	Swedish Research Council Formas subscription
Web of Science Core Collection	Topic (search the fields: title, abstract and keywords)	English	Swedish Research Council Formas subscription includes: Science Citation Index Expanded; Social Sciences Citation Index; Arts & Humanities Citation Index; Conference Proceedings Citation Index- Science; Conference Proceedings Citation Index- Social Science & Humanities; Emerging Sources Citation Index
Academic Search Premier	Title, Abstract, Subject Terms, Author-Supplied Keywords	English	Swedish Research Council Formas subscription on Ebsco platform
CAB Abstracts	Title, Abstract, Heading Words	English	Swedish Research Council Formas subscription on Ovid platform
Directory of Open Access Journals	All fields	English	Free, does not require a subscription
DiVA	All fields	English and Swedish	Free, does not require a subscription
ProQuest Natural Science Collection	Title, Abstract, All subjects & indexing	English	Swedish Research Council Formas subscription includes: AGRICOLA; Agricultural Science database; Aquatic Sciences and Fisheries Abstracts; Biological Science database; Biological Science index; Earth, atmosphere & Aquatic Science database; Environmental Science database; Environmental Science index; Meteorological & Geoastrophysical Abstracts
SwePub	All fields	English and Swedish	Free, does not require a subscription

An updated search was performed at the end of September 2021 in four of the bibliographic databases (Scopus, Web of Science Core Collection, CAB Abstracts and ProQuest Natural Science Collection).

As stated in the systematic map protocol [15] we developed two different search strings, one for field studies and one for laboratory soil-column studies. All information about the searches is provided in Additional file 2. This file includes database and platform information, how the search strings were adapted to the search capabilities and syntax of each specific database/platform, limits to the searches, date of search, and the number of hits from each search. Searches were performed using mainly English search terms, except for in DiVA and SwePub, where also Swedish search terms were used. Since non-English articles often have a title and abstract in English, the use of English search terms captured articles written in several other languages. The searches were limited to include articles in English, Swedish, Norwegian, and Danish. The searches were not limited by publication date or document type.

#### Search engine

A search was performed in the academic search engine Google Scholar on December 13, 2019. It is not possible to use long search strings in Google Scholar, so we used nine simple search strings created to find both field studies and laboratory soil-column studies. Four of the search strings were in English and five in Swedish (see Additional file 2). The search results were ranked by relevance and the first 200 records for every search were exported from Google Scholar using Publish or Perish version 6 software [18].

#### Websites of relevant organisations

In order to find grey literature, we searched the websites of 51 organisations, for example government agencies, environmental protection agencies, environment research institutes, Swedish county administration boards, and (not peer-reviewed) journals. The organisations selected were primarily suggested by stakeholders and experts within the field. The search capabilities differed between the websites. In certain cases, Boolean operators could be used, whereas other websites did not even have a search box, making browsing through the website necessary to find publications. We used search terms in Swedish or English, and sometimes both, depending on the language of the website. All search terms used, and the number of matching results, are provided in Additional file 2.

#### Supplementary searches

In addition to the participants in the initial stakeholder meeting, we contacted four experts in the field (suggested by participants in the stakeholder meeting) to request studies and reports. We also looked through reference lists of included articles.

#### Assembling and managing search results

The results of all searches were collated using the reference management software EndNote. Duplicates were removed using the de-duplication method described by Bramer et al. [19]. In order to find and remove duplicates between the initial search in December 2019 and the updated search in September 2021, we used the de-duplication method described by Bramer and Bain [20].

### Article screening and study eligibility criteria

#### Screening process

To ensure consistency of eligibility decisions at title/abstract level, several subsets of records, in total 738, were screened by multiple reviewers with blinded decisions: 438 found in preliminary searches in bibliographic databases (8.1% of total found), and 300 found in the initial search in bibliographic databases (3.6% of total found). Disagreements were analysed and discussed until it was ascertained that the reviewers interpreted the agreed criteria equally and applied them in a consistent way. This made us confident that the remaining records could be single screened at the title/abstract level. The reviewer had three decision options for each record during screening at this stage: (1) include, (2) exclude, or (3) maybe. All records falling into category (1) or (3) were screened for relevance in full-text (see above, Deviations from the protocol). The screening process at full-text level was performed in pairs with blinded decisions. Any disagreement was solved by the screening-pair in the first instance. If disagreement persisted, the article was discussed by all reviewers to reach a consensus decision.

Reviewers were not allowed to assess the relevance of articles that they had authored themselves.

#### Eligibility criteria

All retrieved records were assessed for relevance using the following criteria:

*Eligible subject:* Phosphorus within primary or secondary treated domestic wastewater. The evidence should be valid for soil-based, on-site wastewater treatment systems designed to serve up to 200 person equivalents (in accordance with the definition made by the Swedish Agency for Marine and Water Management). As regards field studies, the wastewater must hence originate from single or groups of households and have been released to soil-based wastewater treatment systems. The upper limit is set to systems used by/constructed for 400 persons. Field studies on wastewater from municipal wastewater treatment plants are not eligible. However, as regards laboratory studies, using wastewater from municipal wastewater treatment plants is accepted. Studies focusing on, e.g., stormwater, industrial wastewater, wastewater from animal farms, or agricultural wastewater are not eligible, nor are studies using synthetic wastewater. Further, studies focusing on P leaching from sewage sludge are not eligible.

*Eligible intervention:* Infiltration of wastewater in natural (i.e., non-amended as regards P affinity) soils. The soil must not be highly weathered, and not developed in volcanic ash.^3^ Field studies must be performed within climate zones C or D according to the Köppen-Geiger climate classification system [21]. Field studies of P retention in wetlands or ditches are not eligible. However, laboratory column studies, evaluating natural soil materials from wetlands, are eligible. Studies focusing solely on plant uptake of phosphorus, or in which the plants are harvested and brought away, are not eligible.

*Eligible comparator:* The studies must include a comparator. The comparator depends on outcome but could be concentration of P in wastewater before infiltration, concentration of P in unaffected soil or velocity of groundwater not affected by wastewater.

*Eligible outcomes:* Any outcome that somehow allows (the efficiency of) retention of P in the soil to be evaluated, including—but not restricted to—measures of P retardation potential, P concentration in wastewater after infiltration, and concentration of P in groundwater or surface water affected by wastewater from OWTSs. Regarding the last example, concentration of P in surface water, the contribution of P from OWTSs, specifically, must be evaluated and analysed in the study. If not, the study is not eligible since no causality may be determined in those cases.

*Eligible types of study design:* Any controlled observational or experimental study design based on primary data. Modelling studies are eligible only if there are relevant primary data presented, used to validate the model. Laboratory studies must be based on soil column experiments or pilot-scale experiments. Studies based on laboratory batch experiments (“batch studies”) are not eligible.

*Eligible languages:* English, Swedish, Norwegian, and Danish.

A list of articles excluded at full text level is provided (Additional file 4). Reasons for exclusions are given for all articles excluded at this level.

### Study validity assessment

We have not performed validity assessments of included studies since we have not made any compilations or syntheses of the study results. Moreover, it would have been beyond the scope of this project to determine validity assessment criteria relevant for all different eligible study types a priori. Nevertheless, in the section "Mapping the quality of studies relevant to the question", we present some characteristic qualities of specific identified categories of studies: we describe and discuss general and recurrent merits and disadvantages of the respective study types when it comes to their ability to reliably answer (1) the specific research question of the respective study, and (2) our overarching question of soil retention of P occurring in the natural soil environment between the on-site wastewater facility and the recipient. Our ambition is to give a preliminary idea of the overall rigour of the evidence base, primarily relative the focal question.

### Data coding strategy

The metadata/data that have been coded/extracted from each study are listed in Table 2. Not every kind of data is coded/extracted for each study type that was identified. Abbreviations indicate the study types for which the respective data types have been coded/extracted: LC (laboratory column studies), LP (laboratory pilot-scale studies), VW (studies performed in the vadose zone, within facility), VN (studies performed in the vadose zone, in natural soil environment), G (studies performed in the groundwater zone), S (surface water studies).

**Table 2:** Kind of data/metadata that have been coded/extracted for what study types

Kind of data/metadata coded/extracted	Study type
Bibliographic information (name and e-mail of first author, title, publisher, year of publication)	LC, LP, VW, VN, G, S
Report type	LC, LP, VW, VN, G, S
Language	LC, LP, VW, VN, G, S
Country, state	LC, LP, VW, VN, G, S
Region	VW, VN, G, S
Site/case (if more than one)	LC, LP, VW, VN, G, S
Geographic coordinates	VW, VN, G, S
Climate zone	VW, VN, G, S
Study setting (field study or laboratory study)	LC, LP, VW, VN, G, S
Study domain (where in the system measurements were conducted)	VW, VN, G, S
Study set-up (soil column or laboratory pilot-scale system)	LC, LP
Aim of study	LC, LP, VW, VN, G, S
Reported measurement(s)/outcome(s)	LC, LP, VW, VN, G, S
Where in the report the relevant results/outcomes are reported	LC, LP, VW, VN, G, S
P species	LC, LP, VW, VN, G, S
General soil description	LC, LP, VW, VN, G, S
Soil order	LC, LP, VW, VN, G, S
Soil texture	LC, LP, VW, VN, G, S
Sand fraction	LC, LP, VW, VN, G, S
Silt fraction	LC, LP, VW, VN, G, S
Clay fraction	LC, LP, VW, VN, G, S
Diameter and soil package height of columns, or length, width and height of soil container	LC, LP
Whether the soil is virgin (that is, not used for wastewater infiltration previously) or not	LC, LP
Whether pH in soil or groundwater not affected by wastewater is reported or not	LC, LP, VW, VN, G
Whether pH in wastewater before infiltration is reported or not	LC, LP, VW, VN
Whether pH in wastewater after infiltration is reported or not	LC, LP, VW, VN
Whether pH in groundwater affected by wastewater discharge is reported or not	G
Whether concentration of oxalate-extractable iron in soil is reported or not	LC, LP, VW, VN, G, S
Whether concentration of dithionite-extractable iron in soil is reported or not	LC, LP, VW, VN, G, S
Whether concentration of oxalate-extractable aluminium in soil is reported or not	LC, LP, VW, VN, G, S
Whether concentration of dithionite-extractable aluminium in soil is reported or not	LC, LP, VW, VN, G, S
Type (origin) of wastewater	LC, LP
Duration/volume of wastewater load	LC, LP, VW, VN, G, S
Wastewater loading rate (volume/area/time unit) or (if data on wastewater loading rate is lacking) wastewater load (volume/time unit)	LC, LP, VW, VN, G
Whether wastewater loading rate is reported or inferable or not	LC, LP, VW, VN, G
Number and kind of entities giving rise to the P load (for example households, persons)	VW, VN, G
Type of OWTS (as described in the report—the terminology is not consistent)	VW, VN, G
Strategy to correct for dilution and/or background P leakage	G
Strategy to correct for dilution	VW, VN
Method to evaluate P retention	LC, LP, VW, VN, G
Whether available data allow calculation of P load to filter (g/m^3^ material) or not	LC, LP, VW
Whether the study is experimental or observational	VW, VN, G
Number of replicates (for experimental studies)	LC, LP, VW, VN, G
Method to evaluate P retention/possible impact from OWTSs	G
Method to infer possible impact from OWTSs	S
Whether it is possible to isolate the effect of OWSTs or not	S
Whether there is a comparison with control without OWTSs or not	S
Whether there are poor systems (i.e., without soil infiltration) in the catchment area or not	S

The data sheet was discussed, evaluated, and updated several times before being applied. To ensure a repeatable and consistent data coding, data from a subset of articles were then coded in blind by two reviewers and all disagreements were discussed to ensure consistent interpretation of the instructions. Five of the reviewers were involved in this procedure, each of them coding data from at least 15 articles. Thereafter, one reviewer performed most of the data coding, but about 15% of the extracted data were double-checked by another reviewer.

A systematic map database, i.e., a searchable spreadsheet containing the meta-data and data presented above, was created. If multiple studies were reported within one article, each study was coded separately in the database. The database is available in Additional file 5.

### Data mapping method

**Fig. 2:**
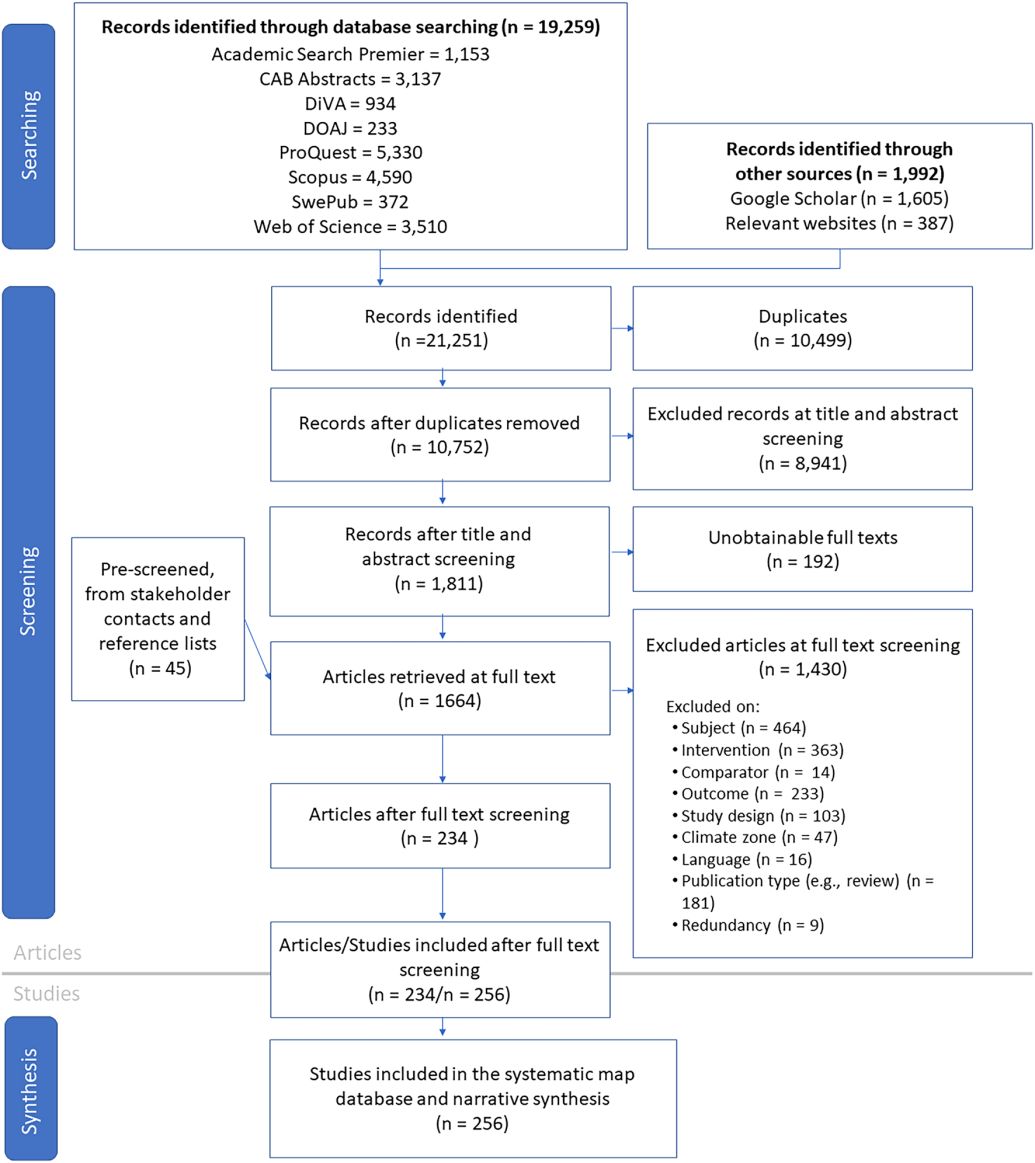
Flow diagram showing the screening process [23]

The numbers of articles found and retained at each stage of the review process are presented in a flow diagram in the section "Review descriptive statistics" (Fig. 2). Descriptive information about the evidence base, such as publication year, publication type, study country and climate zone are presented in tables and figures in the section "Mapping the quantity of studies relevant to the question". Different study types have been defined, and the categorisation is visualised through a flow chart.

We have produced an evidence atlas, using the tool EviAtlas [22]. The evidence atlas is an interactive cartographic map that plots the location of all field studies in geographical space, allowing the user to interrogate and filter studies and see their summary information. The interactive, html-based version is provided as an additional file alongside a static screenshot in Fig. 7. Moreover, a choropleth was created, showing the distribution of all studies over the world, including laboratory studies.

For the sake of clarity, some descriptive data are also presented for each respective study type under the subheading General overview of the study type in the section "Mapping the quality of studies relevant to the question". Examples are age of studied facilities (duration of wastewater flow), size of studied facilities, filter materials used, methods to evaluate P retention, study design (experimental or observational) and number of replicates.

## Review findings

### Review descriptive statistics

The initial searches in all bibliographic databases, Google Scholar and relevant websites resulted in a total number of 8248 unique records, after removal of duplicates. The updated search in September 2021 added 2545 new records after removal of duplicates, resulting in a total number of 10,793 records. During the screening process 41 more duplicates were identified, giving a total number of 10,752 unique records (and totally 10,499 removed duplicates). Moreover, 45 more records were provided by stakeholder contacts or identified by looking through bibliographies of relevant reviews and reports found during the article screening.

The literature screening stages are presented as a flow diagram in Fig. 2.

All eligible articles are found in Additional file 5 and a list of studies excluded at full text level, together with reasons for exclusion, is found in Additional file 4. A list of unobtainable articles is found in Additional file 6.

### Mapping the quantity of studies relevant to the question

#### Publication types

**Fig. 3:**
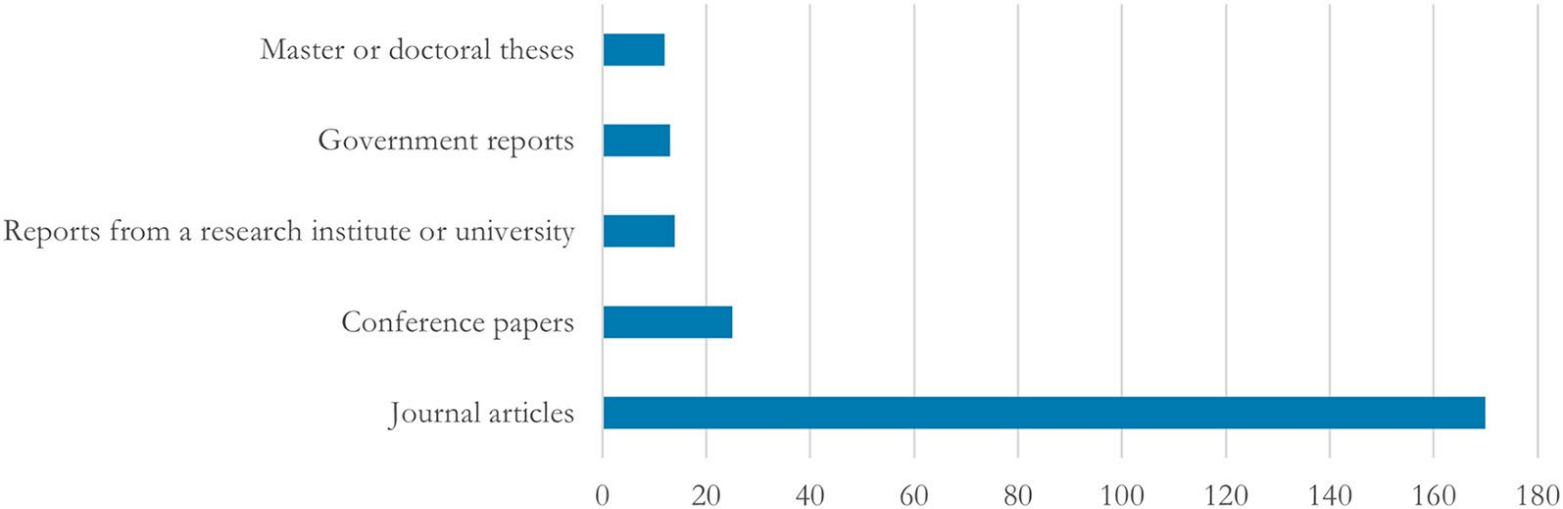
Distribution of types of publications

In total, 234 articles met the inclusion criteria (Fig. 2). A majority of the included articles, i.e., 170, are journal articles; 25 are conference papers; 14 are reports from a research institute or university; 13 are government reports; 12 are master or doctoral theses^4^ (Fig. 3). That is, a considerable part of the included literature is grey.

#### Publication years

**Fig. 4:**
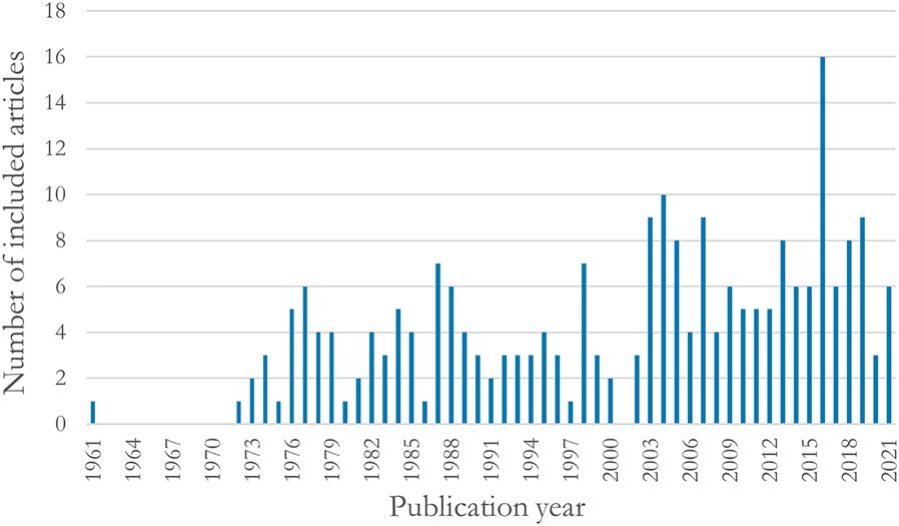
Numbers of included articles published each respective year during the period 1961 to 2021

In Fig. 4 the number of included articles published per year is shown. The oldest included article is from 1961, the most recent one from 2021.

#### Countries

**Fig. 5:**
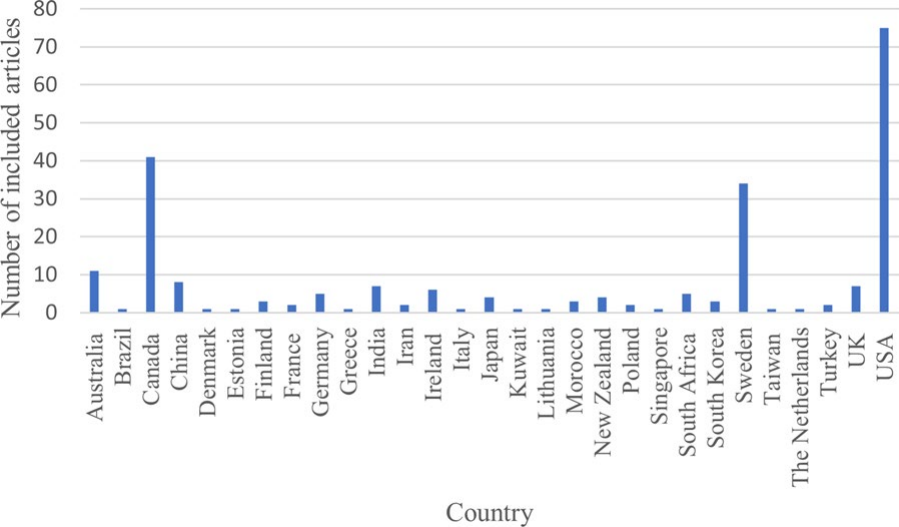
Number of articles from each represented country

Twenty-nine countries are represented in the systematic map (Fig. 5). Most articles (n = 75) are from the USA, followed by Canada (n = 41) and Sweden (n = 34). Most of the articles from the USA are from the state of North Carolina (n = 10), followed by Florida (n = 9). Most of the Canadian studies are from the province of Ontario (n = 32) followed by British Columbia (n = 4).

**Fig. 6:**
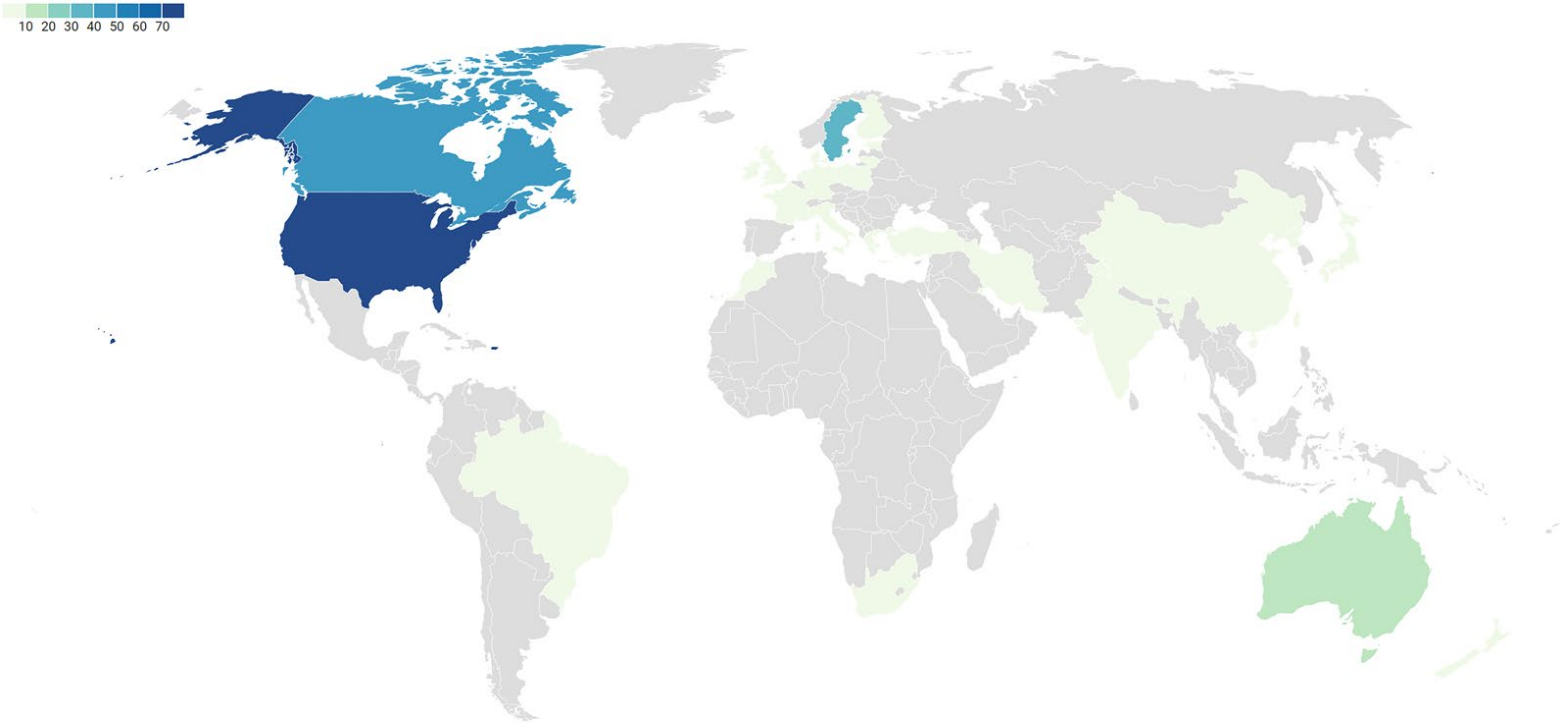
Choropleth displaying the geographical distribution of studies

**Fig. 7:**
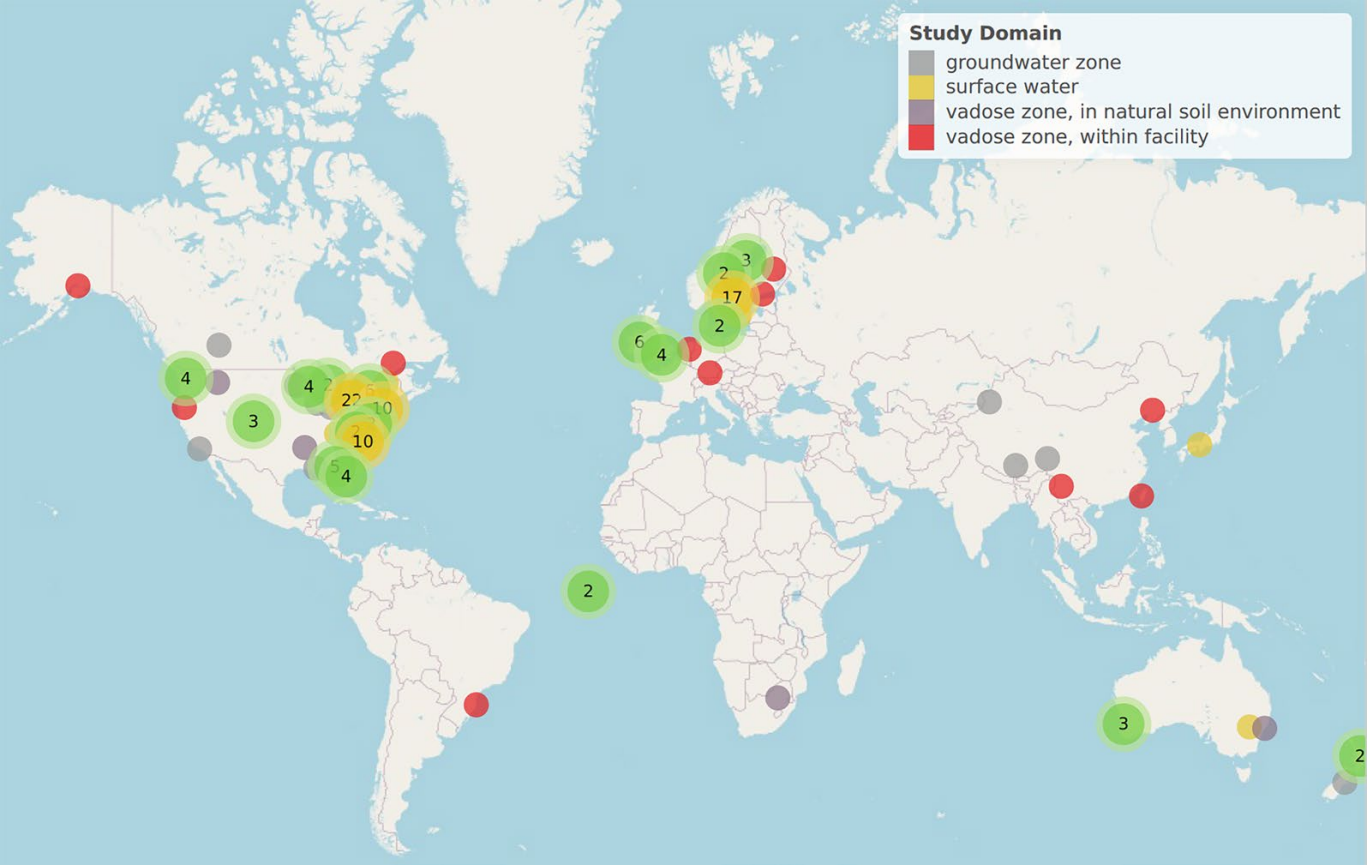
Snapshot of evidence atlas displaying the geographical distribution of field studies. The two studies placed in the Atlantic Ocean lack coordinates. As regards the yellow and green dots with numbers, the colours do not indicate a specific study type. Yellow dots with numbers contain more than 10 studies that appear while clicking on it in the html-based version; green dots contain 2–9 studies that appear while clicking on it. (Yellow dots without a number indicate a single surface water study.)

The bar diagram in Fig. 5 and the choropleth map in Fig. 6 (link: Choropleth), display the number of studies per country. The more precise locations of the field studies, specifically, are displayed in an evidence atlas (see interactive version in Additional file 7 and static snapshot in Fig. 7).

#### Climate zones

**Fig. 8:**
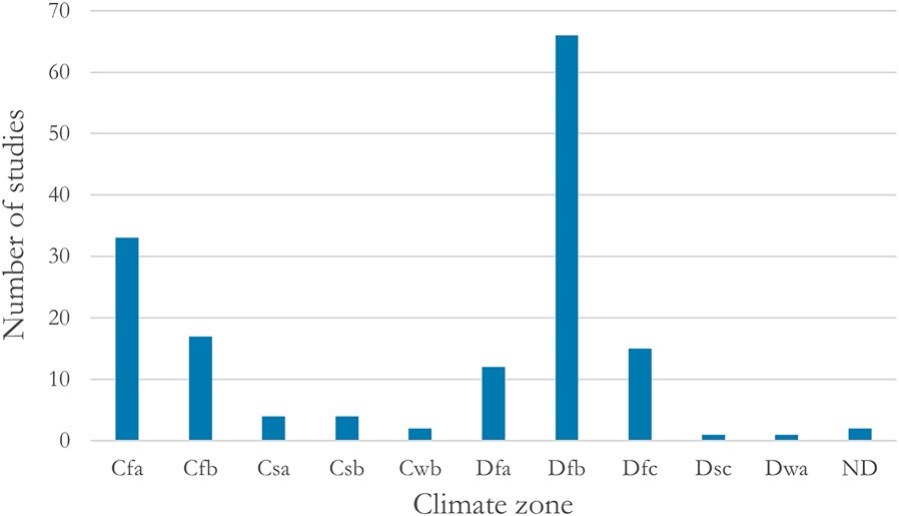
Number of field studies performed in each represented climate zone. ND: no data reported or inferable

A total of 95 field studies were conducted in climate zone D, most commonly in Dfb (n = 66). Sixty field studies were performed in climate zone C, most commonly in Cfa (n = 33) (Fig. 8). It should be noted that in many cases the climate zone was not reported by the authors. In these cases, we have tried to find it out based on the location of the study. However, the reported locations are often less exact than needed, especially when the study is performed close to the limit between two climate zones. That is, the climate zone data should not be regarded totally reliable.

#### Study types

**Fig. 9:**
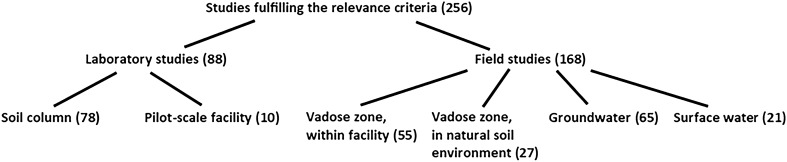
Categorisation of included studies. The numbers in brackets refer to numbers of studies

The studies were categorised into the six different study types mentioned in the data coding section (Fig. 9). First, each study was defined as “Laboratory study” or “Field study”. Laboratory studies were split into “Soil column studies” (LC) and “Pilot-scale studies” (LP), the latter referring to experiments performed with lysimeters constructed to simulate infiltration beds (but in an indoor environment). Field studies were further classified based on where in the soil system the measurements were conducted, i.e., in the vadose zone, within facility (VW); in the vadose zone, in natural soil environment (VN); in groundwater zone (G); or in surface water (S). It could be argued that studies performed within facilities should have been excluded since our review question is focused on the natural soil environment between the facility and the recipient. However, we judged these studies to be analogous to laboratory soil column studies, assuming that the soil material within the facilities is natural.

As a consequence of this classification, one article may include several study types. Those articles were split into two (or more) partial studies and each partial study was assigned a unique study ID and was coded separately in the data base. Hence the sum of studies is not the same as the number of included articles.

A note on soil-based field studies: although the aim of most studies within each of these study types (Vadose zone, within facility; Vadose zone, in natural soil environment; and Groundwater, respectively) is to evaluate P retention in that specific part of the system, this is not always the case. For example, studies in which the measurements were performed on septic tank effluent and shallow groundwater are most often counted as groundwater studies, although they are primarily designed to evaluate what happens in the vadose zone.

### Mapping the quality of studies relevant to the question

The presentation of the evidence base is structured around the six study types defined in the previous section.

As declared in the systematic map protocol [15], a conceptual model is used as a foundation for the discussion. The conceptual model for the processes involved in soil retention of P, and the subsystems in which different processes occur, is described in the section "The processes involved in soil retention" above, and shown in Fig. 1. In the following presentation of the evidence base, modified variants of Fig. 1 are used to clarify where in the system the studies of the different (field) study types have been performed.

The presentation of each study type is structured around three sections: First we describe the evidence base of each category in general terms, including the methods used to evaluate P retention (for study types: LC, LP, VW, VN and G) and methods used to infer possible impact from OWTSs (for study type S). Then we present some general observations regarding the internal validity and reporting quality of the studies of respective study type. Note that this is done from an overall perspective, since we have not performed a critical assessment of each included study. After that we discuss if, and in that case how, the study type might contribute to decision making. This third part focuses on limitations as well as possibilities given by the evidence base. That is, we discuss the limitations that characterise each study type when it comes to using it as the basis for conclusions about the focal question, but also what other knowledge, relevant to stakeholders—and with connections to the focal question—that might be extracted from the research (possibly through systematic reviews).

As mentioned above, one article may include several partial studies, sometimes of different study types. Moreover, each study may include more than one studied facility. Note that in the following review, focus is, variably, on “article”, “study”, “studied facility”, “studied filter” or “case” depending on what is most informative and doable for the specific parameter discussed. It should also be noted that the same facility may have been studied several times and/or with different research questions (in separate articles). In the review below each such occasion is counted as a separate case.

Studies performed on OWTSs that were (or had been) in use for wastewater treatment are categorised as “observational”. Studies performed on soil plots or on facilities set up solely for the purpose of research, and where manipulative experiments were carried out, are categorised as “experimental”.

#### Studies performed in the vadose zone, within facilities

##### General overview of the study type

**Fig. 10:**
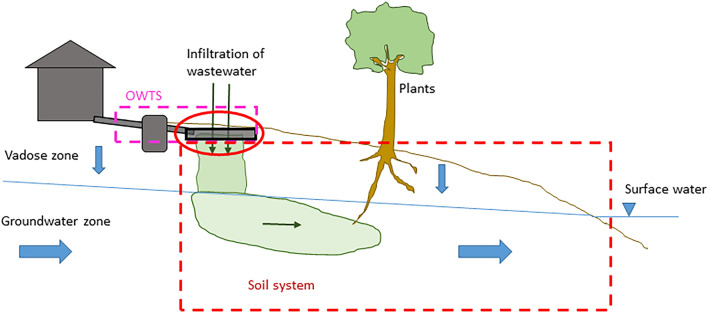
The red ellipse shows which part of the system that is in focus in studies performed in the vadose zone, within facility. The red, dashed line indicates the limits of the complete natural soil system between facility and recipient, that is, the system of primary interest

Fifty-five field studies performed in the vadose zone, within facility (Fig. 10), fulfilled the eligibility criteria and are included in the systematic map. These studies were performed outside of the focal soil system, but they are included since they were performed in natural soil or sand filters (although the soil or sand might have been brought there from another location).

Most of the 55 studies (n = 42) were observational, examining the fate of P within altogether 129 separate facilities/filters^5^ that were in use for wastewater treatment. Thirteen studies were experimental, examining the fate of P within altogether 34 facilities/filters set up for the purpose of research. That is, in all 163 separate filters were studied.

**Fig. 11:**
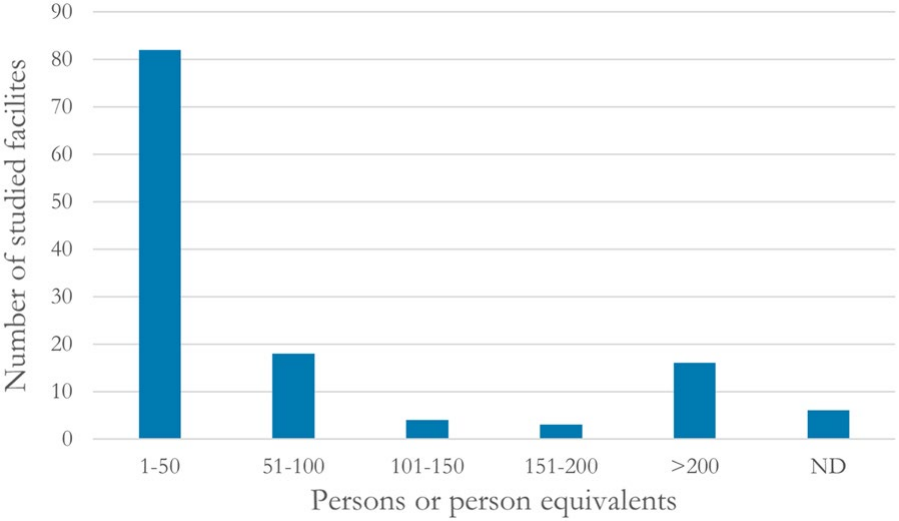
Number of studied facilities within five size categories. ND: no data reported

Most of the 129 studied facilities of the first category were small-scale, serving (or were designed to serve) just a few persons or households. Eighty-two facilities were serving (or were designed to serve) up to 50 persons (Fig. 11).^6^ The biggest studied facility was designed to serve 350 person equivalents.^7^

The hydraulic (wastewater) loading rate was reported or inferable based on available data for 105 studied facilities; not reported or inferable based on available data for 57 studied facilities; and it was not reported, and unclear if it was inferable, for one studied facility.

Most filters consisted of sand or gravel, sometimes in combination with other natural soil materials. Peat was used in 11 of the facilities that were described separately. Other examples are silt loam (used in one facility), and meadow brown soil (used in one facility). For 18 of the facilities that were described separately, the composition of the filter was not reported.

**Fig. 12:**
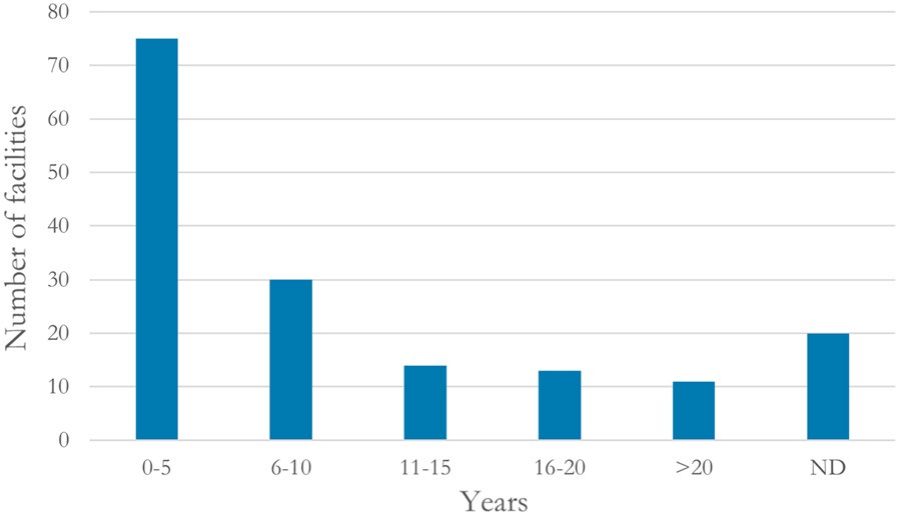
Duration of wastewater exposure. ND: no data reported

Many (75 out of 163; 46%) studied filters had been exposed to wastewater for a fairly short period of time (0–5 years), whereas only 11 facilities (7%) had been in use for more than 20 years. Data on the duration of wastewater exposure was lacking in 20 cases (12%) (Fig. 12).

In 51 out of the 55 studies, the P retention was evaluated by comparing dissolved P in the leachate from the facility with that of the influent from the septic tank. In the remaining cases, P retention was estimated by determining the accumulated P in the filter bed and compare it with the cumulative P load from the septic tank (4 studies). As for the former type of study, total P was determined, sometimes in combination with phosphate phosphorus, PO_4_-P (using the molybdate-blue or equivalent method) in 43 studies; however, in 8 studies only PO_4_-P was studied (Table 3). These two methods can lead to slightly different results as total P may include organic P and P-containing colloidal/particulate materials that may be removed by the filter by mechanical filtration rather than by chemical or biological processes [24, 25].

**Table 3:** Method to evaluate P retention in soil/sand filters within facility

General approach	Method	Number of studies
Determining dissolved P in influent and leachate	Dissolved total phosphorus, TP, in septic tank effluent and after filter	32
	Dissolved PO_4_-P + TP in septic tank effluent and after filter	11
	Dissolved PO_4_-P in septic tank effluent and after filter	8
Comparing accumulated P with cumulative P load	TP and oxalate-P in filter compared to control	3
	Oxalate-P in filter compared to control	1

##### General observations regarding internal validity and reporting quality

Only one study used spatial replication: the experimental study of Mechtensimer and Toor [14]. When several systems were studied within the same study, the systems differed when it comes to soil properties and/or other relevant factors. This undermines the possibility of robust conclusions to be made about the soil retention of P, and about the factors affecting it, and possible confounders.

Correction of dilution effects, resulting from infiltrating rainwater or from leakage from surrounding soils, may be important to get correct P retention data, particularly for filters subject to low hydraulic wastewater loads (< ≈ 10 L m^−2^ d^−1^). To estimate dilution effects, four authors used estimated rainfall recharge at the site, while four others used chloride (Cl^−^) as a tracer. However, most studies did not report any dilution-corrected P retention data, although Cl^−^ in influent and leachate is sometimes measured, making it possible to calculate it.

Another uncertainty of the method is that dissolved P in the influent and leachate can vary substantially over time, leading to large differences in the calculated P retention. For example, in four of the systems studied by Nilsson et al. [25], the P retention was > 90% at one sampling occasion and negative (i.e., higher dissolved P in the leachate than in the influent) at the next sampling occasion. In other words, an individual measurement represents, at best, a “snapshot” of the P retention, which is often unlikely to represent the long-term P retention of the filter. Therefore, repeated measurements are needed for more reliable estimates of P retention. Of the 51 studies that quantified P retention by measuring dissolved P in influent and leachate, 9 studies contained data for only 1 or 2 sampling occasions, and an additional 11 studies used between 3 and 10 sampling occasions. 22 studies used > 10 sampling occasions, whereas 9 studies did not provide this information.

As concerns the method using accumulated P in the filter bed to determine long-term P retention, an advantage is that this gives an integrated measurement of the P retention taking place during the lifespan of the filter. However, to use this method, very good estimates are required for both the cumulative P load onto the filter and the total or oxalate-extractable P of both the filter bed and of a control (e.g., the unused filter medium, to account for its initial P concentration). Such data are often not known or provided by authors. For example, for one of the filters (Luvehult) studied by Eveborn et al. [11], the authors calculated a long-term P retention of more than 100%, an obvious impossibility. This probably resulted from less precise estimates of one or more of the above-mentioned parameters.

For both approaches to quantify P retention in filters, the cumulative P load (expressed as g P m^−3^ material) is potentially an important metric. To correctly estimate it, the following data need to be available: the hydraulic load of septic tank influent; the mean dissolved P concentration of the influent; and the dimensions of the filter bed. For 85 of the studied facilities, such information was presented, while necessary data were missing for 78 of the studied facilities.

Because P retention in the filters is expected to be dominated by sorption onto Al and Fe (hydr)oxides [10, 11], the filter media have a finite capacity to adsorb P. In order to evaluate different filter media and compare results, the following information is needed: the concentration of oxalate- or dithionite-extractable Al and Fe, the pH in the filter material, and the duration of wastewater load. However, the concentration of oxalate-extractable Al was only reported for 21 of the 163 studied filters and the concentration of dithionite-extractable Al was not reported for any of the filters. Additionally, the concentration of oxalate-extractable Fe was reported for 16 filters while the concentration of dithionite-extractable Fe was not reported for any of the studied filters. The pH value of the soil material was reported for 11 filter beds. The fact that a large part of the studied filters were exposed to wastewater for a short period of time (Fig. 12) makes the evidence base weak when it comes to evaluation of different sand/soil filter materials in the long term, which is vital for robust conclusions [10].

##### How might this study type contribute to decision-making?

The aim of the studies within this category was most often to evaluate the purification efficiency of sand/soil filters when it comes to different substances occurring in wastewater (including phosphorus). That is, the soil retention of P was undoubtedly in focus. However, although the soil material within the filter was “natural” (i.e., not amended as regards P adsorption capacity), the retention results obtained from this kind of studies could not be transferred to knowledge about the complex natural soil system between the facility and recipient waterbodies without caution.

As noted above, a decrease of P retention (within the sand/soil filters) with an increased cumulative P load is expected, which was also shown in some of the included studies. Clearly, it would be relevant to stakeholders to get a more elaborate knowledge about the P retention capacity within natural sand/soil filters over time (that is, with an increased cumulative P load), and about what factors that affect the P retention capacity. This could theoretically be the topic of a systematic review. However, our assessment is that the current evidence base could not provide any firm, general answers to this question, given the shortcomings discussed above.

#### Field studies performed in the vadose zone, in natural soil environment

##### General overview of the study type

**Fig. 13:**
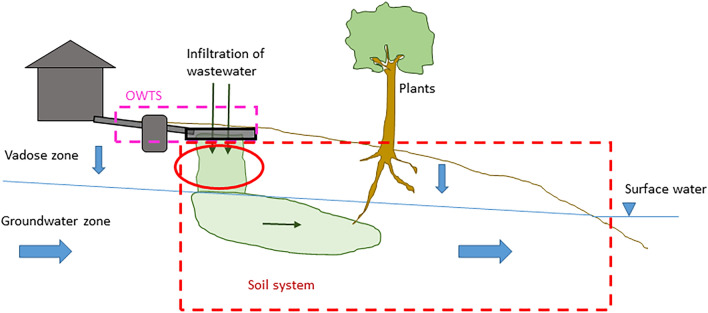
The red ellipse shows which part of the system that is in focus in studies performed in the vadose zone, in the natural soil environment. The red, dashed line indicates the limits of the complete natural soil system between facility and recipient, that is, the system of primary interest

Twenty-seven studies performed in the vadose zone, in the natural soil environment (Fig. 13), fulfilled the eligibility criteria and are included in the systematic map. These studies were—contrary to the studies of the previous category—performed within the focal soil system, although in a limited part of it. Nineteen studies were observational, including in all 75 facilities. Eight studies were experimental, including in all 30 experiments. Three of these studies used replication. In one case, measurements were performed immediately below the vadose zone, in shallow groundwater. This study is included in this category as it aimed to provide information on the P removal in the vadose zone.^8^

Most studied “true” (that is in use for wastewater treatment) facilities (42 out of 75) within this category were small-scale, serving just one house/family. Two were somewhat bigger; one of those were serving 115 persons and the other one 59 dwellings. The size of twenty facilities were more unclear: three were serving “a medical group home”, one was serving "a hotel”, one "a motel”, one "a trailer deport”, one "a school”, two "a resort” and 11 a seasonal use campground”. In 11 cases, data on numbers of persons/households connected to the facility were missing. Hydraulic (wastewater) loading rate (volume/area/time) was reported or inferable based on available data for 76 of the facilities/experiments and not reported or inferable in 29 cases.

In most cases, septic tank effluent had been infiltrated directly into the soil. However, in two of the studies, the septic tank effluent was first treated in a sand filter before soil infiltration.

**Fig. 14:**
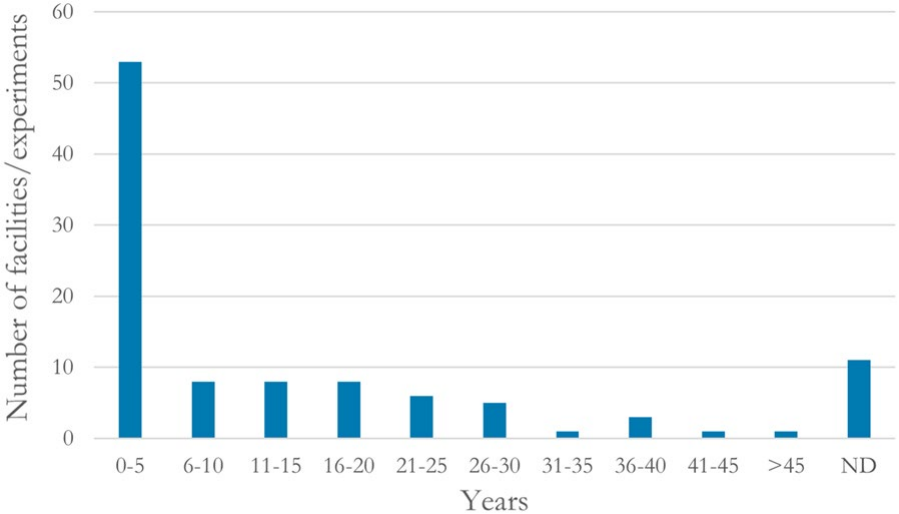
Duration of wastewater exposure. ND: no data reported

A fairly short period of wastewater exposure (≤ 5 years) was reported for 53 facilities/experiments, but 17 facilities had been in use for more than 20 years. Data on duration of wastewater exposure was lacking in 11 cases (Fig. 14).

Similar to the “Within system studies”, in most cases soil P retention had been evaluated by comparing dissolved P in the pore water (typically using a lysimeter) with that of the influent from the septic tank and/or from a sand filter (26 out of 27 studies). In one study, emphasis was instead put on determining the spread of the P plume within the unsaturated zone [26]. In 2 Australian studies, porewater studies were combined with an analysis of the accumulated inorganic P in the soil profile [27, 28].

##### General observations regarding internal validity and reporting standard

Most studies were observational, studying the P flow beneath specific in-use facilities. Eight studies were experimental. Three of those used replication. However, most often, when several systems were studied within the same study, the systems differed when it comes to soil properties and/or other relevant factors. This undermines the possibility of robust conclusions to be made, within and across studies, about the factors that affect P retention.

Of the 26 studies that compared dissolved P in the porewater with that of the septic tank effluent, only two accounted for dilution using the Cl^−^ method, an effect that was sometimes considerable. This calls for caution when interpreting P removal reported for this group of studies. Another factor to consider when interpreting the results is the age of the systems. Fifty-three out of the 105 studied facilities/experiments had been in operation/lasted for 5 years or less, and only 17 studied facilities were ‘old’ (> 20 years) (Fig. 14).

The retention of P in the soil is dependent on chemical conditions, e.g., pH and, as mentioned above, on the amount of Fe and Al (hydr)oxides. However, only 7 studies reported the pH of the soil unaffected by wastewater (prior to the exposure or in a control soil nearby), and no single study reported results for oxalate- or dithionite-extractable Fe and Al.

For the reasons stated above, most of the studies within this study type are likely only to give an indication of the extent of soil retention in the natural environment in the vadose zone. Further, the considerable differences in P removal observed may not always be possible to explain. To what extent these differences are due to different soil properties, different hydraulic loads, or different methodological approaches is poorly, if at all, known. Unfortunately, the evidence base is not sufficient to provide consistent clues.

##### How might this study type contribute to decision-making?

The unsaturated zone beneath drainfields is part of the focal soil system. What happens here has a bearing on the total retention of P occurring between the facility and the recipient waters. A deeper understanding of the P retention occurring in this part of the system would hence be valuable to stakeholders. For example, more detailed knowledge would be needed about the timescale for a given soil to reach its capacity to retain P under the prevailing water chemical conditions. However, most studied facilities had not been exposed to wastewater long enough to enable answering such questions. Specifically, all replicated studies evaluated facilities/soil plots that had been exposed to wastewater for 3 years or less. Moreover, as described above, the reporting was generally deficient, making it difficult to disentangle the factors behind the considerable differences in P removal observed in the included studies. Consequently, our assessment is that the evidence base is insufficient for allowing general conclusions about the P retention occurring in the vadose zone beneath OWTSs. A systematic review would be of limited value; more effort should be put on primary research that is well-conducted (replicated) and adequately reported.

#### Field studies performed in groundwater zone

##### General overview of the study type

**Fig. 15:**
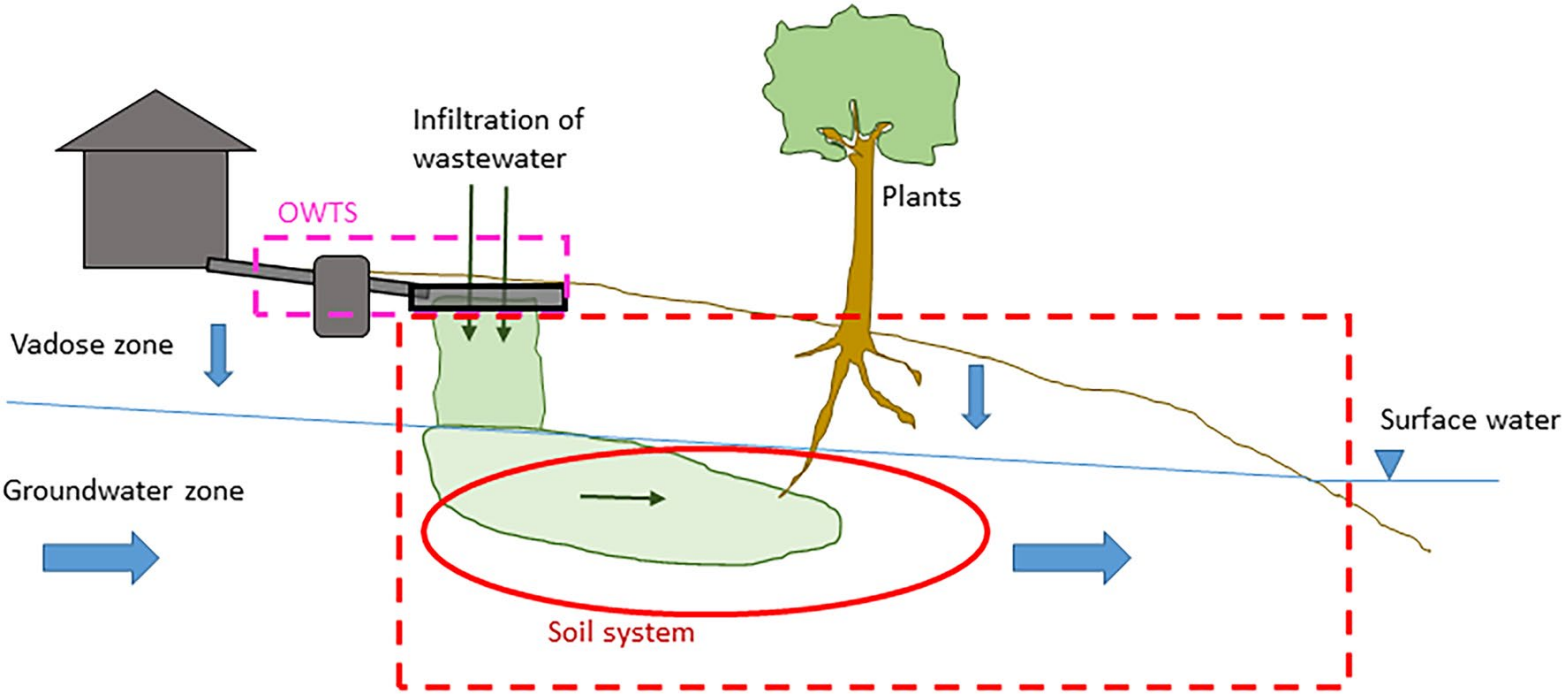
The red ellipse shows which part of the system that is in focus in studies performed in the groundwater zone. The red, dashed line indicates the limits of the complete natural soil system, that is, the system of primary interest

Sixty-five field studies performed in the groundwater zone (Fig. 15) fulfilled the eligibility criteria and are included in the systematic map. Most of these are case studies of one or several sites where the groundwater was, or was suspected to be, impacted by specific OWTSs. In sixteen of the included studies, groundwater in areas with several OWTSs was investigated. Three studies were experimental and one of those was replicated.

A majority of in-use or previously in-use OWTSs that were studied separately (n = 191) were small-scale, serving one or a few households (up to about 50 persons or person equivalents in 119 cases, and between 50 and 250 persons or person equivalents in eight cases). In 35 cases the facilities were serving some kind of public or commercial property (examples are “a campground”, “a school”, or “a hotel”). For 29 facilities this information was missing. In the sixteen studies where groundwater under areas with several OWTSs was in focus, the number of OWTSs in the area was not reported in nine cases. In the remaining studies the number of OWTSs varied between eight and more than 400.

The wastewater loading rate (volume/area/time) was reported or inferable for 65 facilities or experimental plots (out of 198).

**Fig. 16:**
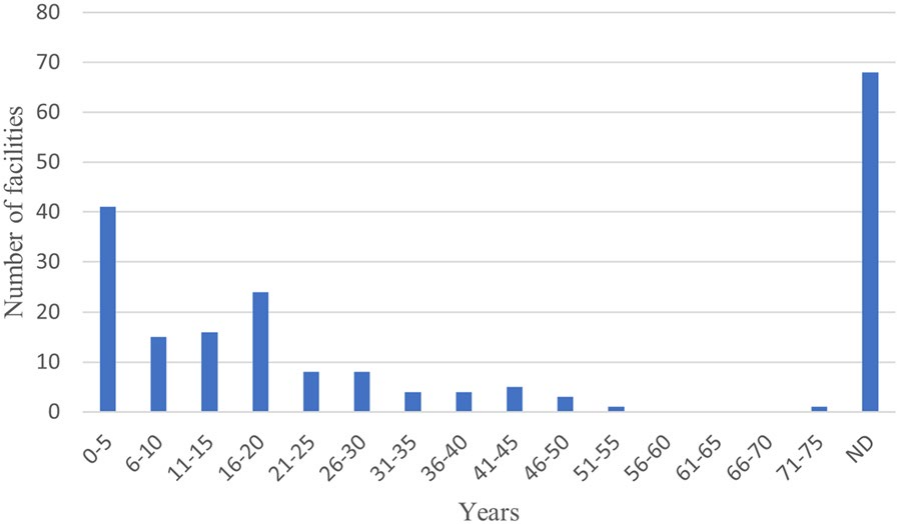
Number of studied facilities per age group (years of wastewater exposure). ND: no data reported

As regards the age of studied OWTSs, data were missing for 68 of the facilities that were studied separately. 56 facilities were 10 years old or less. 34 facilities were older than 20 years (Fig. 16). In the sixteen studies where groundwater under areas with several OWTSs was in focus, the age of OWTSs was reported in three cases: in one of those, many facilities were reported to be more than 20 years old, in the second one, the facilities were between 10 and 75 years old, and in the last one, they were between 20 and 40 years old.

For most of the study sites the soil was described as a sandy material (sand, loamy sand, sand loam etc.), but also sites with predominantly silty materials (silt, silt loam etc.) and finer material (clay, clay loam etc.) were represented. In some cases, there was information about the existence of underlying bedrock or clay, but in none of the study sites, transport and P retention in fractured rock was investigated.

The aim and focus of the studies varied. One group of groundwater studies investigated the impact on groundwater from nearby OWTSs. Primary focus of these studies was not necessarily P. It could rather be, e.g., pathogens or nitrogen, but P concentrations in the groundwater was reported as well, most often compared to background levels. These studies typically did not investigate the transport and retention of P in the groundwater zone in detail or lacked enough data to do so. They often strived to relate the P concentrations in the groundwater to the distance to the OWTS, but often there was not enough or detailed enough data to separate the P retention in the vadose zone from that in the groundwater zone. So, the observed outcome was the combined effect of processes occurring in the vadose and in the groundwater zone, and the main focus was in this case on assessing the status of the groundwater in the presence/vicinity of an OWTS. When measurements have been conducted just beneath the groundwater surface with a very small migration distance in the groundwater zone, this type of study, in fact, provides more information about the efficiency of P retention in the vadose zone than about P retention in the groundwater zone. In some cases, the presence of a dissolved inorganic nitrogen (DIN) plume and simultaneous absence of a P plume, indicated that P from the OWTS had been attenuated in the vadose zone or immediately when entering the groundwater zone [29].

Another group of groundwater studies investigated the evolution of P plumes in groundwater originating from OWTSs. Some studies just characterised the shape and size of the plume, without being able to say anything about how long it took to develop to that size or how long the P takes to go from source to sink. Some of these studies, however, evaluated the overall P retention and P migration velocity in the groundwater zone and some reported a P retardation factor. The P retardation factor describes how much slower the P migrates compared to the groundwater. Typically, a very large number of measurement points (many multi-level wells) are needed to really delineate a plume and assess migration velocity and retardation, and there are relatively few such studies. For example, Harman et al. [30] used 400 sampling points to delineate a PO_4_-P plume that had migrated 75 m in 44 years at a velocity 60 times slower than the groundwater flow velocity (retardation factor of 60).

Several of the groundwater studies also attempted to investigate the mechanisms of P retention in the groundwater zone. In particular, some studies strived to evaluate if the P retention is due to reversible adsorption causing retardation of the P plume or if precipitation of P into minerals is causing a more long-term removal of P from transport with the groundwater. This question is potentially important and appears to depend on site-specific parameters and conditions [5]. Some studies also investigated how P plumes in groundwater reach and cause P emission to surface water, but with less detailed data on the P in the plume and plume processes [29, 30].

##### General observations regarding internal validity and reporting standard

The P retention in the groundwater zone is strongly dependent on a number of environmental parameters, including properties of the soil (or rock), pH, mineral composition etc. (as described above in the conceptual model; Fig. 1). We found that the information about these parameters was most often deficient, or even lacking. This makes it is difficult to generalise the findings to locations other than the specific sites where the studies were conducted, because there is confounding from many undescribed contextual variables.

The groundwater studies comprise the study type that takes most of the complete soil system into consideration and could hence be regarded the most promising when it comes to the possibility to answer the overarching question. However, a prerequisite is that the studied sites have been exposed to wastewater for a sufficiently long period of time, and that the time of exposure is reported. As described above, the age of studied facilities was lacking in 68 cases, and 56 studied facilities were 10 years old or less (Fig. 16). However, 34 facilities were older than 20 years, and among these there were some interesting, reasonably well-reported studies, in which the extension and expansion of the P plume over time was delineated and described. Although also these studies are case studies, and hence ungeneralisable, they present valuable examples.

##### How might this study type contribute to decision-making?

Long-term studies of P plumes in groundwater [e.g., 30, 5] provide an opportunity to study the P retention and transport velocity towards recipients over relevant time scales. There are not enough studies to draw general conclusions, but nevertheless these studies can serve as useful examples representing the specific site conditions in the studies. A synthesis of all groundwater P plume studies would not provide the complete picture of P retention in the groundwater zone but could provide valuable information and examples valid for certain conditions.

The evidence base suggest that P retention mechanisms vary in strength and that different mechanisms may dominate the P retention in the groundwater zone under different conditions, depending on factors such as soil type, mineral composition, groundwater chemistry etc. For some specific conditions, the evidence base may be extensive enough to build models of the dominating P-retention processes and make model predictions of P transport and retention at sites where these conditions apply. However, also in cases (subject to model prediction) where the general site conditions (e.g., soil type) are represented in the evidence base, there can be large variations in site-specific parameters that influence the resulting P-transport and retention. Unless the site-specific parameters (including their spatial variation) are known in great detail, any model prediction will likely be associated with large uncertainties. This systematic map has not investigated to what extent the parameters governing the P retention processes can be generalised to typical hydrogeological conditions in Sweden (or to other boreal, temperate environments). A larger evidence base would be needed to evaluate model-prediction accuracies for a broad range of environmental conditions.

#### Surface water studies

##### General overview of the study type

**Fig. 17:**
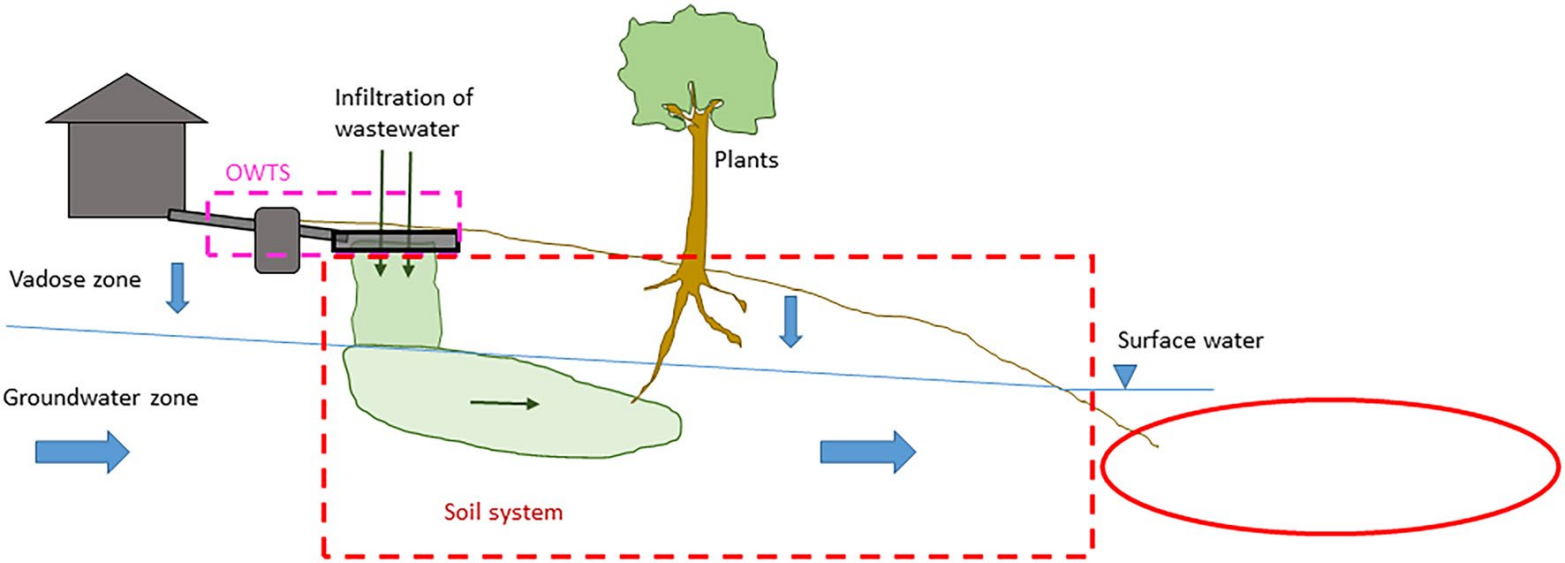
The red ellipse shows which part of the system that is in focus in studies performed in surface water. The red, dashed line indicates the limits of the complete natural soil system, that is, the system of primary interest

Twenty-one surface water monitoring studies (Fig. 17) fulfilled the eligibility criteria and are included in the systematic map. The rationale for including this kind of studies was that if it can be concluded that OWTSs cause raised P concentrations in surface waters nearby, it could also be concluded that the soil retention is not efficient “enough” under prevailing conditions (and the other way around). However, to make such conclusions the contribution of P from soil-based OWTSs, specifically, must have been sorted out from other potential P sources. Many surface water monitoring studies was excluded since they based their calculations on estimations, rather than on measurements, of soil P retention. Other studies have been excluded since they based their calculations on standard values of P released per household, presuming that all P reaches the recipient without retention. A third category of surface water studies have been excluded since the evaluation of impact from OWTSs was deemed to be too speculative.

The duration of wastewater flow was not reported in most of the included surface water studies. In most cases, there were several OWTSs in the catchment area, probably of diverse ages. However, in two studies (in all five sites) the ages of the OWTSs were reported, and the studied OWTSs were in all these cases old (30–60 years).

**Fig. 18:**
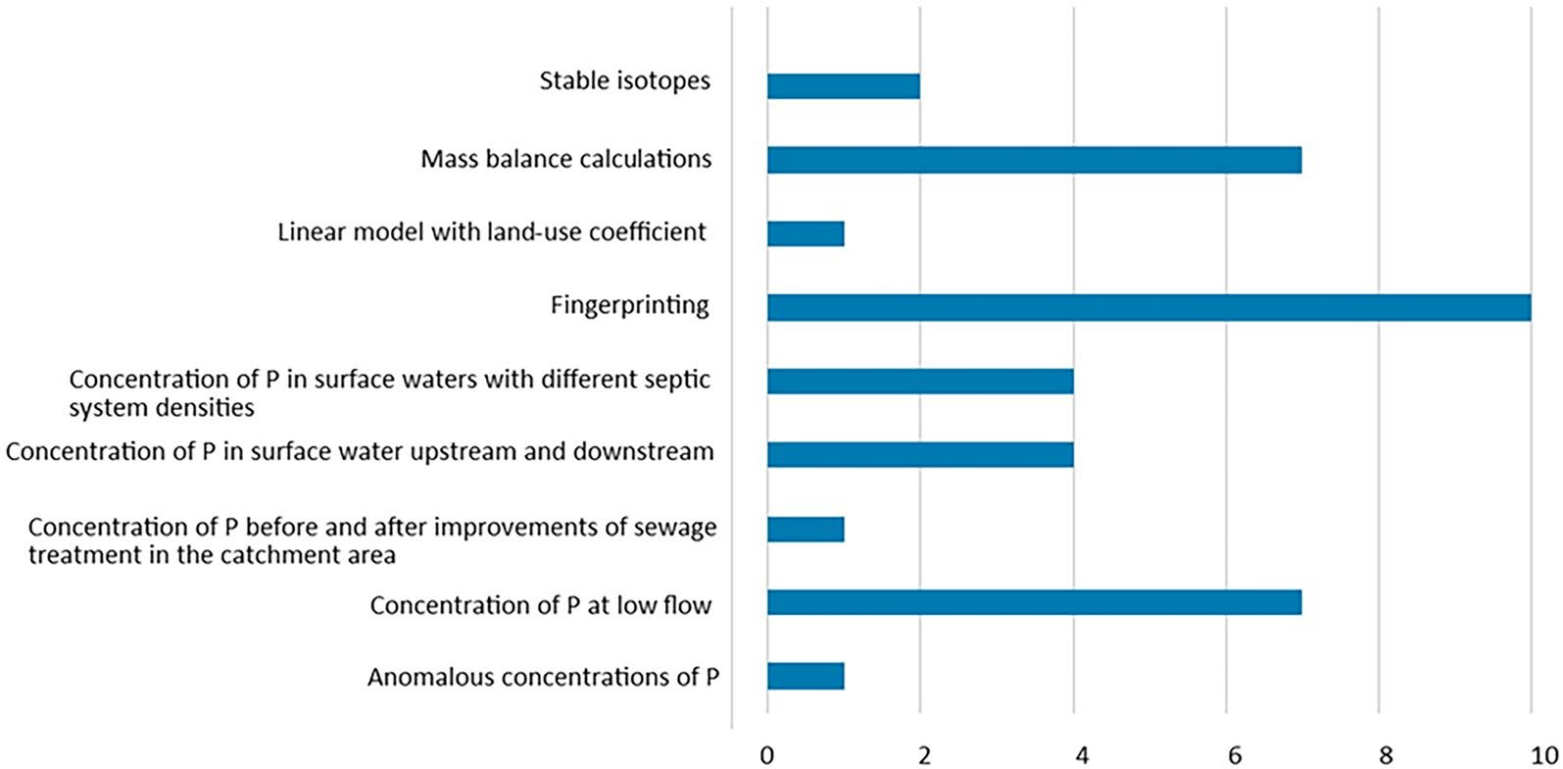
Number of studies using respective method to evaluate impact of OWTS on surface water. Several methods may have been used in the same study

Several methods were used to infer the possible impact from OWTSs on surface waters. The number of studies using respective method is shown in Fig. 18. Often more than one method was used in the same study. Fingerprinting, in which the presence of compounds other than P is analysed to indicate an effect of wastewater (e.g., NO_3_-N, fecal coliforms, sucralose, caffeine), was the most common method, used in ten studies. Fingerprinting was used in some studies as a complementary method to other methods, as it alone does not prove migration of OWTS-derived P into the surface waters. The next most common methods were measurement of the rise in P concentration under low-flow conditions, and mass-balance calculations, respectively, used in seven studies each. The first of these methods uses the assumption that the relative contribution from septic systems is highest under low-flow conditions. Mass-balance calculations involve the estimation of P loads to soil from septic tanks in a specific area, and a comparison with the transport of P in the surface water systems that drain this area. In four studies, it was investigated whether the P concentration in surface water was affected by comparing two different recharge areas, one with a high septic system density and one with a low one. Another method that was used in four studies is comparison of the P concentration upstream and downstream of the discharge from septic systems.

##### General observations regarding internal validity and reporting quality

Many of the included surface water studies suffer from methodological issues. Simply put, there is no easy and straightforward way to assess the impact of soil-based OWTSs on surface water. To get some idea of the general internal validity of included studies, each surface water study was assessed based on (1) the possibility to isolate the effect of OWTSs (yes/no/partly), (2) the comparison with control (without OWTSs) (yes/no/partly), and 3) the contribution of poor systems (i.e., without soil) to the effect (yes/no/uncertain).

The assessment was that it was possible to isolate the effect of OWTSs only in five out of 21 included surface water studies, partly possible in 14 studies and not possible in two studies. Only six studies had used a control (without OWTSs) for comparison. In one study this had been done at least partially, and in 14 studies no control without OWTSs had been used. One recurrent issue was the possible contribution of poor OWTS systems, i.e., systems without soil infiltration (such as so called “sand filters” that discharge directly to surface water). In four of the included studies, there were such poor systems in the catchment area, making it hard to isolate the impact of soil-based OWTSs. In 14 studies, contribution from poor OWTSs could not be ruled out. Only in three studies could it be concluded that there was no contribution from poor systems.

Further, the reporting is often insufficient when it comes to soil properties, number and density of OWTSs in the catchment area, the distance to surface water and age of OWTSs.

##### How might this study type contribute to decision-making?

Existing evidence is limited for the purpose of evaluating to what extent, and under what circumstances, OWTSs *generally* have an impact on P concentration in recipients. The assessment is that the number of studies is far too low, given the complexity and variability of the systems, and given the recurrent methodological issues described above. The evidence base probably does not allow for more detailed knowledge about the factors already known to affect the impact, for example soil type, distance to recipient, and number of OWTSs in the catchment area.

Nevertheless, if a systematic review project with this focus is initiated, our relevance assessments should be treated with caution, and our eligibility criteria possibly fine-tuned (for reasons described below, in section "Limitations of the map").

#### Laboratory soil column studies

##### General overview of the study type

Seventy-eight column studies fulfilled the eligibility criteria and are included in the systematic map. These studies can be split into two categories, one in which specified natural soils were investigated with regard to P retention capacity, and the other one in which soil or sand columns were used as reference, and where the primary objective was to investigate the P retention capacity of different kinds of more or less artificial filter materials. The focus of included column studies was not always on P removal; other substances within the wastewater may have been of primary interest although P data were reported as well.

Methods to evaluate soil retention of P in the column studies varied (Table 4). The predominant method was to measure the P concentration in the wastewater before and after infiltration, respectively. However, the data were not always presented in that form, but as, e.g., P removal efficiency (%) or a P breakthrough curve. A P breakthrough curve is a curve describing the concentration after infiltration divided by the concentration before infiltration (C/C_0_) over time. The second most common method was to measure the concentration of P in the soil after infiltration of wastewater and in a control soil (before infiltration or in another virgin soil sample), often at different soil depths. The third most common method was to measure the amount of P applied and leached, respectively (Table 4). In five studies, researchers used two methods.

**Table 4:** Methods to evaluate P retention in soil columns

General approach	Method	Number of studies
Determining P in influent and effluent	Measure concentration of P before and after infiltration	74
Determining accumulated P in soil	Measure amount of P applied and leached	3
	Measure concentration of P in soil after infiltration and compare to a control (virgin soil)	6

**Fig. 19:**
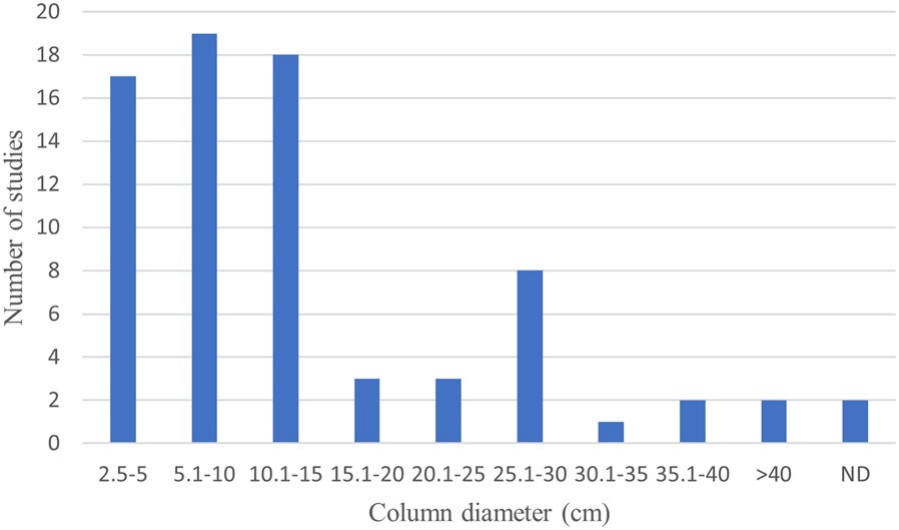
Number of studies using columns with diameters within given ranges. ND: no data reported. (Three studies, using boxes instead of columns, are not included in the diagram.)

**Fig. 20:**
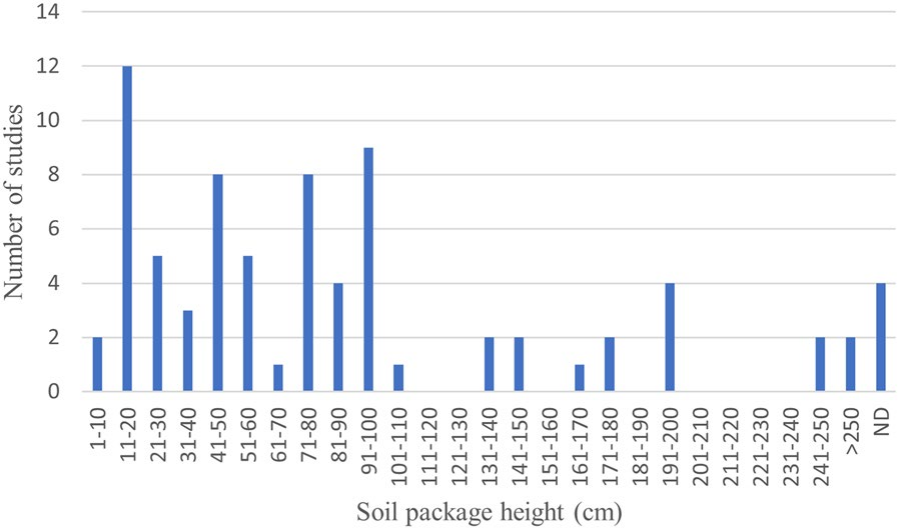
Number of studies using columns with soil package heights within given ranges (cm). Only the largest height in studies where several heights were used is included in the diagram. The ranges indicate flow lengths in most cases. However, this is not completely consistent for the following reasons: (1) it is not always clear whether package height or column height is reported, (2) sometimes there are ports at different heights which means that the flow length is shorter than package heights. ND: no data reported. (Three studies, using boxes instead of columns, are not included in the diagram.)

Among the 78 column studies, a vast range of column sizes were used, with inner diameters from 2.5 to 95.5 cm (Fig. 19), and package heights from 5 to 460 cm (Fig. 20). In three studies boxes instead of columns were used.

**Fig. 21:**
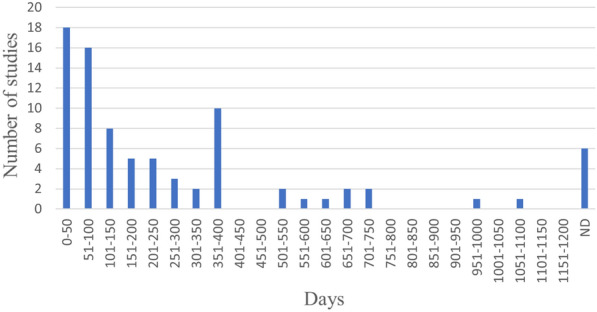
Duration of wastewater flow. If more than one duration was applied within the same study, all durations are included in the diagram. Hence, the sum of studies is greater than the number of included column studies. ND: no data reported

The duration of wastewater load was in most cases a few weeks, but the shortest study was only three days long and the longest study about 3 years (Fig. 21).

**Fig. 22:**
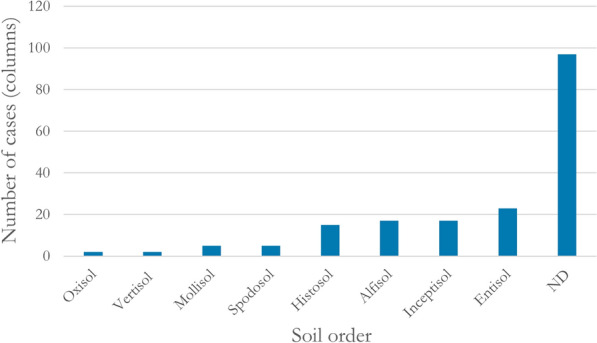
The number of cases using soil of respective soil order. ND: no data reported

Soils of six different soil orders were investigated: Alfisol (used in 17 cases^9^), Entisol (n = 23), Histosol (n = 15), Inceptisol (n = 17), Mollisol (n = 5), Oxisol (n = 2), Spodosol (n = 5), and Vertisol (n = 2) In 97 cases, the soil order was not reported. (Fig. 22)

It is not always clear what kind of wastewater that was used (for example, “domestic wastewater” was a common description) but in at least 33% of the studies, the wastewater was taken from a municipal wastewater treatment plant. In a few of the included studies, relatively “clean” wastewater was used. In some of these cases, it could be suspected that the wastewater was tertiary treated, although this was unclear. In most studies, the wastewater flowed downwards through the columns, but in some studies, upward-flow of the wastewater was practiced.

##### General observations regarding internal validity and reporting quality

As mentioned above, the merit of column studies is the possibility to create a highly controlled environment, which enables the investigation of the impact of specific parameters on the P retention capacity of the soil. Such knowledge is useful not the least while interpreting results from field studies. Important parameters are, in addition to the properties of the investigated soil, the pH of wastewater, hydraulic loading rate and operation time. Chemical and physical soil properties of special importance include pH, the concentration of oxalate or dithionite-extractable iron and aluminium, and particle size. To be able to evaluate specific factors as regards P retention capacity, all relevant parameters must have been adequately measured and reported. Unfortunately, this was not always the case for the included studies. For example, in about half of the cases (53%) no soil order was reported. The concentration of dithionite and/or oxalate-extractable Al and Fe was reported in as few as seven of the studies^10^ (9%). The pH of the soil was reported in 50% of the studies. In 64% of the studies, no information about the fraction of sand, silt and clay, respectively, was reported. In the column studies in which other media (i.e., non-natural soils) were in focus, and a column filled with natural sand or soil was used as reference, the reporting regarding the reference column(s)—the only one(s) relevant to us—was generally most deficient. In these cases, the soil was commonly categorised as a “sand”, and further description was scarce or even lacking. pH of wastewater before infiltration was reported in 67% of the studies, and pH of wastewater after infiltration in 47% of the studies. The hydraulic loading rate was reported, or inferable based on the data reported, in 87% of the studies.

In columns, channels that bypass soil filtration may form. This may lead to preferential flow patterns, making the wastewater flow through the more quickly. Preferential flow patterns may hamper P retention since retention processes will not have the time to occur. This is more likely to be observed under saturated conditions since flow rate is higher during saturated than during unsaturated conditions. Preferential flow patterns also contribute to a smaller contact area between wastewater and soil, where P retention mechanisms take place. The smaller the column is, the more pronounced may these phenomena be. Large-scale columns may also suffer from preferential flow patterns, but the probability is less. Accordingly, studies on large-scale columns generally provide more valid results. However, most columns used in the studies included in this systematic map are to be regarded rather small (Figs. 19, 20).

The chemical composition of the wastewater loaded to the soil materials may also have an impact on the measured P retention. In the column studies included in the systematic map, wastewater from small-scale wastewater treatment facilities such as OWTSs as well as wastewater from large-scale municipal treatment plants had been used. In at least 33% of the studies, municipal wastewater was used. Wastewaters of such origin are often composed of a larger number of different pollutants than a wastewater from a small-scale OWTS serving one or just a few households. This might affect the results, making them less valid for OWTSs. In a few of the included studies rather “clean” wastewater was used. In some of these cases it could be suspected that the wastewater was tertiary treated, although this was unclear. Results from such studies could also be misleading.

The columns in the included studies were reported to be loaded with wastewater for periods ranging from days to years (Fig. 21). Most studies reported periods shorter than 4 months. Depending on the length of the operation period, different P retention mechanisms have the possibility to occur. Results from a column study with short duration, e.g., hours, are difficult to extrapolate to estimates of the efficiency of retention of the soil in the long run. Column studies that run for a period long enough to reach break-through give a more realistic idea of the soil’s P retention capacity. Break-through occurs when the P concentration of the effluent from the column is equal to the P concentration of the wastewater that was fed to it, which happens when the soil is saturated with P. This phenomenon is similar to field conditions where an OWTS has been in operation for several years and the soil is saturated with P. A breakthrough curve (C/C_0_), showing this, was reported in three studies, but P saturation was reached in several cases.

In most studies, the wastewater flowed downwards through the columns, but in some studies, upward-flow was practiced. When column experiments are conducted under saturated conditions, upward-flow is often preferred, as it is commonly assumed that air pockets are more easily displaced, leading to more complete water saturation during the experiment. On the other hand, downward loading of wastewater makes the conditions more similar to field conditions, where wastewater from an OWTS percolates downwards through the soil to the groundwater level.

The experiments were replicated in only 35% (n = 27) of the column studies. Most often one column was used per soil type or per treatment. This means that it is not possible to evaluate the soil retention statistically. Nor can the results be used in meta-analyses.

##### How might this study type contribute to decision-making?

The complexity that prevails under field conditions cannot be replicated in a laboratory soil column setting. Hence, it is not possible to base the knowledge about the retention of P in the natural soil environment solely on results from column studies. The merit of column studies, however, is that they are run under controlled laboratory conditions, which makes it possible to study how specific parameters affect soil retention. Moreover, the possibility to control the actual inflow and outflow of wastewater obviates the dilution problem often encountered in field studies. Accordingly, well-performed and well-described column studies constitute a valuable part of the evidence base. They may provide important complementary information, not the least while interpreting the results from field studies.

It would be possible to conduct a systematic review based on results from soil column studies. However, there are concerns that should be considered before launching such a project. First, given the shortcomings described above, it is unclear how many of the column studies included in this systematic map that would be included in a systematic review, after being critically assessed. The lack of replicates in about 65% of the column studies is a problem since such studies cannot be included in meta-analyses. The insufficient reporting of relevant parameters makes it less probable that there are enough studies to disentangle the significance of specific parameters. The lack of an established reporting standard aggravates syntheses of results. Further, the stakeholder relevance of a systematic review of column studies is not obvious. The outcome of such a review—in case the above-described concerns are not too severe—could be more precise estimates of the impact of different parameters already known to affect P retention. This would probably be more relevant to researchers than to policy and practice decision-makers.

#### Pilot-scale studies in the laboratory

##### General overview of the study type

Ten pilot-scale studies performed in the laboratory fulfilled the relevance criteria and are included in the systematic map. This category of studies can be thought of as being in-between laboratory column studies and studies performed in the vadose-zone, within-facilities. In these studies, some kind of pilot-scale set-up had been constructed using several components such as boxes filled with soil or sand and vegetated boxes. In eight of these studies, sandy materials were used in the filter. One study used peat, and one used loamy soil. All studies measured the concentration of P in the water before and after infiltration, to evaluate the P retention. Secondary treated wastewater from a septic tank was used in only one study; in all the other studies, other types of wastewaters were used, e.g., municipal. The duration of wastewater flow varied between 1 and 275 days.

##### General observations regarding internal validity and reporting quality

An advantage with this type of study, compared to vadose-zone studies in the field, is that the influent wastewater volumes may be more efficiently controlled. Moreover, the possible influence of dilution effects from rainwater can be disregarded. However, none of these studies used spatial replication. Moreover, the reporting is deficient throughout. For example, the soils used as filters are insufficiently described; e.g., none of the studies reports oxalate- or dithionite-extractable Fe and Al. Six of the studies allow cumulative P loads (in g P m^−3^) to be calculated.

##### How might this study type contribute to decision-making?

The study set up varies considerably among studies within this category, making compilation of results difficult. Moreover, the lack of spatial replication and the generally deficient reporting make the studies less valid. These studies cannot not contribute to decision-making in isolation.

## Limitations of the map

### Limitations of the review methods

Firstly, only studies written in English, Swedish, Norwegian and Danish have been taken into consideration. The reason for this is primarily practical: these are the languages mastered by the review team. Although these languages are also deemed to be the most relevant ones given the geographical scope of primary interest of our core stakeholders, there are probably relevant studies written in other languages as well, for example Russian and Polish.

Secondly, we have not screened the reference lists of included articles or relevant reviews in a systematic and exhaustive way, to find articles that had not been found in the main searches. There is no standard best practice on citation chasing, but a more exhaustive screening of reference lists would likely have resulted in some additional relevant articles.

Thirdly, field studies conducted in small-scale facilities but using municipal wastewater have been excluded, which might be regarded inadequate. The reason for this is that the evidence within this systematic map should be valid for soil-based, on-site wastewater treatment systems designed to serve up to 200 person equivalents (in accordance with the definition made by the Swedish Agency for Marine and Water Management). Hence, one of our eligibility criteria (for field studies) was that the studied wastewater must originate from single or groups of households and released to soil; field studies on wastewater from municipal wastewater treatment plants were not eligible. The primary rationale behind this criterion was that the hydraulic loading rate within municipal wastewater plants is of a completely different order of magnitude than that within OWTSs. Hence, the transferability of results from municipal wastewater plants to knowledge about OWTSs is dubious. What we did not foresee while writing the protocol, though, was that there are experimental field studies that were conducted in small-scale facilities but that used municipal wastewater. However, we decided to adhere to the original eligibility criteria but list small-scale field studies using municipal wastewater in an additional, parallel database (Additional file 8), for any interested reader to examine. It should be noted, though, that this database is not necessarily exhaustive since this kind of studies were not the subject of a comprehensive search.

Fourthly, as regards surface water studies, we found it difficult to apply one of the criteria in a consistent way, i.e., the criterion that the contribution of P from OWTSs, specifically, must have been evaluated and analysed. For example, often it is not possible to be completely confident that a given effect is due to the flux of P from OWTSs or from another source. Hence, we have argued that the authors do not have to show *unequivocally* that the contribution of P from OWTSs has been correctly quantified, but it should be evident that some kind of conclusion can be made concerning the role of OWTSs, despite the occurrence of uncertainties. However, this is open to interpretations and, possibly, to inconsistent assessments.

In addition to the limitations described above, it could be suspected that the captured evidence base is not complete, although for reasons beyond our control. The possible impact from OWTSs on surface waters has been a concern for decades, and a vast number of “grey” reports from governmental agencies, consulting firms, and research institutes related to the topic have been produced. It has been challenging to get access to many of these (often old) reports. 192 potentially relevant records (11%) captured by our literature searches, and included after screening at title/abstract level, have not been found in full text, although genuine efforts were made. These references are listed in Additional file 6. Most of these are conference papers, theses, or very old works. We also suspect that grey literature has slipped through our searches, as they are not indexed in the databases, and may be hard to find through “manual” searches. However, this may be regarded as a general limitation of the methodology; we have followed best practices. In summary, it is probable that there are relevant studies that are not included in the systematic map. However, our assessment is that it is unlikely that the conclusions from this systematic map would have been different if we had found these studies.

### Limitations of the evidence base

Stakeholders need valid generic guideline values of the retention of P occurring in the natural soil environment between an on-site wastewater facility and the recipient waters. To be able to determine such values, replicated field studies, performed over complete systems (from facility to recipient), during long periods of time and under different (well-documented) local conditions would be preferable. Although it would be possible, at least in theory, to conduct such studies, none has been found. Instead, almost all field studies are unreplicated, and to be regarded as case studies. Hence, they could not be included in meta-analyses. Typically, they are focused on a specific, limited part of the system, giving a snapshot of the conditions at this specific site at a specific point in time. For the results from these studies to be synthesisable, they must be combined as individual data points, and a very large number of studies performed according to a consistent sampling and reporting standard would be required to be able to investigate the influence of different parameters. These requirements are not fulfilled by the evidence base.

Another limitation of the evidence base is that the duration of wastewater flow in a majority studied facilities is either short (< 5 years) or not reported. This is a shortcoming partly because the retention mechanisms occurring in the soil affect its future P retention capacity. Simply put, from the beginning all P may be adsorbed in a specific part of the system, but in due time the soil becomes saturated with respect to P, after which (almost) no P is adsorbed. Hence, the time perspective is important.

An additional limitation of the evidence base, concerning primarily studies performed in the vadose zone, is that most studies do not report any dilution-corrected P retention data. This is a problem, because correction of dilution effects from, e.g., precipitation recharge may be important to get correct P retention data. In many studies where no correction was made for dilution, there was no information about the reason (i.e., whether there was a lack of data, or whether the system was designed in such a way that dilution did not occur).

Unfortunately, reporting deficiencies are a recurrent problem: there is a large number of methodological details and factors of crucial importance for P retention that is not reported, and there are a lot of descriptions of results without provision of the actual data. Above all, the articles often lack sufficient information about contextual factors of potential significance. For example, the properties of the soils are often insufficiently described. Moreover, methods are often inadequately reported.

## Conclusions

### The character of the evidence base

This systematic map aimed to describe, collate and catalogue literature related to soil retention of P from on-site wastewater treatment systems in boreal and temperate climate zones. It can be concluded that there is a substantial amount of research related to the topic. It can also be concluded that the evidence base is indeed heterogeneous. Some of the variability applies to study setting factors, e.g., the limits of the soil systems under study, focal outcomes, and study designs. This variability is partly an inherent consequence of the broad mapping approach. Other variability applies to conditional factors within the systems under study, such as soil properties, size of OWTSs, and durations of wastewater discharge. This variability could be regarded as a strength of the evidence base since it is a prerequisite for the investigation of possible effect modifiers. Furthermore, the level of detail of the reporting—and what kind of data that is reported—varies to a great extent. Obviously, no reporting standard has been established: there is a noticeable heterogeneity as to what is reported and how, even if the same phenomenon/process/outcome is studied.

Another characteristic of the evidence base is that most of the studies are unreplicated and hence to be regarded as observational case studies. This applies to almost all field studies (97%) (including most experimental field studies [79%]), and to the majority (69%) of laboratory studies (column and pilot-scale).

### Implications for policy/management

Stakeholders need generic guideline values for the retention of P occurring in the natural soil environment between an on-site wastewater facility and the recipient. As noted above, replicated studies, conducted over the complete systems for long periods of time (at least decades), would be preferable to get such estimates through traditional meta-analyses. However, there are no such studies. Although theoretically possible, such a study design would be almost impossible in practice. An alternative could be a great number of case studies conducted over the complete system and for long periods of time, and according to similar study protocols. While there are some such case studies in the evidence base, they do not cover a broad enough range of environmental conditions, and they are too few to draw general conclusions. Accordingly, our primary conclusion is that it is not possible to derive valid generic guideline values of the efficiency of soil retention of P occurring between OWTS and recipient (expressed as, for example, g P/m^3^ soil or % retained P) from available empirical data.

Our second conclusion is that it is not possible to derive generic estimates of the efficiency of the soil retention of P occurring in the unsaturated zone below OWTSs, from available empirical data. Neither does the evidence base offer more elaborate, generally valid, knowledge about the P retention efficiency (within natural sand/soil filters) over time (that is with an increased cumulative P load), which otherwise probably would be highly relevant to stakeholders. Firstly, almost all studies in the vadose zone are conducted without replication. Secondly, very few of these studies report dilution-corrected P retention data, although infiltrating rainwater or leakage from surrounding soils may affect the results considerably. Thirdly, few studies have been performed long enough to be able to evaluate the soil retention of P in the long run. Fourthly, few studies report quantified soil properties relevant for the adsorption of P.

Our third conclusion is that the evidence base does not allow for answering the question of the magnitude of the potential impact of OWTSs on the P concentration in recipients on a general basis and/or under what conditions soil based OWTSs generally do have such an impact. The studies addressing this question are far too few, given the complexity and variability of the systems. Moreover, in most cases (for practical reasons) there are methodological issues, making the causal inference dubious, also in the specific cases.

Although the evidence base does not allow for estimating the efficiency of soil retention of P from OWTSs through traditional meta-analyses, a possibility might be to approach the question through model simulations. Our fourth conclusion is that the evidence base may allow for creating model simulations or prognoses for some specific environmental conditions. However, the practical usefulness of such models would be limited, since the hydrogeological and biogeochemical conditions underground vary considerably, and site-specific information about these conditions are in most cases unknown. There are too few studies to create valid model simulations and prognoses for a broader variety of conditions. The variability in the parameters governing the transport and retention of P in the subsurface, coupled with our limited ability to observe what is underground, is significant obstacle to answering the overarching question.

The primary conclusions relevant to policy and management are listed in Table 5.

**Table 5:** Primary conclusions of relevance to policy and management

Conclusions	Main observations that form the basis for the conclusions
• It is not possible to derive valid generic guideline values for the efficiency of soil retention of P occurring between OWTS and recipients from available empirical data	• There are too few studies conducted over sufficiently long periods of time (at least decades) that cover the complete system from OWTS to recipients
• It is not possible to derive generic estimates of the efficiency of soil retention of P occurring in the vadose zone within or below OWTSs from available empirical data	• Almost all studies in the vadose zone are conducted without replication • Very few studies report dilution-corrected P retention data • Few studies were performed long enough to be able to evaluate the soil retention of P in the long run • Few studies quantify soil properties relevant to the adsorption of P
• The evidence base does not allow answering the question of the magnitude of the potential impact of OWTSs on the concentration of phosphorus in recipients on a general basis, nor under what conditions soil-based OWTSs generally have such an impact	• The studies addressing this question are far too few, given the complexity and variability of the systems • In most cases (for practical reasons) there are methodological issues, making the causal inference dubious, also in the specific cases
• The evidence base may allow for creating model simulations and forecasts for certain specific environmental conditions. However, the practical utility of such models would be restricted	• The hydrogeological and biogeochemical conditions underground vary considerably, and site-specific information about these conditions is often unknown • There are too few studies to create valid model simulations and forecasts for a broader range of conditions

In the following we will outline two feasible systematic review projects. Although the answers these potential systematic reviews might deliver are not the answer to the original question raised by stakeholders, they might still be informative. However, it should be noted that it is unclear how firm conclusions that could be drawn, because of the issues discussed below.

A systematic review based on laboratory column studies might potentially contribute more precise estimations of different factors that have an impact on the P retention capacity of different soils. However, as described above, P retention results from laboratory column studies (and hence results from a systematic review based on such studies) should not be regarded as valid for the natural soil system. Moreover, many of the column studies are not replicated, which means that it is not possible to evaluate the soil retention results statistically. These studies could therefore not be included in meta-analyses. Further, necessary information about important soil parameters is often lacking.

A systematic review based on studies performed in the groundwater zone, aiming to delineate the extension and expansion of the P plume might potentially be valuable. For the purposes of the overarching question (effectiveness of soil retention of P) their data are at a high risk of bias because they are unreplicated and confounded. Moreover, information about important parameters, crucial for the P retention (for example hydraulic loading rate, duration of wastewater load and detailed information about the soil), is often lacking. Nevertheless, a compilation of these studies might help bringing the question one step further by exemplifying how far the P plume may reach in a given time period. However, again, since these studies are case studies, and the number of them insufficient given the complexity and variability of the systems, no general conclusions would be possible.

The overall conclusion is that the evidence related to soil retention of P from OWTSs cannot form a firm basis for decisions made by stakeholders and policy makers. Simply put, almost all P will reach the recipient surface water in due time. Soil retention processes inhibit the P transport, making less P reaching the recipient surface per unit of time, but the efficiency of these soil retention processes remains unclear. Decisions about what requirements to set on OWTSs must be made, but research-based knowledge about the magnitude of soil retention of P is beyond reach and can therefore not contribute effectively to those decisions. That is, the evidence base does not allow for answering the question raised by stakeholders. However, this is an important conclusion per se, not least in view of the fact that research results are often referred to in order to support different opinions on the issue.

### Implications for research

There is a need for more groundwater studies, aimed at delineating the extension and expansion of the P plume downstream from OWTSs that have been in use for long periods (decades). Although these studies are case studies, additional ones would provide valuable examples of the development of P plumes over time, and possibly indicate under what circumstances (such as distance to the recipient) OWTSs may be suspected to be problematic as regards P. For the Swedish context specifically, more groundwater studies at sites with relatively thin soil layers on top of fractured crystalline rock are desirable.

More studies in the vadose zone, both within the facility and in the natural soil environment, are also desirable. Focus should be on facilities that have been in use for long periods of time and effort should be put to design studies that account for dilution. To consider the ability of the soil to bind P, oxalate-extractable iron and aluminium should be measured. Additional such studies could, for example, contribute to better estimations of the time perspective concerning the P saturation process.

There are some observations that lead to conclusions of relevance to future research on a general basis. Firstly, the majority of studies included in this systematic map is observational case studies. Typically, measurements have been conducted in the vicinity of existing, real OWTSs. Also, many of the experimental studies are to be regarded as case studies since there is no spatial replication. This applies to experimental field studies (performed in pilot-scale facilities or experimental soil plots) as well as to laboratory studies. This makes the research results less valid and less useable. Future research should hence strive for truly replicated study designs, when possible.

Secondly, the reporting quality within the research field is often deficient. Although a general improvement over time as regards reporting might be discernible, there is still a need for improvements: more relevant data should be gathered and/or the data/methods should be reported in a more transparent and exhaustive way than is often done. We recommend a reporting and study design standard within the research field be established, to provide recommendations for the reporting of research methods, contextual data, and results. This would improve the possibilities of critical evaluation, comparison, and compilation of study results.

The primary conclusions/observations of relevance to future research are listed in Table 6.

**Table 6:** Primary conclusions/observations of relevance to future research

Conclusions/observations	Comments
• There is a need for additional studies of P plumes downstream from OWTSs that have been in use for long periods of time	• Such studies provide valuable examples of the development of P plumes over time
• There is a need for additional studies performed in the vadose zone below OWTSs, preferably below facilities that have been in use for long periods of time	• Such studies could contribute to better estimates of the time frame regarding the process of P saturation • The studies should account for dilution • Oxalate-extractable iron and aluminium should be measured
• Many studies are not replicated. In future research, study designs should strive for true replication whenever possible	• Unreplicated study designs make research results less valid and less usable; such studies cannot be included in regular meta-analyses
• The reporting quality within the research field is often unsatisfactory. We recommend establishing reporting and study design standards	• This would improve the possibilities of critical evaluation, comparison, and compilation of study results

## Supplementary Information

**Additional file 1.** ROSES form.

**Additional file 2.** Searching for literature.

**Additional file 3.** Benchmark studies.

**Additional file 4.** Excluded studies.

**Additional file 5.** Database.

**Additional file 6.** Unobtainable articles.

**Additional file 7.** Evidence map.

**Additional file 8.** Small-scale field studies using municipal wastewater.

## Data Availability

All data generated or analysed during this study are included in this published article (and its supplementary information files).

## Footnotes

1 There are OWTSs without a soil infiltration step, for example so called package treatment plants. Such systems are not of interest within this systematic map.

2 Wastewater generated in households, excluding wastewater from toilets.

3 See section "Deviations from the protocol".

4 Complete doctoral theses or separate (unpublished) papers from theses.

5 The number of facilities that were studied, and described, separately. In three studies the data were clustered.

6 The numbers are approximate since the sizes of the facilities are estimated in certain cases. For example, if reported as number of families/residences/households or similar, the number has been multiplied by 4 to estimate number of persons.

7 The results should be valid for OWTSs serving up to 200 pe, according to the definition by the Swedish Agency for Marine and Water Management. We set the limit to systems used by/constructed for 400 persons, reasoning that data from facilities of this size should be generalisable for facilities serving up to 200 pe.

8 It should be noted that there is a grey zone as to how to categorise these kind of studies, and there could be inconsistencies.

9 One case = one “experiment”, that is one column (if unreplicated) or one set of replicates.

10 Note that a study may contain several experiments, replicated or unreplicated.

## References

[1] Drizo A. Phosphorus pollution control—policies and strategies. 1st ed. Hoboken: John Wiley & Sons; 2020.

[2] Canter L, Knox RC. Evaluation of septic tank system effluents on ground water quality. Ada, Oklahoma: US Environmental Protection Agency; 1984. Report EPA-600/2–84–107. Ada: US Environmental Protection Agency; 1984.

[3] Speed CD, Fretwell BA, Davison PS. The role of septic tanks in the dissolved phosphorus budget of the Upper River Nar and possible implications for other catchments. Q J Eng Geol Hydrogeol. 2018;52:23–37. 10.1144/qjegh2018-004.

[4] Swedish Agency for Marine and Water Management (SwAM). Havs- och vattenmyndighetens allmänna råd om små avloppsanläggningar för hushållsspillvatten; 2016. HVMFS 2016:17.

[5] Robertson WD, Garda DL. Pyrite oxidation halts migration of a phosphorus plume. Groundwater. 2020;58:27–34. 10.1111/gwat.12877. 10.1111/gwat.12877 30843187

[6] European Environment Agency. Source apportionment of nitrogen and phosphorus inputs to the aquatic environment. Copenhagen: European Environment Agency; 2005. EEA Report 7/2005.

[7] Hansson K, Ejhed H, Widén-Nilsson E, Johnsson H, Tengdelius Brunell J, Gustavsson H, et al. Näringsbelastningen på Östersjön och Västerhavet 2017. Sveriges underlag till HELCOM:s sjunde Load Compilation. Havs- och Vattenmyndighetens Rapport 2016:12. Göteborg: Swedish Agency for Marine and Water Management; 2019. Havs- och Vattenmyndighetens Rapport 2019:20.

[8] Eveborn D. Sustainable phosphorus removal in onsite wastewater treatment [dissertation]. Stockholm: KTH Royal Institute of Technology; 2013. TRITA-LWR PHD 1070.

[9] Olshammar M. Utsläpp från små avloppsanläggningar 2017. Norrköping: Swedish Meteorological and Hydrological Institute (SMHI); 2018. SMED Rapport 6 2018

[10] Eveborn D, Kong D, Gustafsson JP. Wastewater treatment by soil infiltration: long-term phosphorus removal. J Contam Hydrol. 2012;140–141:24–33. 10.1016/j.jconhyd.2012.08.003. 10.1016/j.jconhyd.2012.08.003 22982614

[11] Eveborn D, Gustafsson JP, Elmefors E, Yu L, Eriksson AK, Ljung E, Renman G. Phosphorus in soil treatment systems: accumulation and mobility. Water Res. 2014;64:42–52. 10.1016/j.watres.2014.06.034. 25036667 10.1016/j.watres.2014.06.034

[12] Zanini L, Robertson WD, Ptacek CJ, Schiff SL, Mayer T. Phosphorus characterization in sediments impacted by septic effluent at four sites in central Canada. J Contam Hydrol. 1998;33:405–29. 10.1016/S0169-7722(98)00082-5.

[13] Zurawsky MA, Robertson WD, Ptacek CJ, Schiff SL. Geochemical stability of phosphorus solids below septic system infiltration beds. J Contam Hydrol. 2004;73(1-4):129–143. 10.1016/j.jconhyd.2004.01.003. 10.1016/j.jconhyd.2004.01.003 15336792

[14] Mechtensimer S, Toor GS. Fate, mass balance, and transport of phosphorus in the septic system drainfields. Chemosphere. 2016;159:153–8. 10.1016/j.chemosphere.2016.05.084. 27288645 10.1016/j.chemosphere.2016.05.084

[15] Envall I, Fagerlund F, Johansson Westholm L, Åberg C, Bring A, Land M, Gustafsson JP. What evidence exists related to soil retention of phosphorus from on-site wastewater treatment systems in boreal and temperate climate zones? A systematic map protocol. Environ Evid. 2020;9:22. 10.1186/s13750-020-00205-9. 10.1186/s13750-023-00300-7 PMC11378865 39294785

[16] Collaboration for Environmental Evidence. 2018. Guidelines and Standards for Evidence synthesis in Environmental Management. Version 5.0. (AS Pullin, GK Frampton, B Livoreil & G Petrokofsky, Eds) 2018. www.environmentalevidence.org/information-for-authors.

[17] Haddaway NR, Macura B, Whaley P, Pullin AS. ROSES RepOrting standards for Systematic Evidence Syntheses: pro forma flow-diagram and descriptive summary of the plan and conduct of environmental systematic reviews and systematic maps. Environ Evid. 2018;7(1). 10.1186/s13750-018-0121-7.

[18] Harzing AW. Publish or Perish 2007. https://harzing.com/resources/publish-or-perish. Accessed 19 Nov 2019.

[19] Bramer WM, Giustini D, De Jonge GB, Holland L, Bekhuis T. De-duplication of database search results for systematic reviews in EndNote. J Med Libr Assoc. 2016;104:240–3. 10.5195/jmla.2016.24. 27366130 10.3163/1536-5050.104.3.014 PMC4915647

[20] Bramer W, Bain P. Updating search strategies for systematic reviews using EndNote. J Med Libr Assoc. 2017;105:285–9. 10.5195/jmla.2017.183. 28670219 10.5195/jmla.2017.183 PMC5490709

[21] Beck HE, Zimmermann NE, McVicar TR, Vergopolan N, Berg A, Wood EF. Present and future Köppen-Geiger climate classification maps at 1-km resolution. Sci Data. 2018;5:180214. 10.1038/sdata.2018.214. 30375988 10.1038/sdata.2018.214 PMC6207062

[22] Haddaway NR, Feierman A, Grainger MJ, Gray CT, Tanriver-Ayder E, Dhaubanjar S, et al. EviAtlas: a tool for visualising evidence synthesis databases. Environ Evid. 2019;8:22. 10.1186/s13750-019-0167-1.

[23] Haddaway N, Macura B, Whaley P, Pullin A. ROSES Flow Diagram for Systematic Maps. Version 1.0. (2018). 10.6084/m9.figshare.6085940.v2.

[24] Brandes M. Effect of precipitation and evapotranspiration of a septic tank-sand filter disposal system. J Water Pollut Control Fed. 1980;52:59–75.

[25] Nilsson P, Nyberg F, Karlsson M. Markbäddars funktion: kontroll och utvärdering av markbäddar. Stockholm: The Swedish Environmental Protection Agency; 1998.

[26] Geary PM. Effluent tracing and the transport of contaminants from a domestic septic system. Water Sci Technol. 2005;51:283–90. 10.2166/wst.2005.0377. 16104432

[27] Whelan BR, Barrow NJ. The movement of septic tank effluent through sandy soils near Perth. II. Movement of phosphorus. Soil Res. 1984;22:293. 10.1071/SR9840293.

[28] Whelan BR. Disposal of septic tank effluent in calcareous sands. J Environ Qual. 1988;17:272–7. 10.2134/jeq1988.00472425001700020019x.

[29] Price TL Jr. Factors Influencing Septic Tank Nutrient Transport to Surface Water Bodies in the Vicinity of the Indian River Lagoon, Florida [dissertation]. ProQuest Dissertations Publishing, 2016.

[30] Harman J, Robertson WD, Cherry JA, Zanini L. Impacts on a sand aquifer from an old septic system: nitrate and phosphate. Ground Water. 1996;34:1105–14. 10.1111/j.1745-6584.1996.tb02177.x.

