# Supplementary material for: Existing evidence related to soil retention of phosphorus from on-site wastewater treatment systems in boreal and temperate climate zones: a systematic map
Source: Environ Evid. 2023 Apr 3;12:6. doi: 10.1186/s13750-023-00300-7 (PMC11378865; doi:10.1186/s13750-023-00300-7)
Supplement: Supplementary file 2 — Additional file 2. Searching for literature. [file 13750_2023_300_MOESM2_ESM.docx]

*README*

Title: Searching for literature

Description: This additional file describes all the search strings that were used to find peer-reviewed articles and grey literature to include in the systematic map. Two different search strings were used, one for field studies and one for laboratory soil-column studies. Searches were made in bibliographic databases, an academic search engine and websites of relevant organizations.

**Searching for literature**

**Bibliographic database search - Search strings for field studies**

**Database: Scopus**

Database provider: Elsevier

Date of search: September 29, 2021

| **No** | **Search string** | **Number of hits** |
| --- | --- | --- |
|  | **On-site wastewater treatment systems** |  |
| **1** | (TITLE-ABS-KEY(("on site" OR onsite OR "small scale" OR smallscale OR private OR single OR individual OR independent OR residential OR decentral* OR "de-central*") AND ("wastewater treatment" OR "waste water treatment" OR "sewage treatment" OR "wastewater system*" OR "waste water system*" OR "wastewater unit*" OR "waste water unit*" OR "wastewater facilit*" OR "waste water facilit*" OR "wastewater disposal" OR "waste water disposal" OR "sewage system*" OR "sewer* system*" OR "sewage facilit*" OR "sewage disposal" OR "disposal system*" OR "treatment system*" OR "treatment technique*" OR "soil treatment" OR "soil filtration" OR "soil infiltration" OR "land filtration" OR "land infiltration" OR sanitation*))) OR (TITLE-ABS-KEY("septic system*" OR "seepage system*" OR "septic tank*" OR *"septic leach*" OR cesspool* OR cesspit** OR "percolation system*" OR "percolation unit*" OR "percolation facilit*" OR "infiltration system*" OR "infiltration unit*" OR "infiltration facilit*" OR "infiltration site*" OR "infiltration bed*" OR "filter bed*" OR "tile bed*" OR "tile field*" OR "sand bed*" OR "soil bed*" OR "percolation bed*" OR "seepage bed*" OR "soil trench*" OR "percolation trench*" OR "seepage trench*" OR "soil aquifer treatment" OR "soil treatment system*" OR "soil treatment unit*" OR "soil treatment facilit*" OR "land application system*" OR "land treatment system*" OR "land based treatment" OR "land based disposal" OR "disposal to land" OR "disposal field*" OR "leach* field*" OR leachfield* OR "drain* field*" OR drainfield* OR "small wastewater treatment system*" OR "small waste water treatment system*" OR "small wastewater treatment unit*" OR "small waste water treatment unit*" OR "small wastewater treatment facilit*" OR "small waste water treatment facilit*" OR "small sewage treatment system*" OR "small sewage treatment unit*" OR "small sewage treatment facilit*" OR "small wastewater system*" OR "small waste water system*" OR "small wastewater unit*" OR "small waste water unit*" OR "small wastewater facilit*" OR "small waste water facilit*" OR "small wastewater disposal" OR "small waste water disposal" OR "small sewage system*" OR "small sewer* system*" OR "small sewage facilit*" OR "small sewage disposal" OR "domestic wastewater treatment system*" OR "domestic waste water treatment system*" OR "domestic wastewater treatment unit*" OR "domestic waste water treatment unit*" OR "domestic wastewater treatment facilit*" OR "domestic waste water treatment facilit*" OR "domestic sewage treatment system*" OR "domestic sewage treatment unit*" OR "domestic sewage treatment facilit*" OR "domestic wastewater system*" OR "domestic waste water system*" OR "domestic wastewater unit*" OR "domestic waste water unit*" OR "domestic wastewater facilit*" OR "domestic waste water facilit*" OR "domestic wastewater disposal" OR "domestic waste water disposal" OR "domestic sewage system*" OR "domestic sewer* system*" OR "domestic sewage facilit*" OR "domestic sewage disposal" OR OWS OR OWSs OR OWT OR OWTs OR OWTSs)) *OR ((TITLE-ABS-KEY("waste water" OR wastewater OR sewage* OR "domestic effluent*" OR "black water" OR blackwater OR "grey water" OR greywater OR "gray water" OR greywater OR "sanitary water*") AND TITLE-ABS-KEY(irrigat* OR "spray application" OR "land application" OR fertigat* OR sprinkl* OR "slow rate" OR "overland flow") AND TITLE-ABS-KEY(sand* OR soil* OR loam* OR silt OR till OR clay OR peat) AND TITLE-ABS-KEY(attenuat* OR adsorption OR adsorb* OR absorb* OR bind* OR captur* OR fixat* OR immobili* OR precipitat* OR purification OR reduction* OR remov* OR renovat* OR retain* OR retardation OR retention OR sorption OR filtrat* OR infiltrat* OR percolat* OR transport* OR desorption OR desorb* OR mobili* OR leach* OR dissol*) AND TITLE-ABS-KEY(phosphor* OR polyphosphate* OR orthophosphate* OR phosphate* OR *po4*)))* | **49 043** |
|  | **Phosphorus or eutrophication** |  |
| **2** | (TITLE-ABS-KEY(phosphor* OR polyphosphate* OR orthophosphate* OR phosphate* OR *po4* OR eutrophi* OR trophic* OR oligotrophi*)) | **1 830 781** |
|  | **Combination of search strings** |  |
| **3** | 1 AND 2 | **4 450** |
|  | **Limit to language: English, Danish, Norwegian, Swedish** |  |
| **4** | AND (LIMIT-TO(LANGUAGE, “English”) OR LIMIT-TO(LANGUAGE, “Danish”) OR LIMIT-TO(LANGUAGE, “Norwegian”) OR LIMIT-TO( LANGUAGE, “Swedish”)) | **4 127** |

* = An asterisk represents any group of characters, including no character

" " = Citation Marks searches for an exact phrase

TITLE-ABS-KEY = Title or Abstract or Keywords
*Search terms in italic letters are deviations from the systematic map protocol*

**Database: Web of Science Core Collection (1970-)**

Database provider: Clarivate Analytics

Date of search: September 29, 2021

Including: Science Citation Index Expanded (SCI-EXPANDED), Social Sciences Citation Index (SSCI), Arts & Humanities Citation Index (A&HCI), Conference Proceedings Citation Index- Science (CPCI-S), Conference Proceedings Citation Index- Social Science & Humanities (CPCI-SSH) and Emerging Sources Citation Index (ESCI)

| **No** | **Search string** | **Number of hits** |
| --- | --- | --- |
|  | **On-site wastewater treatment systems** |  |
| **1** | TS=("on site" OR onsite OR "small scale" OR smallscale OR private OR single OR individual OR independent OR residential OR decentral* OR "de-central*") AND TS=("wastewater treatment" OR "waste water treatment" OR "sewage treatment" OR "wastewater system*" OR "waste water system*" OR "wastewater unit*" OR "waste water unit*" OR "wastewater facilit*" OR "waste water facilit*" OR "wastewater disposal" OR "waste water disposal" OR "sewage system*" OR "sewer* system*" OR "sewage facilit*" OR "sewage disposal" OR "disposal system*" OR "treatment system*" OR "treatment technique*" OR "soil treatment" OR "soil filtration" OR "soil infiltration" OR "land filtration" OR "land infiltration" OR sanitation*) OR (TS=("septic system*" OR "seepage system*" OR "septic tank*" OR *"septic leach*" OR cesspool* OR cesspit** OR "percolation system*" OR "percolation unit*" OR "percolation facilit*" OR "infiltration system*" OR "infiltration unit*" OR "infiltration facilit*" OR "infiltration site*" OR "infiltration bed*" OR "filter bed*" OR "tile bed*" OR "tile field*" OR "sand bed*" OR "soil bed*" OR "percolation bed*" OR "seepage bed*" OR "soil trench*" OR "percolation trench*" OR "seepage trench*" OR "soil aquifer treatment" OR "soil treatment system*" OR "soil treatment unit*" OR "soil treatment facilit*" OR "land application system*" OR "land treatment system*" OR "land based treatment" OR "land based disposal" OR "disposal to land" OR "disposal field*" OR "leach* field*" OR leachfield* OR "drain* field*" OR drainfield* OR "small wastewater treatment system*" OR "small waste water treatment system*" OR "small wastewater treatment unit*" OR "small waste water treatment unit*" OR "small wastewater treatment facilit*" OR "small waste water treatment facilit*" OR "small sewage treatment system*" OR "small sewage treatment unit*" OR "small sewage treatment facilit*" OR "small wastewater system*" OR "small waste water system*" OR "small wastewater unit*" OR "small waste water unit*" OR "small wastewater facilit*" OR "small waste water facilit*" OR "small wastewater disposal" OR "small waste water disposal" OR "small sewage system*" OR "small sewer* system*" OR "small sewage facilit*" OR "small sewage disposal" OR "domestic wastewater treatment system*" OR "domestic waste water treatment system*" OR "domestic wastewater treatment unit*" OR "domestic waste water treatment unit*" OR "domestic wastewater treatment facilit*" OR "domestic waste water treatment facilit*" OR "domestic sewage treatment system*" OR "domestic sewage treatment unit*" OR "domestic sewage treatment facilit*" OR "domestic wastewater system*" OR "domestic waste water system*" OR "domestic wastewater unit*" OR "domestic waste water unit*" OR "domestic wastewater facilit*" OR "domestic waste water facilit*" OR "domestic wastewater disposal" OR "domestic waste water disposal" OR "domestic sewage system*" OR "domestic sewer* system*" OR "domestic sewage facilit*" OR "domestic sewage disposal" OR OWS OR OWSs OR OWT OR OWTs OR OWTSs)) *OR (TS=("waste water" OR wastewater OR sewage* OR "domestic effluent*" OR "black water" OR blackwater OR "grey water" OR greywater OR "gray water" OR greywater OR "sanitary water*") AND TS=(irrigat* OR "spray application" OR "land application" OR fertigat* OR sprinkl* OR "slow rate" OR "overland flow") AND TS=(sand* OR soil* OR loam* OR silt OR till OR clay OR peat) AND TS=(attenuat* OR adsorption OR adsorb* OR absorb* OR bind* OR captur* OR fixat* OR immobili* OR precipitat* OR purification OR reduction* OR remov* OR renovat* OR retain* OR retardation OR retention OR sorption OR filtrat* OR infiltrat* OR percolat* OR transport* OR desorption OR desorb* OR mobili* OR leach* OR dissol*) AND TS=(phosphor* OR polyphosphate* OR orthophosphate* OR phosphate* OR *po4*))* | **28 624** |
|  | **Phosphorus or eutrophication** |  |
| **2** | TS=(phosphor* OR polyphosphate* OR orthophosphate* OR phosphate* OR *po4* OR eutrophi* OR trophic* OR oligotrophi*) | **1 333 288** |
|  | **Combination of search strings** |  |
| **3** | 1 AND 2 | **3 094** |
|  | **Limit to language: English, Danish, Norwegian, Swedish** |  |
| **4** | Refined by: LANGUAGES: ( ENGLISH OR DANISH OR NORWEGIAN OR SWEDISH ) | **3 054** |

* = An asterisk represents any group of characters, including no character

" " = Citation Marks searches for an exact phrase

TS = Topic Search (search the Title, Abstract, Author Keywords and Keywords Plus within every record)
*Search terms in italic letters are deviations from the systematic map protocol*

**Database: Academic Search Premier**

Database provider: EBSCO

Date of search: December 2, 2019
Search mode: Boolean/Phrase

Expanders: Apply equivalent subjects

| **No** | **Search string** | **Number of hits** |
| --- | --- | --- |
|  | **On-site wastewater treatment systems** |  |
| **1** | ((SU ("on site" OR onsite OR "small scale" OR smallscale OR private OR single OR individual OR independent OR residential OR decentral* OR "de-central*") OR TI ("on site" OR onsite OR "small scale" OR smallscale OR private OR single OR individual OR independent OR residential OR decentral* OR "de-central*") OR AB ("on site" OR onsite OR "small scale" OR smallscale OR private OR single OR individual OR independent OR residential OR decentral* OR "de-central*") OR KW ("on site" OR onsite OR "small scale" OR smallscale OR private OR single OR individual OR independent OR residential OR decentral* OR "de-central*")) AND (SU ("wastewater treatment" OR "waste water treatment" OR "sewage treatment" OR "wastewater system*" OR "waste water system*" OR "wastewater unit*" OR "waste water unit*" OR "wastewater facilit*" OR "waste water facilit*" OR "wastewater disposal" OR "waste water disposal" OR "sewage system*" OR "sewer* system*" OR "sewage facilit*" OR "sewage disposal" OR "disposal system*" OR "treatment system*" OR "treatment technique*" OR "soil treatment" OR "soil filtration" OR "soil infiltration" OR "land filtration" OR "land infiltration" OR sanitation*) OR TI ("wastewater treatment" OR "waste water treatment" OR "sewage treatment" OR "wastewater system*" OR "waste water system*" OR "wastewater unit*" OR "waste water unit*" OR "wastewater facilit*" OR "waste water facilit*" OR "wastewater disposal" OR "waste water disposal" OR "sewage system*" OR "sewer* system*" OR "sewage facilit*" OR "sewage disposal" OR "disposal system*" OR "treatment system*" OR "treatment technique*" OR "soil treatment" OR "soil filtration" OR "soil infiltration" OR "land filtration" OR "land infiltration" OR sanitation*) OR AB ("wastewater treatment" OR "waste water treatment" OR "sewage treatment" OR "wastewater system*" OR "waste water system*" OR "wastewater unit*" OR "waste water unit*" OR "wastewater facilit*" OR "waste water facilit*" OR "wastewater disposal" OR "waste water disposal" OR "sewage system*" OR "sewer* system*" OR "sewage facilit*" OR "sewage disposal" OR "disposal system*" OR "treatment system*" OR "treatment technique*" OR "soil treatment" OR "soil filtration" OR "soil infiltration" OR "land filtration" OR "land infiltration" OR sanitation*) OR KW ("wastewater treatment" OR "waste water treatment" OR "sewage treatment" OR "wastewater system*" OR "waste water system*" OR "wastewater unit*" OR "waste water unit*" OR "wastewater facilit*" OR "waste water facilit*" OR "wastewater disposal" OR "waste water disposal" OR "sewage system*" OR "sewer* system*" OR "sewage facilit*" OR "sewage disposal" OR "disposal system*" OR "treatment system*" OR "treatment technique*" OR "soil treatment" OR "soil filtration" OR "soil infiltration" OR "land filtration" OR "land infiltration" OR sanitation*))) OR (SU ("septic system*" OR "seepage system*" OR "septic tank*" OR "percolation system*" OR "percolation unit*" OR "percolation facilit*" OR "infiltration system*" OR "infiltration unit*" OR "infiltration facilit*" OR "infiltration site*" OR "infiltration bed*" OR "filter bed*" OR "tile bed*" OR "tile field*" OR "sand bed*" OR "soil bed*" OR "percolation bed*" OR "seepage bed*" OR "soil trench*" OR "percolation trench*" OR "seepage trench*" OR "soil aquifer treatment" OR "soil treatment system*" OR "soil treatment unit*" OR "soil treatment facilit*" OR "land application system*" OR "land treatment system*" OR "land based treatment" OR "land based disposal" OR "disposal to land" OR "disposal field*" OR "leach* field*" OR leachfield* OR "drain* field*" OR drainfield* OR "small wastewater treatment system*" OR "small waste water treatment system*" OR "small wastewater treatment unit*" OR "small waste water treatment unit*" OR "small wastewater treatment facilit*" OR "small waste water treatment facilit*" OR "small sewage treatment system*" OR "small sewage treatment unit*" OR "small sewage treatment facilit*" OR "small wastewater system*" OR "small waste water system*" OR "small wastewater unit*" OR "small waste water unit*" OR "small wastewater facilit*" OR "small waste water facilit*" OR "small wastewater disposal" OR "small waste water disposal" OR "small sewage system*" OR "small sewer* system*" OR "small sewage facilit*" OR "small sewage disposal" OR "domestic wastewater treatment system*" OR "domestic waste water treatment system*" OR "domestic wastewater treatment unit*" OR "domestic waste water treatment unit*" OR "domestic wastewater treatment facilit*" OR "domestic waste water treatment facilit*" OR "domestic sewage treatment system*" OR "domestic sewage treatment unit*" OR "domestic sewage treatment facilit*" OR "domestic wastewater system*" OR "domestic waste water system*" OR "domestic wastewater unit*" OR "domestic waste water unit*" OR "domestic wastewater facilit*" OR "domestic waste water facilit*" OR "domestic wastewater disposal" OR "domestic waste water disposal" OR "domestic sewage system*" OR "domestic sewer* system*" OR "domestic sewage facilit*" OR "domestic sewage disposal" OR OWS OR OWSs OR OWT OR OWTs OR OWTSs) OR TI ("septic system*" OR "seepage system*" OR "septic tank*" OR "percolation system*" OR "percolation unit*" OR "percolation facilit*" OR "infiltration system*" OR "infiltration unit*" OR "infiltration facilit*" OR "infiltration site*" OR "infiltration bed*" OR "filter bed*" OR "tile bed*" OR "tile field*" OR "sand bed*" OR "soil bed*" OR "percolation bed*" OR "seepage bed*" OR "soil trench*" OR "percolation trench*" OR "seepage trench*" OR "soil aquifer treatment" OR "soil treatment system*" OR "soil treatment unit*" OR "soil treatment facilit*" OR "land application system*" OR "land treatment system*" OR "land based treatment" OR "land based disposal" OR "disposal to land" OR "disposal field*" OR "leach* field*" OR leachfield* OR "drain* field*" OR drainfield* OR "small wastewater treatment system*" OR "small waste water treatment system*" OR "small wastewater treatment unit*" OR "small waste water treatment unit*" OR "small wastewater treatment facilit*" OR "small waste water treatment facilit*" OR "small sewage treatment system*" OR "small sewage treatment unit*" OR "small sewage treatment facilit*" OR "small wastewater system*" OR "small waste water system*" OR "small wastewater unit*" OR "small waste water unit*" OR "small wastewater facilit*" OR "small waste water facilit*" OR "small wastewater disposal" OR "small waste water disposal" OR "small sewage system*" OR "small sewer* system*" OR "small sewage facilit*" OR "small sewage disposal" OR "domestic wastewater treatment system*" OR "domestic waste water treatment system*" OR "domestic wastewater treatment unit*" OR "domestic waste water treatment unit*" OR "domestic wastewater treatment facilit*" OR "domestic waste water treatment facilit*" OR "domestic sewage treatment system*" OR "domestic sewage treatment unit*" OR "domestic sewage treatment facilit*" OR "domestic wastewater system*" OR "domestic waste water system*" OR "domestic wastewater unit*" OR "domestic waste water unit*" OR "domestic wastewater facilit*" OR "domestic waste water facilit*" OR "domestic wastewater disposal" OR "domestic waste water disposal" OR "domestic sewage system*" OR "domestic sewer* system*" OR "domestic sewage facilit*" OR "domestic sewage disposal" OR OWS OR OWSs OR OWT OR OWTs OR OWTSs) OR AB ("septic system*" OR "seepage system*" OR "septic tank*" OR "percolation system*" OR "percolation unit*" OR "percolation facilit*" OR "infiltration system*" OR "infiltration unit*" OR "infiltration facilit*" OR "infiltration site*" OR "infiltration bed*" OR "filter bed*" OR "tile bed*" OR "tile field*" OR "sand bed*" OR "soil bed*" OR "percolation bed*" OR "seepage bed*" OR "soil trench*" OR "percolation trench*" OR "seepage trench*" OR "soil aquifer treatment" OR "soil treatment system*" OR "soil treatment unit*" OR "soil treatment facilit*" OR "land application system*" OR "land treatment system*" OR "land based treatment" OR "land based disposal" OR "disposal to land" OR "disposal field*" OR "leach* field*" OR leachfield* OR "drain* field*" OR drainfield* OR "small wastewater treatment system*" OR "small waste water treatment system*" OR "small wastewater treatment unit*" OR "small waste water treatment unit*" OR "small wastewater treatment facilit*" OR "small waste water treatment facilit*" OR "small sewage treatment system*" OR "small sewage treatment unit*" OR "small sewage treatment facilit*" OR "small wastewater system*" OR "small waste water system*" OR "small wastewater unit*" OR "small waste water unit*" OR "small wastewater facilit*" OR "small waste water facilit*" OR "small wastewater disposal" OR "small waste water disposal" OR "small sewage system*" OR "small sewer* system*" OR "small sewage facilit*" OR "small sewage disposal" OR "domestic wastewater treatment system*" OR "domestic waste water treatment system*" OR "domestic wastewater treatment unit*" OR "domestic waste water treatment unit*" OR "domestic wastewater treatment facilit*" OR "domestic waste water treatment facilit*" OR "domestic sewage treatment system*" OR "domestic sewage treatment unit*" OR "domestic sewage treatment facilit*" OR "domestic wastewater system*" OR "domestic waste water system*" OR "domestic wastewater unit*" OR "domestic waste water unit*" OR "domestic wastewater facilit*" OR "domestic waste water facilit*" OR "domestic wastewater disposal" OR "domestic waste water disposal" OR "domestic sewage system*" OR "domestic sewer* system*" OR "domestic sewage facilit*" OR "domestic sewage disposal" OR OWS OR OWSs OR OWT OR OWTs OR OWTSs) OR KW ("septic system*" OR "seepage system*" OR "septic tank*" OR "percolation system*" OR "percolation unit*" OR "percolation facilit*" OR "infiltration system*" OR "infiltration unit*" OR "infiltration facilit*" OR "infiltration site*" OR "infiltration bed*" OR "filter bed*" OR "tile bed*" OR "tile field*" OR "sand bed*" OR "soil bed*" OR "percolation bed*" OR "seepage bed*" OR "soil trench*" OR "percolation trench*" OR "seepage trench*" OR "soil aquifer treatment" OR "soil treatment system*" OR "soil treatment unit*" OR "soil treatment facilit*" OR "land application system*" OR "land treatment system*" OR "land based treatment" OR "land based disposal" OR "disposal to land" OR "disposal field*" OR "leach* field*" OR leachfield* OR "drain* field*" OR drainfield* OR "small wastewater treatment system*" OR "small waste water treatment system*" OR "small wastewater treatment unit*" OR "small waste water treatment unit*" OR "small wastewater treatment facilit*" OR "small waste water treatment facilit*" OR "small sewage treatment system*" OR "small sewage treatment unit*" OR "small sewage treatment facilit*" OR "small wastewater system*" OR "small waste water system*" OR "small wastewater unit*" OR "small waste water unit*" OR "small wastewater facilit*" OR "small waste water facilit*" OR "small wastewater disposal" OR "small waste water disposal" OR "small sewage system*" OR "small sewer* system*" OR "small sewage facilit*" OR "small sewage disposal" OR "domestic wastewater treatment system*" OR "domestic waste water treatment system*" OR "domestic wastewater treatment unit*" OR "domestic waste water treatment unit*" OR "domestic wastewater treatment facilit*" OR "domestic waste water treatment facilit*" OR "domestic sewage treatment system*" OR "domestic sewage treatment unit*" OR "domestic sewage treatment facilit*" OR "domestic wastewater system*" OR "domestic waste water system*" OR "domestic wastewater unit*" OR "domestic waste water unit*" OR "domestic wastewater facilit*" OR "domestic waste water facilit*" OR "domestic wastewater disposal" OR "domestic waste water disposal" OR "domestic sewage system*" OR "domestic sewer* system*" OR "domestic sewage facilit*" OR "domestic sewage disposal" OR OWS OR OWSs OR OWT OR OWTs OR OWTSs)) | **10 324** |
|  | **Phosphorus or eutrophication** |  |
| **2** | (SU (phosphor* OR polyphosphate* OR orthophosphate* OR phosphate* OR po4* OR kh2po4 OR eutrophi* OR trophic* OR oligotrophi*) OR TI (phosphor* OR polyphosphate* OR orthophosphate* OR phosphate* OR po4* OR kh2po4 OR eutrophi* OR trophic* OR oligotrophi*) OR AB (phosphor* OR polyphosphate* OR orthophosphate* OR phosphate* OR po4* OR kh2po4 OR eutrophi* OR trophic* OR oligotrophi*) OR KW (phosphor* OR polyphosphate* OR orthophosphate* OR phosphate* OR po4* OR kh2po4 OR eutrophi* OR trophic* OR oligotrophi*)) | **442 729** |
|  | **Combination of search strings** |  |
| **3** | 1 AND 2 | **1 062** |
|  | **Limit to language: English, Danish, Norwegian, Swedish** |  |
| **4** | AND (LA(english OR danish OR norwegian OR swedish)) | **1 042** |

* = An asterisk represents any group of characters, including no character

" " = Citation Marks searches for an exact phrase
SU = Subject Terms
TI = Title
AB = Abstract
KW = Author-Supplied Keywords
LA = Language

**Database: CAB Abstracts (1973-)**

Database provider: Ovid

Date of search: September 30, 2021

| **No** | **Search string** | **Number of hits** |
| --- | --- | --- |
|  | **On-site wastewater treatment systems** |  |
| **1** | (("on site" OR onsite OR "small scale" OR smallscale OR private OR single OR individual OR independent OR residential OR decentral* OR "de-central*") AND ("wastewater treatment" OR "waste water treatment" OR "sewage treatment" OR "wastewater system*" OR "waste water system*" OR "wastewater unit*" OR "waste water unit*" OR "wastewater facilit*" OR "waste water facilit*" OR "wastewater disposal" OR "waste water disposal" OR "sewage system*" OR "sewer* system*" OR "sewage facilit*" OR "sewage disposal" OR "disposal system*" OR "treatment system*" OR "treatment technique*" OR "soil treatment" OR "soil filtration" OR "soil infiltration" OR "land filtration" OR "land infiltration" OR sanitation*)).ti,ab,hw. OR ("septic system*" OR "seepage system*" OR "septic tank*" OR *"septic leach*" OR cesspool* OR cesspit** OR "percolation system*" OR "percolation unit*" OR "percolation facilit*" OR "infiltration system*" OR "infiltration unit*" OR "infiltration facilit*" OR "infiltration site*" OR "infiltration bed*" OR "filter bed*" OR "tile bed*" OR "tile field*" OR "sand bed*" OR "soil bed*" OR "percolation bed*" OR "seepage bed*" OR "soil trench*" OR "percolation trench*" OR "seepage trench*" OR "soil aquifer treatment" OR "soil treatment system*" OR "soil treatment unit*" OR "soil treatment facilit*" OR "land application system*" OR "land treatment system*" OR "land based treatment" OR "land based disposal" OR "disposal to land" OR "disposal field*" OR "leach* field*" OR leachfield* OR "drain* field*" OR drainfield* OR "small wastewater treatment system*" OR "small waste water treatment system*" OR "small wastewater treatment unit*" OR "small waste water treatment unit*" OR "small wastewater treatment facilit*" OR "small waste water treatment facilit*" OR "small sewage treatment system*" OR "small sewage treatment unit*" OR "small sewage treatment facilit*" OR "small wastewater system*" OR "small waste water system*" OR "small wastewater unit*" OR "small waste water unit*" OR "small wastewater facilit*" OR "small waste water facilit*" OR "small wastewater disposal" OR "small waste water disposal" OR "small sewage system*" OR "small sewer* system*" OR "small sewage facilit*" OR "small sewage disposal" OR "domestic wastewater treatment system*" OR "domestic waste water treatment system*" OR "domestic wastewater treatment unit*" OR "domestic waste water treatment unit*" OR "domestic wastewater treatment facilit*" OR "domestic waste water treatment facilit*" OR "domestic sewage treatment system*" OR "domestic sewage treatment unit*" OR "domestic sewage treatment facilit*" OR "domestic wastewater system*" OR "domestic waste water system*" OR "domestic wastewater unit*" OR "domestic waste water unit*" OR "domestic wastewater facilit*" OR "domestic waste water facilit*" OR "domestic wastewater disposal" OR "domestic waste water disposal" OR "domestic sewage system*" OR "domestic sewer* system*" OR "domestic sewage facilit*" OR "domestic sewage disposal" OR OWS OR OWSs OR OWT OR OWTs OR OWTSs).ti,ab,hw. *OR (("waste water" OR wastewater OR sewage* OR "domestic effluent*" OR "black water" OR blackwater OR "grey water" OR greywater OR "gray water" OR greywater OR "sanitary water*") AND (irrigat* OR "spray application" OR "land application" OR fertigat* OR sprinkl* OR "slow rate" OR "overland flow") AND (sand* OR soil* OR loam* OR silt OR till OR clay OR peat) AND (attenuat* OR adsorption OR adsorb* OR absorb* OR bind* OR captur* OR fixat* OR immobili* OR precipitat* OR purification OR reduction* OR remov* OR renovat* OR retain* OR retardation OR retention OR sorption OR filtrat* OR infiltrat* OR percolat* OR transport* OR desorption OR desorb* OR mobili* OR leach* OR dissol*) AND (phosphor* OR polyphosphate* OR orthophosphate* OR phosphate* OR po4* OR kh2po4)).ti,ab,hw.* | **21 940** |
|  | **Phosphorus or eutrophication** |  |
| **2** | (phosphor* OR polyphosphate* OR orthophosphate* OR phosphate* OR po4* OR kh2po4 OR eutrophi* OR trophic* OR oligotrophi*).ti,ab,hw. | **435 993** |
|  | **Combination of search strings** |  |
| **3** | 1 AND 2 | **3 077** |
|  | **Limit to language: English, Danish, Norwegian, Swedish** |  |
| **4** | Limit 3 to (english OR danish OR norwegian OR swedish) | **2 799** |

* = An asterisk represents any group of characters, including no character
" " = Citation Marks searches for an exact phrase
.ti,ab,hw. = Title or Abstract or Heading words
*Search terms in italic letters are deviations from the systematic map protocol*

**Database: Directory of Open Access Journals (DOAJ)**

Database provider: Independent
Date of search: December 6, 2019

DOAJ is a community-curated list of open access journals and aims to be the starting point for information searches for quality, peer reviewed open access material.

| **Search string** (search in all fields) | **Number of hits** (duplicates included) |
| --- | --- |
| “onsite wastewater” AND phosphor* “on site wastewater” AND phosphor* “small scale wastewater” AND phosphor*  “onsite sewage” AND phosphor*  “on site sewage” AND phosphor* “small scale sewage” AND phosphor*  “septic system” AND phosphor*  “septic systems” AND phosphor* “septic tank” AND phosphor* “septic tanks” AND phosphor*  “onsite wastewater” AND eutrophi* “on site wastewater” AND eutrophi* “small scale wastewater” AND eutrophi*  “onsite sewage” AND eutrophi*  “on site sewage” AND eutrophi* “small scale sewage” AND eutrophi*  “septic system” AND eutrophi*  “septic systems” AND eutrophi* “septic tank” AND eutrophi* “septic tanks” AND eutrophi* | **8**  **18**  **17**  **3**  **8**  **7**  **27**  **15**  **22**  **4**  **2**  **11**  **6**  **1**  **10**  **5**  **4**  **6**  **4**  **5** |

* = An asterisk represents any group of characters, including no character
" " = Citation Marks searches for an exact phrase

**Database: DiVA**

Database provider: Swedish universities and research institutions
Date of search: December 10, 2019

DiVA contains research publications and student theses from Swedish universities and research institutions.

| **Language** | **Search string** (search in all fields) | **Number of hits** (duplicates included) |
| --- | --- | --- |
| **English** | “onsite wastewater” AND phosphor* “on site wastewater” AND phosphor* “small scale wastewater” AND phosphor*  “onsite sewage” AND phosphor*  “on site sewage” AND phosphor* “small scale sewage” AND phosphor*  “septic system” AND phosphor*  “septic systems” AND phosphor* “septic tank” AND phosphor* “septic tanks” AND phosphor*  “onsite wastewater” AND eutrophi* “on site wastewater” AND eutrophi* “small scale wastewater” AND eutrophi*  “onsite sewage” AND eutrophi*  “on site sewage” AND eutrophi* “small scale sewage” AND eutrophi*  “septic system” AND eutrophi*  “septic systems” AND eutrophi* “septic tank” AND eutrophi* “septic tanks” AND eutrophi* | **78**  **77**  **44**  **19**  **24**  **12**  **12**  **13**  **11**  **9**  **21**  **20**  **15**  **9**  **16**  **7**  **4**  **5**  **3**  **1** |
| **Swedish** | ”enskilda avlopp” AND fosfor*  ”enskilda avloppssystem” AND fosfor*  ”enskilda avloppsanläggningar” AND fosfor*  ”enskilt avlopp” AND fosfor*  ”enskilt avloppssystem” AND fosfor*  ”enskild avloppsanläggning” AND fosfor*  ”små avlopp” AND fosfor*  ”små avloppssystem” AND fosfor*  ”små avloppsanläggningar” AND fosfor*  ”småskaliga avlopp” AND fosfor*  ”småskaligt avlopp” AND fosfor*  ”småskaliga avloppssystem” AND fosfor*  ”småskaligt avloppssystem” AND fosfor*  ”småskaliga avloppsanläggningar” AND fosfor* ”va-anläggning” AND fosfor* ”va-anläggningar” AND fosfor*  ”enskilda avlopp” AND eutrofiering*  ”enskilda avloppssystem” AND eutrofiering*  ”enskilda avloppsanläggningar” AND eutrofiering*  ”enskilt avlopp” AND eutrofiering*  ”enskilt avloppssystem” AND eutrofiering*  ”enskild avloppsanläggning” AND eutrofiering*  ”små avlopp” AND eutrofiering*  ”små avloppssystem” AND eutrofiering*  ”små avloppsanläggningar” AND eutrofiering*  ”småskaliga avlopp” AND eutrofiering*  ”småskaligt avlopp” AND eutrofiering*  ”småskaliga avloppssystem” AND eutrofiering*  ”småskaligt avloppssystem” AND eutrofiering*  ”småskaliga avloppsanläggningar” AND eutrofiering* ”va-anläggning” AND eutrofiering* ”va-anläggningar” AND eutrofiering*  ”enskilda avlopp” AND övergöd*  ”enskilda avloppssystem” AND övergöd*  ”enskilda avloppsanläggningar” AND övergöd*  ”enskilt avlopp” AND övergöd*  ”enskilt avloppssystem” AND övergöd*  ”enskild avloppsanläggning” AND övergöd*  ”små avlopp” AND övergöd*  ”små avloppssystem” AND övergöd*  ”små avloppsanläggningar” AND övergöd*  ”småskaliga avlopp” AND övergöd*  ”småskaligt avlopp” AND övergöd*  ”småskaliga avloppssystem” AND övergöd*  ”småskaligt avloppssystem” AND övergöd*  ”småskaliga avloppsanläggningar” AND övergöd* ”va-anläggning” AND övergöd* ”va-anläggningar” AND övergöd* | **65**  **8**  **12**  **9**  **2**  **0**  **18**  **5**  **5**  **1**  **0**  **2**  **0**  **1**  **25**  **25**  **5**  **1**  **2**  **0**  **0**  **0**  **2**  **1**  **0**  **0**  **0**  **0**  **0**  **0**  **2**  **0**  **33**  **8**  **7**  **3**  **1**  **0**  **7**  **4**  **5**  **0**  **0**  **0**  **0**  **0**  **16**  **10** |

* = An asterisk represents any group of characters, including no character
" " = Citation Marks searches for an exact phrase

**Database: ProQuest Natural Science Collection**

Database provider: ProQuest

Date of search: September 30, 2021

Including: AGRICOLA; Agricultural Science database; Aquatic Sciences and Fisheries Abstracts; Biological Science database; Biological Science index; Earth, atmosphere & Aquatic Science database; Environmental Science database; Environmental Science index; Meteorological & Geoastrophysical Abstracts

| **No** | **Search string** | **Number of hits** |
| --- | --- | --- |
|  | **On-site wastewater treatment systems** |  |
| **1** | (ti,ab,su("on site" OR onsite OR "small scale" OR smallscale OR private OR single OR individual OR independent OR residential OR decentral* OR "de-central*") AND ti,ab,su("wastewater treatment" OR "waste water treatment" OR "sewage treatment" OR "wastewater system" OR "waste water system" OR "wastewater unit" OR "waste water unit" OR "wastewater facilit*" OR "waste water facilit*" OR "wastewater disposal" OR "waste water disposal" OR "sewage system" OR "sewer* system" OR "sewage facilit*" OR "sewage disposal" OR "disposal system" OR "treatment system" OR "treatment technique" OR "soil treatment" OR "soil filtration" OR "soil infiltration" OR "land filtration" OR "land infiltration" OR sanitation*)) OR ti,ab,su("septic system" OR "seepage system" OR "septic tank" OR *"septic leach*" OR cesspool OR cesspit* OR "percolation system" OR "percolation unit" OR "percolation facilit*" OR "infiltration system" OR "infiltration unit" OR "infiltration facilit*" OR "infiltration site" OR "infiltration bed" OR "filter bed" OR "tile bed" OR "tile field" OR "sand bed" OR "soil bed" OR "percolation bed" OR "seepage bed" OR "soil trench" OR "percolation trench" OR "seepage trench" OR "soil aquifer treatment" OR "soil treatment system" OR "soil treatment unit" OR "soil treatment facilit*" OR "land application system" OR "land treatment system" OR "land based treatment" OR "land based disposal" OR "disposal to land" OR "disposal field" OR "leach* field" OR leachfield OR "drain* field" OR drainfield OR "small wastewater treatment system" OR "small waste water treatment system" OR "small wastewater treatment unit" OR "small waste water treatment unit" OR "small wastewater treatment facilit*" OR "small waste water treatment facilit*" OR "small sewage treatment system" OR "small sewage treatment unit" OR "small sewage treatment facilit*" OR "small wastewater system" OR "small waste water system" OR "small wastewater unit" OR "small waste water unit" OR "small wastewater facilit*" OR "small waste water facilit*" OR "small wastewater disposal" OR "small waste water disposal" OR "small sewage system" OR "small sewer* system" OR "small sewage facilit*" OR "small sewage disposal" OR "domestic wastewater treatment system" OR "domestic waste water treatment system" OR "domestic wastewater treatment unit" OR "domestic waste water treatment unit" OR "domestic wastewater treatment facilit*" OR "domestic waste water treatment facilit*" OR "domestic sewage treatment system" OR "domestic sewage treatment unit" OR "domestic sewage treatment facilit*" OR "domestic wastewater system" OR "domestic waste water system" OR "domestic wastewater unit" OR "domestic waste water unit" OR "domestic wastewater facilit*" OR "domestic waste water facilit*" OR "domestic wastewater disposal" OR "domestic waste water disposal" OR "domestic sewage system" OR "domestic sewer* system" OR "domestic sewage facilit*" OR "domestic sewage disposal" OR OWS OR OWSs OR OWT OR OWTs OR OWTSs) *OR ti,ab,su(("waste water" OR wastewater OR sewage OR "domestic effluent" OR "black water" OR blackwater OR "grey water" OR greywater OR "gray water" OR greywater OR "sanitary water") AND (irrigat* OR "spray application" OR "land application" OR fertigat* OR sprinkl* OR "slow rate" OR "overland flow") AND (sand* OR soil* OR loam* OR silt OR till OR clay OR peat) AND (attenuat* OR adsorption OR adsorb* OR absorb* OR bind* OR captur* OR fixat* OR immobili* OR precipitat* OR purification OR reduction* OR remov* OR renovat* OR retain* OR retardation OR retention OR sorption OR filtrat* OR infiltrat* OR percolat* OR transport* OR desorption OR desorb* OR mobili* OR leach* OR dissol*) AND (phosphor* OR polyphosphate* OR orthophosphate* OR phosphate* OR po4* OR kh2po4))* | **66 094** (duplicates included) |
|  | **Phosphorus or eutrophication** |  |
| **2** | ti,ab,su(phosphor* OR polyphosphate* OR orthophosphate* OR phosphate* OR po4* OR kh2po4 OR eutrophi* OR trophic* OR oligotrophi*) | **1 276 413** (duplicates included) |
|  | **Combination of search strings** |  |
| **3** | 1 AND 2 | **7 114** (duplicates included) |
|  | **Limit to language: English, Danish, Norwegian, Swedish** |  |
| **4** | AND la.exact("ENG" OR "DAN" OR "NOR" OR "SWE") | **4 716** (duplicates removed) |

* = An asterisk represents any group of characters, including no character (Formas ProQuest settings is configured to automatically search for the plural forms of the search terms)
" " = Citation Marks searches for an exact phrase
ti,ab,su = Title or Abstract or All subjects & indexing
la.exact = Language
*Search terms in italic letters are deviations from the systematic map protocol*

**Database: SwePub**
Database provider: National Library of Sweden
Date of search: December 10, 2019

SwePub contains references to articles, conference papers and dissertations published at Swedish universities and authorities.

| **Language** | **Search string** | **Number of hits** |
| --- | --- | --- |
| **English** | ("onsite wastewater" OR "on site wastewater" OR "small scale wastewater" OR "onsite sewage" OR "on site sewage" OR "small scale sewage" OR "septic system*" OR "septic tank*") AND (phosphor* OR phosphate* OR eutrophi* OR trophic* OR oligotrophi*) | **128** |
| **Swedish** | ("enskilda avlopp*" OR "enskilt avlopp*" OR "små* avlopp*" OR "va-anläggning*") AND (fosfor* OR fosfat* OR eutrofiering* OR övergöd*) | **46** |

* = An asterisk represents any group of characters, including no character
" " = Citation Marks searches for an exact phrase

**Bibliographic database search - Search strings for laboratory soil-column studies**

**Database: Scopus**

Database provider: Elsevier

Date of search: September 30, 2021

| **No** | **Search string** | **Number of hits** |
| --- | --- | --- |
|  | **Wastewater, or on-site wastewater treatment systems** |  |
| **1** | (TITLE-ABS-KEY("waste water" OR wastewater OR sewage* OR *"domestic effluent*"* OR "black water" OR blackwater OR "grey water" OR greywater OR "gray water" OR greywater *OR "sanitary water*"* OR "reclaimed water" OR "re-claimed water" OR "recycled water" OR "re-cycled water")) OR (TITLE-ABS-KEY(("on site" OR onsite OR small OR smallscale OR private OR domestic OR single OR individual OR independent OR residential OR decentral* OR "de-central*") AND ("sewer* system*" OR "disposal system*" OR "soil treatment" OR "treatment system*" OR "treatment technique*" OR "soil filtration" OR "soil infiltration" OR "land filtration" OR "land infiltration" OR sanitation*))) OR (TITLE-ABS-KEY("septic system*" OR "seepage system*" OR "septic tank*" OR *"septic leach*" OR cesspool* OR cesspit** OR "percolation system*" OR "percolation unit*" OR "percolation facilit*" OR "infiltration system*" OR "infiltration unit*" OR "infiltration facilit*" OR "infiltration site*" OR "infiltration bed*" OR "filter bed*" OR "tile bed*" OR "tile field*" OR "sand bed*" OR "soil bed*" OR "percolation bed*" OR "seepage bed*" OR "soil trench*" OR "percolation trench*" OR "seepage trench*" OR "soil aquifer treatment" OR "soil treatment system*" OR "soil treatment unit*" OR "soil treatment facilit*" OR "land application system*" OR "land treatment system*" OR "land based treatment" OR "land based disposal" OR "disposal to land" OR "disposal field*" OR "leach* field*" OR leachfield* OR "drain* field*" OR drainfield* OR OWS OR OWSs OR OWT OR OWTs OR OWTSs)) | **413 430** |
|  | **Phosphorus** |  |
| **2** | (TITLE-ABS-KEY(phosphor* OR polyphosphate* OR orthophosphate* OR phosphate* OR *po4*)) | **1 722 749** |
|  | **Flow through soils / soil retention** |  |
| **3** | (TITLE-ABS-KEY((sand* OR soil* OR loam* OR silt OR till OR clay OR peat) AND (attenuat* OR adsorption OR adsorb* OR absorb* OR bind* OR bioretention OR captur* OR fixat* OR immobili* OR precipitat* OR purification OR reduction* OR remov* OR renovat* OR retain* OR retardation OR retention OR sorption OR sorb* OR uptake OR filtrat* OR infiltrat* OR migrat* OR percolat* OR transport* OR desorption OR desorb* OR mobili* OR leach* OR dissol* OR drain*))) | **699 977** |
|  | **Study type: Column studies** |  |
| **4** | (TITLE-ABS-KEY(column* OR lysimeter* OR "percolation experiment*" OR "percolation stud*" OR "continuous test*" OR *"continuous flow test*"* OR "continuous experiment*" OR *"continuous flow experiment*"*)) | **650 559** |
|  | **Combination of search strings** |  |
| **5** | 1 AND 2 AND 3 AND 4 | **498** |
|  | **Limit to language: English, Danish, Norwegian, Swedish** |  |
| **6** | AND (LIMIT-TO(LANGUAGE, “English”) OR LIMIT-TO(LANGUAGE, “Danish”) OR LIMIT-TO(LANGUAGE, “Norwegian”) OR LIMIT-TO( LANGUAGE, “Swedish”)) | **463** |

* = An asterisk represents any group of characters, including no character

" " = Citation Marks searches for an exact phrase

TITLE-ABS-KEY = Title or Abstract or Keywords
*Search terms in italic letters are deviations from the systematic map protocol*

**Database: Web of Science Core Collection (1970-)**

Database provider: Clarivate Analytics

Date of search: September 30, 2021

Including: Science Citation Index Expanded (SCI-EXPANDED), Social Sciences Citation Index (SSCI), Arts & Humanities Citation Index (A&HCI), Conference Proceedings Citation Index- Science (CPCI-S), Conference Proceedings Citation Index- Social Science & Humanities (CPCI-SSH) and Emerging Sources Citation Index (ESCI)

| **No** | **Search string** | **Number of hits** |
| --- | --- | --- |
|  | **Wastewater, or on-site wastewater treatment systems** |  |
| **1** | (TS=("waste water" OR wastewater OR sewage* OR *"domestic effluent*"* OR "black water" OR blackwater OR "grey water" OR greywater OR "gray water" OR greywater OR *"sanitary water*"* OR "reclaimed water" OR "re-claimed water" OR "recycled water" OR "re-cycled water")) OR (TS=("on site" OR onsite OR small OR smallscale OR private OR domestic OR single OR individual OR independent OR residential OR decentral* OR "de-central*") AND TS=("sewer* system*" OR "disposal system*" OR "soil treatment" OR "treatment system*" OR "treatment technique*" OR "soil filtration" OR "soil infiltration" OR "land filtration" OR "land infiltration" OR sanitation*)) OR TS=("septic system*" OR "seepage system*" OR "septic tank*" OR *"septic leach*" OR cesspool* OR cesspit** OR "percolation system*" OR "percolation unit*" OR "percolation facilit*" OR "infiltration system*" OR "infiltration unit*" OR "infiltration facilit*" OR "infiltration site*" OR "infiltration bed*" OR "filter bed*" OR "tile bed*" OR "tile field*" OR "sand bed*" OR "soil bed*" OR "percolation bed*" OR "seepage bed*" OR "soil trench*" OR "percolation trench*" OR "seepage trench*" OR "soil aquifer treatment" OR "soil treatment system*" OR "soil treatment unit*" OR "soil treatment facilit*" OR "land application system*" OR "land treatment system*" OR "land based treatment" OR "land based disposal" OR "disposal to land" OR "disposal field*" OR "leach* field*" OR leachfield* OR "drain* field*" OR drainfield* OR OWS OR OWSs OR OWT OR OWTs OR OWTSs) | **303 000** |
|  | **Phosphorus** |  |
| **2** | TS=(phosphor* OR polyphosphate* OR orthophosphate* OR phosphate* OR *po4*) | **1 238 802** |
|  | **Flow through soils / soil retention** |  |
| **3** | TS=(sand* OR soil* OR loam* OR silt OR till OR clay OR peat) AND TS=(attenuat* OR adsorption OR adsorb* OR absorb* OR bind* OR bioretention OR captur* OR fixat* OR immobili* OR precipitat* OR purification OR reduction* OR remov* OR renovat* OR retain* OR retardation OR retention OR sorption OR sorb* OR uptake OR filtrat* OR infiltrat* OR migrat* OR percolat* OR transport* OR desorption OR desorb* OR mobili* OR leach* OR dissol* OR drain*) | **540 032** |
|  | **Study type: Column studies** |  |
| **4** | TS=(column* OR lysimeter* OR "percolation experiment*" OR "percolation stud*" OR "continuous test*" OR *"continuous flow test*"* OR "continuous experiment*" OR *"continuous flow experiment*")* | **446 132** |
|  | **Combination of search strings** |  |
| **5** | 1 AND 2 AND 3 AND 4 | **463** |
|  | **Limit to language: English, Danish, Norwegian, Swedish** |  |
| **6** | Refined by: LANGUAGES: ( ENGLISH OR DANISH OR NORWEGIAN OR SWEDISH ) | **456** |

* = An asterisk represents any group of characters, including no character

" " = Citation Marks searches for an exact phrase

TS = Topic Search (search the Title, Abstract, Author Keywords and Keywords Plus within every record)
*Search terms in italic letters are deviations from the systematic map protocol*

**Database: Academic Search Premier**

Database provider: EBSCO

Date of search: December 12, 2019

| **No** | **Search string** | **Number of hits** |
| --- | --- | --- |
|  | **Wastewater, or on-site wastewater treatment systems** |  |
| **1** | (SU ("waste water" OR wastewater OR sewage* OR "black water" OR blackwater OR "grey water" OR greywater OR "gray water" OR greywater OR "reclaimed water" OR "re-claimed water" OR "recycled water" OR "re-cycled water") OR TI ("waste water" OR wastewater OR sewage* OR "black water" OR blackwater OR "grey water" OR greywater OR "gray water" OR greywater OR "reclaimed water" OR "re-claimed water" OR "recycled water" OR "re-cycled water") OR AB ("waste water" OR wastewater OR sewage* OR "black water" OR blackwater OR "grey water" OR greywater OR "gray water" OR greywater OR "reclaimed water" OR "re-claimed water" OR "recycled water" OR "re-cycled water") OR KW ("waste water" OR wastewater OR sewage* OR "black water" OR blackwater OR "grey water" OR greywater OR "gray water" OR greywater OR "reclaimed water" OR "re-claimed water" OR "recycled water" OR "re-cycled water")) OR ((SU ("on site" OR onsite OR small OR smallscale OR private OR domestic OR single OR individual OR independent OR residential OR decentral* OR "de-central*") OR TI ("on site" OR onsite OR small OR smallscale OR private OR domestic OR single OR individual OR independent OR residential OR decentral* OR "de-central*") OR AB ("on site" OR onsite OR small OR smallscale OR private OR domestic OR single OR individual OR independent OR residential OR decentral* OR "de-central*") OR KW ("on site" OR onsite OR small OR smallscale OR private OR domestic OR single OR individual OR independent OR residential OR decentral* OR "de-central*")) AND SU ("sewer* system*" OR "disposal system*" OR "soil treatment" OR "treatment system*" OR "treatment technique*" OR "soil filtration" OR "soil infiltration" OR "land filtration" OR "land infiltration" OR sanitation*) OR TI ("sewer* system*" OR "disposal system*" OR "soil treatment" OR "treatment system*" OR "treatment technique*" OR "soil filtration" OR "soil infiltration" OR "land filtration" OR "land infiltration" OR sanitation*) OR AB ("sewer* system*" OR "disposal system*" OR "soil treatment" OR "treatment system*" OR "treatment technique*" OR "soil filtration" OR "soil infiltration" OR "land filtration" OR "land infiltration" OR sanitation*) OR KW ("sewer* system*" OR "disposal system*" OR "soil treatment" OR "treatment system*" OR "treatment technique*" OR "soil filtration" OR "soil infiltration" OR "land filtration" OR "land infiltration" OR sanitation*))) OR (SU ("septic system*" OR "seepage system*" OR "septic tank*" OR "percolation system*" OR "percolation unit*" OR "percolation facilit*" OR "infiltration system*" OR "infiltration unit*" OR "infiltration facilit*" OR "infiltration site*" OR "infiltration bed*" OR "filter bed*" OR "tile bed*" OR "tile field*" OR "sand bed*" OR "soil bed*" OR "percolation bed*" OR "seepage bed*" OR "soil trench*" OR "percolation trench*" OR "seepage trench*" OR "soil aquifer treatment" OR "soil treatment system*" OR "soil treatment unit*" OR "soil treatment facilit*" OR "land application system*" OR "land treatment system*" OR "land based treatment" OR "land based disposal" OR "disposal to land" OR "disposal field*" OR "leach* field*" OR leachfield* OR "drain* field*" OR drainfield* OR OWS OR OWSs OR OWT OR OWTs OR OWTSs) OR TI ("septic system*" OR "seepage system*" OR "septic tank*" OR "percolation system*" OR "percolation unit*" OR "percolation facilit*" OR "infiltration system*" OR "infiltration unit*" OR "infiltration facilit*" OR "infiltration site*" OR "infiltration bed*" OR "filter bed*" OR "tile bed*" OR "tile field*" OR "sand bed*" OR "soil bed*" OR "percolation bed*" OR "seepage bed*" OR "soil trench*" OR "percolation trench*" OR "seepage trench*" OR "soil aquifer treatment" OR "soil treatment system*" OR "soil treatment unit*" OR "soil treatment facilit*" OR "land application system*" OR "land treatment system*" OR "land based treatment" OR "land based disposal" OR "disposal to land" OR "disposal field*" OR "leach* field*" OR leachfield* OR "drain* field*" OR drainfield* OR OWS OR OWSs OR OWT OR OWTs OR OWTSs) OR AB ("septic system*" OR "seepage system*" OR "septic tank*" OR "percolation system*" OR "percolation unit*" OR "percolation facilit*" OR "infiltration system*" OR "infiltration unit*" OR "infiltration facilit*" OR "infiltration site*" OR "infiltration bed*" OR "filter bed*" OR "tile bed*" OR "tile field*" OR "sand bed*" OR "soil bed*" OR "percolation bed*" OR "seepage bed*" OR "soil trench*" OR "percolation trench*" OR "seepage trench*" OR "soil aquifer treatment" OR "soil treatment system*" OR "soil treatment unit*" OR "soil treatment facilit*" OR "land application system*" OR "land treatment system*" OR "land based treatment" OR "land based disposal" OR "disposal to land" OR "disposal field*" OR "leach* field*" OR leachfield* OR "drain* field*" OR drainfield* OR OWS OR OWSs OR OWT OR OWTs OR OWTSs) OR KW ("septic system*" OR "seepage system*" OR "septic tank*" OR "percolation system*" OR "percolation unit*" OR "percolation facilit*" OR "infiltration system*" OR "infiltration unit*" OR "infiltration facilit*" OR "infiltration site*" OR "infiltration bed*" OR "filter bed*" OR "tile bed*" OR "tile field*" OR "sand bed*" OR "soil bed*" OR "percolation bed*" OR "seepage bed*" OR "soil trench*" OR "percolation trench*" OR "seepage trench*" OR "soil aquifer treatment" OR "soil treatment system*" OR "soil treatment unit*" OR "soil treatment facilit*" OR "land application system*" OR "land treatment system*" OR "land based treatment" OR "land based disposal" OR "disposal to land" OR "disposal field*" OR "leach* field*" OR leachfield* OR "drain* field*" OR drainfield* OR OWS OR OWSs OR OWT OR OWTs OR OWTSs)) | **120 816** |
|  | **Phosphorus** |  |
| **2** | (SU (phosphor* OR polyphosphate* OR orthophosphate* OR phosphate* OR po4* OR kh2po4) OR TI (phosphor* OR polyphosphate* OR orthophosphate* OR phosphate* OR po4* OR kh2po4) OR AB (phosphor* OR polyphosphate* OR orthophosphate* OR phosphate* OR po4* OR kh2po4) OR KW (phosphor* OR polyphosphate* OR orthophosphate* OR phosphate* OR po4* OR kh2po4)) | **406 815** |
|  | **Flow through soils / soil retention** |  |
| **3** | (SU (sand* OR soil* OR loam* OR silt OR till OR clay OR peat) OR TI (sand* OR soil* OR loam* OR silt OR till OR clay OR peat) OR AB (sand* OR soil* OR loam* OR silt OR till OR clay OR peat) OR KW (sand* OR soil* OR loam* OR silt OR till OR clay OR peat)) AND (SU (attenuat* OR adsorption OR adsorb* OR absorb* OR bind* OR bioretention OR captur* OR fixat* OR immobili* OR precipitat* OR purification OR reduction* OR remov* OR renovat* OR retain* OR retardation OR retention OR sorption OR sorb* OR uptake OR filtrat* OR infiltrat* OR migrat* OR percolat* OR transport* OR desorption OR desorb* OR mobili* OR leach* OR dissol* OR drain*) OR TI (attenuat* OR adsorption OR adsorb* OR absorb* OR bind* OR bioretention OR captur* OR fixat* OR immobili* OR precipitat* OR purification OR reduction* OR remov* OR renovat* OR retain* OR retardation OR retention OR sorption OR sorb* OR uptake OR filtrat* OR infiltrat* OR migrat* OR percolat* OR transport* OR desorption OR desorb* OR mobili* OR leach* OR dissol* OR drain*) OR AB (attenuat* OR adsorption OR adsorb* OR absorb* OR bind* OR bioretention OR captur* OR fixat* OR immobili* OR precipitat* OR purification OR reduction* OR remov* OR renovat* OR retain* OR retardation OR retention OR sorption OR sorb* OR uptake OR filtrat* OR infiltrat* OR migrat* OR percolat* OR transport* OR desorption OR desorb* OR mobili* OR leach* OR dissol* OR drain*) OR KW (attenuat* OR adsorption OR adsorb* OR absorb* OR bind* OR bioretention OR captur* OR fixat* OR immobili* OR precipitat* OR purification OR reduction* OR remov* OR renovat* OR retain* OR retardation OR retention OR sorption OR sorb* OR uptake OR filtrat* OR infiltrat* OR migrat* OR percolat* OR transport* OR desorption OR desorb* OR mobili* OR leach* OR dissol* OR drain*)) | **185 540** |
|  | **Study type: Column studies** |  |
| **4** | (SU (column* OR lysimeter* OR "percolation experiment*" OR "percolation stud*" OR "continuous test*" OR "continuous experiment*") OR TI (column* OR lysimeter* OR "percolation experiment*" OR "percolation stud*" OR "continuous test*" OR "continuous experiment*") OR AB (column* OR lysimeter* OR "percolation experiment*" OR "percolation stud*" OR "continuous test*" OR "continuous experiment*") OR KW (column* OR lysimeter* OR "percolation experiment*" OR "percolation stud*" OR "continuous test*" OR "continuous experiment*")) | **183 576** |
|  | **Combination of search strings** |  |
| **5** | 1 AND 2 AND 3 AND 4 | **112** |
|  | **Limit to language: English, Danish, Norwegian, Swedish** |  |
| **6** | AND (LA(english OR danish OR norwegian OR swedish)) | **111** |

* = An asterisk represents any group of characters, including no character

" " = Citation Marks searches for an exact phrase
SU = Subject Terms
TI = Title
AB = Abstract
KW = Author-Supplied Keywords
LA = Language

**Database: CAB Abstracts (1973-)**

Database provider: Ovid

Date of search: September 30, 2021

| **No** | **Search string** | **Number of hits** |
| --- | --- | --- |
|  | **Wastewater, or on-site wastewater treatment systems** |  |
| **1** | ("waste water" OR wastewater OR sewage* OR *"domestic effluent*"* OR "black water" OR blackwater OR "grey water" OR greywater OR "gray water" OR greywater OR *"sanitary water*"* OR "reclaimed water" OR "re-claimed water" OR "recycled water" OR "re-cycled water").ti,ab,hw. OR (("on site" OR onsite OR small OR smallscale OR private OR domestic OR single OR individual OR independent OR residential OR decentral* OR "de-central*") AND ("sewer* system*" OR "disposal system*" OR "soil treatment" OR "treatment system*" OR "treatment technique*" OR "soil filtration" OR "soil infiltration" OR "land filtration" OR "land infiltration" OR sanitation*)).ti,ab,hw. OR ("septic system*" OR "seepage system*" OR "septic tank*" OR *"septic leach*" OR cesspool* OR cesspit** OR "percolation system*" OR "percolation unit*" OR "percolation facilit*" OR "infiltration system*" OR "infiltration unit*" OR "infiltration facilit*" OR "infiltration site*" OR "infiltration bed*" OR "filter bed*" OR "tile bed*" OR "tile field*" OR "sand bed*" OR "soil bed*" OR "percolation bed*" OR "seepage bed*" OR "soil trench*" OR "percolation trench*" OR "seepage trench*" OR "soil aquifer treatment" OR "soil treatment system*" OR "soil treatment unit*" OR "soil treatment facilit*" OR "land application system*" OR "land treatment system*" OR "land based treatment" OR "land based disposal" OR "disposal to land" OR "disposal field*" OR "leach* field*" OR leachfield* OR "drain* field*" OR drainfield* OR OWS OR OWSs OR OWT OR OWTs OR OWTSs).ti,ab,hw. | **191 375** |
|  | **Phosphorus** |  |
| **2** | (phosphor* OR polyphosphate* OR orthophosphate* OR phosphate* OR po4* OR kh2po4).ti,ab,hw. | **396 852** |
|  | **Flow through soils / soil retention** |  |
| **3** | ((sand* OR soil* OR loam* OR silt OR till OR clay OR peat) AND (attenuat* OR adsorption OR adsorb* OR absorb* OR bind* OR bioretention OR captur* OR fixat* OR immobili* OR precipitat* OR purification OR reduction* OR remov* OR renovat* OR retain* OR retardation OR retention OR sorption OR sorb* OR uptake OR filtrat* OR infiltrat* OR migrat* OR percolat* OR transport* OR desorption OR desorb* OR mobili* OR leach* OR dissol* OR drain*)).ti,ab,hw. | **448 147** |
|  | **Study type: Column studies** |  |
| **4** | (column* OR lysimeter* OR "percolation experiment*" OR "percolation stud*" OR "continuous test*" OR *"continuous flow test*"* OR "continuous experiment*" OR *"continuous flow experiment*"*).ti,ab,hw. | **108 257** |
|  | **Combination of search strings** |  |
| **5** | 1 AND 2 AND 3 AND 4 | **369** |
|  | **Limit to language: English, Danish, Norwegian, Swedish** |  |
| **6** | Limit 5 to (english OR danish OR norwegian OR swedish) | **338** |

* = An asterisk represents any group of characters, including no character
" " = Citation Marks searches for an exact phrase
.ti,ab,hw. = Title or Abstract or Heading words
*Search terms in italic letters are deviations from the systematic map protocol*

**Database: Directory of Open Access Journals (DOAJ)**

Database provider: Independent
Date of search: December 13, 2019

DOAJ is a community-curated list of open access journals and aims to be the starting point for information searches for quality, peer reviewed open access material.

| **Search string** (search in all fields) | **Number of hits** (duplicates included) |
| --- | --- |
| wastewater AND phosphorus AND column* “waste water” AND phosphorus AND column*  sewage AND phosphorus AND column*  “septic system” AND column*  “septic systems” AND column*  “septic tank” AND column*  “septic tanks” AND column*  wastewater AND phosphorus AND lysimeter* “waste water” AND phosphorus AND lysimeter*  sewage AND phosphorus AND lysimeter*  “septic system” AND lysimeter*  “septic systems” AND lysimeter*  “septic tank” AND lysimeter*  “septic tanks” AND lysimeter* | **18**  **5**  **9**  **4**  **3**  **1**  **1**  **3**  **1**  **1**  **0**  **2**  **2**  **0** |

* = An asterisk represents any group of characters, including no character
" " = Citation Marks searches for an exact phrase

**Database: DiVA**

Database provider: Swedish universities and research institutions
Date of search: December 13, 2019

DiVA contains research publications and student theses from Swedish universities and research institutions.

| **Language** | **Search string** (search in all fields) | **Number of hits** (duplicates included) |
| --- | --- | --- |
| **English** | wastewater AND phosphorus AND column* “waste water” AND phosphorus AND column*  sewage AND phosphorus AND column*  “septic system” AND column*  “septic systems” AND column*  “septic tank” AND column*  “septic tanks” AND column*  wastewater AND phosphorus AND lysimeter* “waste water” AND phosphorus AND lysimeter*  sewage AND phosphorus AND lysimeter*  “septic system” AND lysimeter*  “septic systems” AND lysimeter*  “septic tank” AND lysimeter*  “septic tanks” AND lysimeter* | **55**  **10**  **13**  **7**  **6**  **6**  **5**  **2**  **0**  **0**  **0**  **0**  **0**  **0** |
| **Swedish** | avlopp* AND kolonn*  avlopp* AND jordkolonn*  avlopp* AND lysimeter* | **15**  **1**  **0** |

* = An asterisk represents any group of characters, including no character
" " = Citation Marks searches for an exact phrase

**Database: ProQuest Natural Science Collection**

Database provider: ProQuest

Date of search: September 30, 2021

Including: AGRICOLA; Agricultural Science database; Aquatic Sciences and Fisheries Abstracts; Biological Science database; Biological Science index; Earth, atmosphere & Aquatic Science database; Environmental Science database; Environmental Science index; Meteorological & Geoastrophysical Abstracts

| **No** | **Search string** | **Number of hits** |
| --- | --- | --- |
|  | **Wastewater, or on-site wastewater treatment systems** |  |
| **1** | ti,ab,su("waste water" OR wastewater OR sewage* OR *"domestic effluent*"* OR "black water" OR blackwater OR "grey water" OR greywater OR "gray water" OR greywater OR *"sanitary water*"* OR "reclaimed water" OR "re-claimed water" OR "recycled water" OR "re-cycled water") OR (ti,ab,su("on site" OR onsite OR small OR smallscale OR private OR domestic OR single OR individual OR independent OR residential OR decentral* OR "de-central*") AND ti,ab,su("sewer* system" OR "disposal system" OR "soil treatment" OR "treatment system" OR "treatment technique" OR "soil filtration" OR "soil infiltration" OR "land filtration" OR "land infiltration" OR sanitation*)) OR ti,ab,su("septic system" OR "seepage system" OR "septic tank" OR *"septic leach*" OR cesspool OR cesspit* OR "percolation system" OR "percolation unit" OR "percolation facilit*" OR "infiltration system" OR "infiltration unit" OR "infiltration facilit*" OR "infiltration site" OR "infiltration bed" OR "filter bed" OR "tile bed" OR "tile field" OR "sand bed" OR "soil bed" OR "percolation bed" OR "seepage bed" OR "soil trench" OR "percolation trench" OR "seepage trench" OR "soil aquifer treatment" OR "soil treatment system" OR "soil treatment unit" OR "soil treatment facilit*" OR "land application system" OR "land treatment system" OR "land based treatment" OR "land based disposal" OR "disposal to land" OR "disposal field" OR "leach* field" OR leachfield OR "drain* field" OR drainfield OR OWS OR OWSs OR OWT OR OWTs OR OWTSs) | **690 616** (duplicates included) |
|  | **Phosphorus** |  |
| **2** | ti,ab,su(phosphor* OR polyphosphate* OR orthophosphate* OR phosphate* OR po4* OR kh2po4) | **1 107 257** (duplicates included) |
|  | **Flow through soils / soil retention** |  |
| **3** | (ti,ab,su(sand* OR soil* OR loam* OR silt OR till OR clay OR peat) AND ti,ab,su(attenuat* OR adsorption OR adsorb* OR absorb* OR bind* OR bioretention OR captur* OR fixat* OR immobili* OR precipitat* OR purification OR reduction* OR remov* OR renovat* OR retain* OR retardation OR retention OR sorption OR sorb* OR uptake OR filtrat* OR infiltrat* OR migrat* OR percolat* OR transport* OR desorption OR desorb* OR mobili* OR leach* OR dissol* OR drain*)) | **852 678** (duplicates included) |
|  | **Study type: Column studies** |  |
| **4** | ti,ab,su(column* OR lysimeter* OR "percolation experiment*" OR "percolation stud*" OR "continuous test*" OR *"continuous flow test*"* OR "continuous experiment*" OR *"continuous flow experiment*"*) | **518 018** (duplicates included) |
|  | **Combination of search strings** |  |
| **5** | 1 AND 2 AND 3 AND 4 | **1 015** (duplicates included) |
|  | **Limit to language: English, Danish, Norwegian, Swedish** |  |
| **6** | AND la.exact("ENG" OR "DAN" OR "NOR" OR "SWE") | **614** (duplicates removed) |

* = An asterisk represents any group of characters, including no character (Formas ProQuest settings is configured to automatically search for the plural forms of the search terms)
" " = Citation Marks searches for an exact phrase
ti,ab,su = Title or Abstract or All subjects & indexing
la.exact = Language
*Search terms in italic letters are deviations from the systematic map protocol*

**Database: SwePub**

Database provider: National Library of Sweden

Date of search: December 13, 2019

SwePub contains references to articles, conference papers and dissertations published at Swedish universities and authorities.

| **Language** | **Search string** | **Number of hits** |
| --- | --- | --- |
| **English** | (wastewater OR “waste water” OR sewage” OR “septic system*” OR “septic tank*”) AND (column* OR lysimeter* OR “percolation experiment*” OR “percolation stud*”) | **195** |
| **Swedish** | (avloppsvatten OR ”enskilda avlopp*” OR ”enskilt avlopp*” OR ”små* avlopp*” OR ”va-anläggning*”) AND (kolonn* OR jordkolonn* OR lysimeter*) | **3** |

* = An asterisk represents any group of characters, including no character
" " = Citation Marks searches for an exact phrase

**Search engine - Search strings for field studies**

**Search engine: Google Scholar**

Date of search: December 13, 2019
The first 200 results for every search were exported from Google Scholar using Publish or Perish version 6 software: Harzing, A.W. (2007) Publish or Perish, available from <https://harzing.com/resources/publish-or-perish>

| **Language** | **Search string** | **Number of hits** |
| --- | --- | --- |
| **English** | **Phosphorus search** Any of the words: “on site wastewater” “small scale wastewater” “on site sewage” “small scale sewage” “septic system” “septic tank” All of the words: phosphorus  **Eutrophication search** Any of the words: “on site wastewater” “small scale wastewater” “on site sewage” “small scale sewage” “septic system” “septic tank” All of the words: eutrophication | **200** (generated more hits, but we only imported the first 200)  **200** (generated more hits, but we only imported the first 200) |
| **Swedish** | **Fosfor sökning (phosphorus search)** Any of the words: ”enskilda avlopp” ”enskilt avlopp” ”små avlopp” ”va-anläggning” ”va-anläggningar” ”enskilda avloppssystem” ”enskilt avloppssystem” ”enskild avloppsanläggning” ”enskilda avloppsanläggningar” ”små avloppssystem” ”små avloppsanläggningar” ”småskaliga avlopp” ”småskaligt avlopp” ”småskaliga avloppssystem” ”småskaligt avloppssystem” ”småskaliga avloppsanläggningar”  All of the words: fosfor  **Eutrofiering sökning (eutrophication search)** Any of the words: ”enskilda avlopp” ”enskilt avlopp” ”små avlopp” ”va-anläggning” ”va-anläggningar” ”enskilda avloppssystem” ”enskilt avloppssystem” ”enskild avloppsanläggning” ”enskilda avloppsanläggningar” ”små avloppssystem” ”små avloppsanläggningar” ”småskaliga avlopp” ”småskaligt avlopp” ”småskaliga avloppssystem” ”småskaligt avloppssystem” ”småskaliga avloppsanläggningar”  All of the words: eutrofiering  **Övergödning sökning (eutrophication search)** Any of the words: ”enskilda avlopp” ”enskilt avlopp” ”små avlopp” ”va-anläggning” ”va-anläggningar” ”enskilda avloppssystem” ”enskilt avloppssystem” ”enskild avloppsanläggning” ”enskilda avloppsanläggningar” ”små avloppssystem” ”små avloppsanläggningar” ”småskaliga avlopp” ”småskaligt avlopp” ”småskaliga avloppssystem” ”småskaligt avloppssystem” ”småskaliga avloppsanläggningar”  All of the words: övergödning | **200** (generated more hits, but we only imported the first 200)  **200** (generated more hits, but we only imported the first 200)  **200** (generated more hits, but we only imported the first 200) |

" " = Citation Marks searches for an exact phrase

**Search engine - Search strings for laboratory soil-column studies**

**Search engine: Google Scholar**

Date of search: December 13, 2019
The first 200 results for every search were exported from Google Scholar using Publish or Perish version 6 software: Harzing, A.W. (2007) Publish or Perish, available from <https://harzing.com/resources/publish-or-perish>

| **Language** | **Search string** | **Number of hits** |
| --- | --- | --- |
| **English** | **Laboratory columns search** Any of the words: “on site wastewater” “small scale wastewater” “on site sewage” “small scale sewage” “septic system” “septic tank” All of the words: phosphorus columns laboratory  **Column experiments search** Any of the words: “on site wastewater” “small scale wastewater” “on site sewage” “small scale sewage” “septic system” “septic tank” All of the words: phosphorus columns experiment | **200** (generated more hits, but we only imported the first 200)  **200** (generated more hits, but we only imported the first 200) |
| **Swedish** | **Sökning på kolonner (column search)** Any of the words: avloppsvatten ”enskilda avlopp” ”enskilt avlopp” ”små avlopp” ”va-anläggning” ”va-anläggningar” ”enskilda avloppssystem” ”enskilt avloppssystem” ”enskild avloppsanläggning” ”enskilda avloppsanläggningar” ”små avloppssystem” ”små avloppsanläggningar” ”småskaliga avlopp” ”småskaligt avlopp” ”småskaliga avloppssystem” ”småskaligt avloppssystem” ”småskaliga avloppsanläggningar”  All of the words: kolonner  **Sökning på jordkolonner (soil-column search)** Any of the words: avloppsvatten ”enskilda avlopp” ”enskilt avlopp” ”små avlopp” ”va-anläggning” ”va-anläggningar” ”enskilda avloppssystem” ”enskilt avloppssystem” ”enskild avloppsanläggning” ”enskilda avloppsanläggningar” ”små avloppssystem” ”små avloppsanläggningar” ”småskaliga avlopp” ”småskaligt avlopp” ”småskaliga avloppssystem” ”småskaligt avloppssystem” ”småskaliga avloppsanläggningar”  All of the words: jordkolonner | **200** (generated more hits, but we only imported the first 200)  **4** |

“ “ = Citation Marks searches for an exact phrase

**Websites of relevant organizations**

The search languages used on these websites are English or Swedish and sometimes both, depending on the language of the website. Only documents written in English or Swedish was considered to be relevant, due to the language skills of the person performing the search.

| **Website** | **Date** | **Search string** | **Number of potentially relevant documents** |
| --- | --- | --- | --- |
| EEA (European Environment Agency) <https://www.eea.europa.eu> | July 30, 2020 | **Used the search box on the publications page**  wastewater “waste water” sewage septic  phosphorus | **1 0 0 0**  **1** |
| DCE (Danish Centre for Environment and Energy) <http://dce.au.dk> | July 30, 2020 | **Used the regular search box for the entire website** "on site" AND wastewater "on site" AND “waste water” onsite AND wastewater  onsite AND "waste water"  "on site" AND sewage onsite AND sewage small AND wastewater AND phosphorus  small AND "waste water" AND phosphorus sewage AND phosphorus septic  wastewater AND phosphorus AND column* "waste water" AND phosphorus AND column* | **0 0 0 0 0 6**  **0 1 1**  **0**  **0**  **0** |
| Miljøstyrelsen (Danish Environmental Protection Agency) <https://mst.dk> | July 30, 2020 | **Used the regular search box for the entire website** "on site" AND wastewater AND phosphorus "on site" AND “waste water” AND phosphorus onsite AND wastewater AND phosphorus  onsite AND "waste water" AND phosphorus  small AND wastewater AND phosphorus  small AND "waste water" AND phosphorus sewage AND phosphorus septic  wastewater AND phosphorus AND column* "waste water" AND phosphorus AND column* | **0 2 0 0**  **0 0**  **0 0**  **0**  **0** |
| Luke (Natural Resources Institute Finland) <https://www.luke.fi> | July 30, 2020 | **Used Advanced search in their Jukuri publication service https://jukuri.luke.fi/discover?query=&scope=** "on site" AND wastewater AND phosphorus "on site" AND “waste water” AND phosphorus onsite AND wastewater AND phosphorus  onsite AND "waste water" AND phosphorus small AND wastewater AND phosphorus  small AND "waste water" AND phosphorus "on site" AND sewage AND phosphorus onsite AND sewage AND phosphorus small AND sewage AND phosphorus septic AND phosphorus wastewater AND phosphorus AND column* "waste water" AND phosphorus AND column* sewage AND phosphorus AND column*  enskil* AND avlopp*  små* AND avlopp*  va-anläggning*  septiktank* septitank* markbädd* markretention* markrening* | **0 1 0**  **0 4 0**  **0 0 0 0 0 0 0**  **0**  **0**  **0**  **0**  **0**  **0**  **0**  **0** |
| SYKE (Finnish Environment Institute)  https://www.syke.fi | August 19, 2020 | **Used simple search in their publications archive**  **https://helda.helsinki.fi/handle/10138/29865?locale-attribute=en**  "on site" AND wastewater AND phosphorus "on site" AND “waste water” AND phosphorus onsite AND wastewater AND phosphorus  onsite AND "waste water" AND phosphorus small AND wastewater AND phosphorus small AND "waste water" AND phosphorus "on site" AND sewage AND phosphorus onsite AND sewage AND phosphorus small AND sewage AND phosphorus septic AND phosphorus wastewater AND phosphorus AND column* "waste water" AND phosphorus AND column*  sewage AND phosphorus AND column*  "enskilda avlopp"  "enskilda avloppssystem"  "enskilda avloppsanläggningar"  "enskilt avlopp"  "enskilt avloppssystem"  "enskild avloppsanläggning"  "små avlopp"  "små avloppssystem"  "små avloppsanläggningar"  "småskaliga avlopp"  "småskaligt avlopp"  "småskaliga avloppssystem"  "småskaligt avloppssystem"  "småskaliga avloppsanläggningar"  "va-anläggning"  "va-anläggningar"  septiktank* septitank* markbädd* markretention* markrening*  fosfor | **9**  **1**  **0**  **0**  **3**  **0**  **1**  **0**  **1**  **0**  **0**  **0**  **0**  **1**  **1**  **0**  **0**  **0**  **0**  **1**  **0**  **0**  **0**  **0**  **0**  **0**  **0**  **0**  **0**  **0**  **0**  **0**  **0**  **0**  **0** |
| Umweltbundesamt (German Environment Agency)  https://www.umweltbundesamt.de | August 19, 2020 | **Used the search box on the publications page**  wastewater “waste water” sewage septic  phosphorus | **0**  **0**  **0**  **0**  **0** |
| PIA (Development and assessment institute in waste water technology at RWTH Aachen University, Germany)  https://www.pia-gmbh.com | August 20, 2020 | **Used the regular search box for the entire website**  wastewater “waste water” sewage septic  phosphorus | **0**  **0**  **0**  **0**  **0** |
| PIA (Development and assessment institute in waste water technology at RWTH Aachen University, Germany)  http://www.pia.rwth-aachen.de | August 20, 2020 | **No search box on the website** Browsed through the publication list on the publications page  http://www.pia.rwth-aachen.de/index.php/en/publications | **0** |
| EPA Ireland (Environmental Protection Agency, Ireland)  http://epa.ie | August 20, 2020 | **Used the search box on the publications page**  "on site" AND wastewater "on site" AND “waste water” onsite AND wastewater  onsite AND "waste water" "on site" AND sewage onsite AND sewage wastewater AND phosphorus  "waste water" AND phosphorus sewage AND phosphorus septic AND phosphorus | **4**  **0**  **0**  **0**  **0**  **0**  **2**  **0**  **0**  **0** |
| STOWA (Foundation for Applied Water Research, in the Netherlands)  https://www.stowa.nl | August 21, 2020 | **Used the search box on the publications page**  wastewater “waste water” sewage septic  phosphorus | **0**  **0**  **0**  **0**  **0** |
| Wageningen Environmental Research (Research Institute in the Netherlands)  https://www.wur.nl/en/Research-Results/Research-Institutes/Environmental-Research.htm | August 21, 2020 | **Used the search box on the publications page**  **Limit to Content type: Research Output**  "on site" AND wastewater AND phosphorus  "on site" AND “waste water” AND phosphorus  onsite AND wastewater AND phosphorus  onsite AND "waste water" AND phosphorus  small AND wastewater AND phosphorus  small AND "waste water" AND phosphorus  "on site" AND sewage AND phosphorus  onsite AND sewage AND phosphorus  small AND sewage AND phosphorus  septic AND phosphorus  wastewater AND phosphorus AND column*  "waste water" AND phosphorus AND column*  sewage AND phosphorus AND column* | **0**  **0**  **0**  **0**  **0**  **4**  **0**  **0**  **2**  **0**  **0**  **1**  **0** |
| Miljødirektoratet (Norwegian Environment Agency)  https://www.miljodirektoratet.no | October 8, 2020 | **Used the regular search box for the entire website**  wastewater “waste water” sewage septic  phosphorus | **2**  **3**  **0**  **0**  **0** |
| NIBIO (Norwegian Institute of Bioeconomy Research)  https://www.nibio.no | October 8, 2020 | **Used the search box on the publications page**  "on site" AND wastewater AND phosphorus  "on site" AND “waste water” AND phosphorus  onsite AND wastewater AND phosphorus  onsite AND "waste water" AND phosphorus  small AND wastewater AND phosphorus  small AND "waste water" AND phosphorus  "on site" AND sewage AND phosphorus  onsite AND sewage AND phosphorus  small AND sewage AND phosphorus  septic AND phosphorus  wastewater AND phosphorus AND column*  "waste water" AND phosphorus AND column*  sewage AND phosphorus AND column* | **3**  **0**  **0**  **0**  **1**  **0**  **0**  **0**  **0**  **0**  **1**  **0**  **0** |
| NIVA (Norwegian Institute for Water Research)  https://www.niva.no | October 8, 2020 | **Used the regular search box for the entire website**  wastewater “waste water” sewage septic  phosphorus | **0**  **0**  **1**  **0**  **0** |
| NTNU (Norwegian University of Science and Technology)  https://www.ntnu.no | October 8, 2020 | **Used the search box on their publication site NTNU Open**  **https://ntnuopen.ntnu.no/**  **Sort option: Relevance; Results per page: 10**  **Stopped screening when five pages in a row didn't contain any relevant documents**  onsite AND wastewater AND phosphorus  small AND wastewater AND phosphorus  onsite AND sewage AND phosphorus  small AND sewage AND phosphorus  septic AND phosphorus  wastewater AND phosphorus AND column*  sewage AND phosphorus AND column* | **1**  **2**  **0**  **0**  **0**  **0**  **0** |
| Tidsskriftet VANN (Water, a Norwegian journal)  https://vannforeningen.no/tidsskriftet-vann | November 10, 2020 | **Used the search box for the article archive**  **https://vannforeningen.no/dokumentarkiv/**  "on site" wastewater phosphorus  "on site" “waste water” phosphorus  onsite wastewater phosphorus  onsite "waste water" phosphorus  small wastewater phosphorus  small "waste water" phosphorus  "on site" sewage phosphorus  onsite sewage phosphorus  small sewage phosphorus  septic phosphorus  wastewater phosphorus column*  "waste water" phosphorus column*  sewage phosphorus column* | **3**  **0**  **0**  **0**  **1**  **0**  **0**  **0**  **0**  **0**  **0**  **0**  **0** |
| Havs- och vattenmyndigheten (Swedish Agency for Marine and Water Management)  https://www.havochvatten.se | November 10, 2020 | **Used the regular search box on the publications page**  enskilda avlopp  enskilda avloppssystem  enskilda avloppsanläggningar  enskilt avlopp  enskilt avloppssystem  enskild avloppsanläggning  små avlopp  små avloppssystem  små avloppsanläggningar  småskaliga avlopp  småskaligt avlopp  småskaliga avloppssystem  småskaligt avloppssystem  småskaliga avloppsanläggningar  va-anläggning  va-anläggningar  septiktank  septiktankar septitank  septitankar markbädd  markbäddar markretention markrening  fosfor | **8**  **0**  **0**  **0**  **0**  **0**  **0**  **0**  **0**  **0**  **0**  **0**  **0**  **0**  **0**  **0**  **0**  **0**  **0**  **0**  **0**  **0**  **0**  **0**  **3** |
| IVL (Swedish Environmental Research Institute)  https://www.ivl.se | November 10, 2020 | **Used the search box on the publications page**  on site AND wastewater on site AND waste water  onsite AND wastewater  onsite AND waste water on site AND sewage onsite AND sewage wastewater AND phosphorus  waste water AND phosphorus sewage AND phosphorus septic  enskil* AND avlopp*  små* AND avlopp*  va-anläggning*  septiktank* septitank* markbädd* markretention* markrening*  fosfor* | **0**  **0**  **1**  **0**  **0**  **0**  **0**  **0**  **0**  **0**  **6**  **1**  **0**  **0**  **0**  **0**  **0**  **0**  **10** |
| Länsstyrelsen Blekinge (County Administrative Board of Blekinge, Sweden) https://www.lansstyrelsen.se/blekinge | July 29, 2020 | **Used the regular search box for the entire website**  enskil* AND avlopp*  små* AND avlopp*  va-anläggning*  septiktank* septitank* markbädd* markretention* markrening*  fosfor* | **1**  **0**  **0**  **0**  **0**  **0**  **0**  **0**  **1** |
| Länsstyrelsen Dalarna (County Administrative Board of Dalarna, Sweden) https://www.lansstyrelsen.se/dalarna | July 29, 2020 | **Used the regular search box for the entire website**  enskil* AND avlopp*  små* AND avlopp*  va-anläggning*  septiktank* septitank* markbädd* markretention* markrening*  fosfor* | **0**  **0**  **0**  **0**  **0**  **0**  **0**  **0**  **3** |
| Länsstyrelsen Gotland (County Administrative Board of Gotland, Sweden) https://www.lansstyrelsen.se/gotland | July 29, 2020 | **Used the regular search box for the entire website**  enskil* AND avlopp*  små* AND avlopp*  va-anläggning*  septiktank* septitank* markbädd* markretention* markrening*  fosfor* | **1**  **0**  **0**  **0**  **0**  **0**  **0**  **0**  **2** |
| Länsstyrelsen Gävleborg (County Administrative Board of Gävleborg, Sweden) https://www.lansstyrelsen.se/gavleborg | July 29, 2020 | **Used the regular search box for the entire website**  enskil* AND avlopp*  små* AND avlopp*  va-anläggning*  septiktank* septitank* markbädd* markretention* markrening*  fosfor* | **0**  **0**  **0**  **0**  **0**  **0**  **0**  **0**  **0** |
| Länsstyrelsen Halland (County Administrative Board of Halland, Sweden) https://www.lansstyrelsen.se/halland | July 29, 2020 | **Used the regular search box for the entire website**  enskil* AND avlopp*  små* AND avlopp*  va-anläggning*  septiktank* septitank* markbädd* markretention* markrening*  fosfor* | **0**  **0**  **0**  **0**  **0**  **0**  **0**  **0**  **0** |
| Länsstyrelsen Jämtland (County Administrative Board of Jämtland, Sweden) https://www.lansstyrelsen.se/jamtland | July 29, 2020 | **Used the regular search box for the entire website**  enskil* AND avlopp*  små* AND avlopp*  va-anläggning*  septiktank* septitank* markbädd* markretention* markrening*  fosfor* | **0**  **0**  **0**  **0**  **0**  **0**  **0**  **0**  **0** |
| Länsstyrelsen Jönköping (County Administrative Board of Jönköping, Sweden) https://www.lansstyrelsen.se/jonkoping | July 29, 2020 | **Used the regular search box for the entire website**  enskil* AND avlopp*  små* AND avlopp*  va-anläggning*  septiktank* septitank* markbädd* markretention* markrening*  fosfor* | **3**  **0**  **0**  **0**  **0**  **0**  **0**  **0**  **1** |
| Länsstyrelsen Kalmar (County Administrative Board of Kalmar, Sweden) https://www.lansstyrelsen.se/kalmar | July 29, 2020 | **Used the regular search box for the entire website**  enskil* AND avlopp*  små* AND avlopp*  va-anläggning*  septiktank* septitank* markbädd* markretention* markrening*  fosfor* | **0**  **0**  **0**  **0**  **0**  **0**  **0**  **0**  **0** |
| Länsstyrelsen Kronoberg (County Administrative Board of Kronoberg, Sweden) https://www.lansstyrelsen.se/kronoberg | July 29, 2020 | **Used the regular search box for the entire website**  enskil* AND avlopp*  små* AND avlopp*  va-anläggning*  septiktank* septitank* markbädd* markretention* markrening*  fosfor* | **0**  **0**  **0**  **0**  **0**  **0**  **0**  **0**  **0** |
| Länsstyrelsen Norrbotten (County Administrative Board of Norrbotten, Sweden) https://www.lansstyrelsen.se/norrbotten | July 29, 2020 | **Used the regular search box for the entire website**  enskil* AND avlopp*  små* AND avlopp*  va-anläggning*  septiktank* septitank* markbädd* markretention* markrening*  fosfor* | **0**  **0**  **0**  **0**  **0**  **0**  **0**  **0**  **0** |
| Länsstyrelsen Skåne (County Administrative Board of Skåne, Sweden) https://www.lansstyrelsen.se/skane | July 29, 2020 | **Used the regular search box for the entire website**  enskil* AND avlopp*  små* AND avlopp*  va-anläggning*  septiktank* septitank* markbädd* markretention* markrening*  fosfor* | **0**  **0**  **0**  **0**  **0**  **0**  **0**  **0**  **0** |
| Länsstyrelsen Stockholm (County Administrative Board of Stockholm, Sweden) https://www.lansstyrelsen.se/stockholm | July 29, 2020 | **Used the regular search box for the entire website**  enskil* AND avlopp*  små* AND avlopp*  va-anläggning*  septiktank* septitank* markbädd*  markretention* markrening*  fosfor* | **3**  **0**  **0**  **0**  **0**  **0**  **0**  **0**  **1** |
| Länsstyrelsen Södermanland (County Administrative Board of Södermanland, Sweden) <https://www.lansstyrelsen.se/sodermanland> | July 29, 2020 | **Used the regular search box for the entire website**  enskil* AND avlopp*  små* AND avlopp*  va-anläggning*  septiktank* septitank* markbädd* markretention* markrening*  fosfor* | **0**  **0**  **0**  **0**  **0**  **0**  **0**  **0**  **0** |
| Länsstyrelsen Uppsala (County Administrative Board of Uppsala, Sweden) https://www.lansstyrelsen.se/uppsala | July 29, 2020 | **Used the regular search box for the entire website**  enskil* AND avlopp*  små* AND avlopp*  va-anläggning*  septiktank* septitank* markbädd* markretention* markrening*  fosfor* | **0**  **0**  **0**  **0**  **0**  **0**  **0**  **0**  **1** |
| Länsstyrelsen Värmland (County Administrative Board of Värmland, Sweden) https://www.lansstyrelsen.se/varmland | July 29, 2020 | **Used the regular search box for the entire website**  enskil* AND avlopp*  små* AND avlopp*  va-anläggning*  septiktank* septitank* markbädd* markretention* markrening*  fosfor* | **0**  **0**  **0**  **0**  **0**  **0**  **0**  **0**  **0** |
| Länsstyrelsen Västerbotten (County Administrative Board of Västerbotten, Sweden) https://www.lansstyrelsen.se/vasterbotten | July 29, 2020 | **Used the regular search box for the entire website**  enskil* AND avlopp*  små* AND avlopp*  va-anläggning*  septiktank* septitank* markbädd* markretention* markrening*  fosfor* | **0**  **0**  **0**  **0**  **0**  **0**  **0**  **0**  **0** |
| Länsstyrelsen Västernorrland (County Administrative Board of Västernorrland, Sweden) https://www.lansstyrelsen.se/vasternorrland | July 29, 2020 | **Used the regular search box for the entire website**  enskil* AND avlopp*  små* AND avlopp*  va-anläggning*  septiktank* septitank* markbädd* markretention* markrening*  fosfor* | **0**  **0**  **0**  **0**  **0**  **0**  **0**  **0**  **0** |
| Länsstyrelsen Västmanland (County Administrative Board of Västmanland, Sweden) https://www.lansstyrelsen.se/vastmanland | July 29, 2020 | **Used the regular search box for the entire website**  enskil* AND avlopp*  små* AND avlopp*  va-anläggning*  septiktank* septitank* markbädd* markretention* markrening*  fosfor* | **1**  **0**  **0**  **0**  **0**  **0**  **0**  **0**  **1** |
| Länsstyrelsen Västra Götaland (County Administrative Board of Västra Götaland, Sweden) https://www.lansstyrelsen.se/vastra-gotaland | July 29, 2020 | **Used the regular search box for the entire website**  enskil* AND avlopp*  små* AND avlopp*  va-anläggning*  septiktank* septitank* markbädd* markretention* markrening*  fosfor* | **1**  **2**  **0**  **0**  **0**  **0**  **0**  **0**  **2** |
| Länsstyrelsen Örebro (County Administrative Board of Örebro, Sweden) https://www.lansstyrelsen.se/orebro | July 29, 2020 | **Used the regular search box for the entire website**  enskil* AND avlopp*  små* AND avlopp*  va-anläggning*  septiktank* septitank* markbädd* markretention* markrening*  fosfor* | **1**  **0**  **0**  **0**  **0**  **0**  **0**  **0**  **0** |
| Länsstyrelsen Östergötland (County Administrative Board of Östergötland, Sweden) https://www.lansstyrelsen.se/ostergotland | July 29, 2020 | **Used the regular search box for the entire website**  enskil* AND avlopp*  små* AND avlopp*  va-anläggning*  septiktank* septitank* markbädd* markretention* markrening*  fosfor* | **0**  **0**  **0**  **0**  **0**  **0**  **0**  **0**  **0** |
| Naturvårdsverket (Swedish Environmental Protection Agency) <http://www.naturvardsverket.se> | November 10, 2020 | **Used the regular search box for the entire website Limit: Publications**  enskil* AND avlopp*  små* AND avlopp*  va-anläggning*  septiktank* septitank* markbädd* markretention* markrening*  fosfor* | **11**  **1**  **0**  **0**  **0**  **1**  **0**  **0**  **4** |
| SGU (Geological Survey of Sweden)  https://www.sgu.se | November 11, 2020 | **Used the search box in their publication service GeoLagret https://apps.sgu.se/geolagret/**  "enskilda avlopp"  "enskilda avloppssystem"  "enskilda avloppsanläggningar"  "enskilt avlopp"  "enskilt avloppssystem"  "enskild avloppsanläggning"  "små avlopp"  "små avloppssystem"  "små avloppsanläggningar"  "småskaliga avlopp"  "småskaligt avlopp"  "småskaliga avloppssystem"  "småskaligt avloppssystem"  "småskaliga avloppsanläggningar"  "va-anläggning"  "va-anläggningar"  septiktank* septitank* markbädd* markretention* markrening*  fosfor* | **0**  **0**  **0**  **0**  **0**  **0**  **0**  **0**  **0**  **0**  **0**  **0**  **0**  **0**  **0**  **0**  **0**  **0**  **0**  **0**  **0**  **0** |
| SLU (Swedish University of Agricultural Sciences) <https://www.slu.se> | November 11, 2020 | **Used Advanced search in their publication service Epsilon https://pub.epsilon.slu.se/cgi/search/advanced Search field: Abstract, all of the words**  "on site" wastewater phosphorus  "on site" “waste water” phosphorus  onsite wastewater phosphorus  onsite "waste water" phosphorus  small wastewater phosphorus  small "waste water" phosphorus  "on site" sewage phosphorus  onsite sewage phosphorus  small sewage phosphorus  septic phosphorus  wastewater phosphorus column*  "waste water" phosphorus column*  sewage phosphorus column*  enskil* avlopp* fosf*  små* avlopp* fosf*  septiktank*  septitank*  avlopp* fosf* kolonn*  avlopp* fosf* jordkolonn* | **2**  **0**  **0**  **0**  **0**  **0**  **0**  **0**  **0**  **1**  **0**  **2**  **0**  **6**  **0**  **0**  **0**  **0**  **0** |
| SMED (Swedish Environmental Emissions Data)  https://www.smed.se | November 12, 2020 | **Used the regular search box for the entire website**  **Automatic truncation is used (it's a default functionality)**  enskilda avlopp  enskilt avlopp  enskild avloppsanläggning  små avlopp  småskaliga avlopp  småskaligt avlopp  septi markbädd markretention markrening  fosfor | **13**  **0**  **0**  **2**  **0**  **0**  **0**  **0**  **0**  **0**  **9** |
| Svenskt Vatten (Swedish Water & Wastewater Association)  http://www.svensktvatten.se | November 12, 2020 | **Used the regular search box for the entire website**  "enskilda avlopp"  "enskilda avloppssystem"  "enskilda avloppsanläggningar"  "enskilt avlopp"  "enskilt avloppssystem"  "enskild avloppsanläggning"  "små avlopp"  "små avloppssystem"  "små avloppsanläggningar"  "småskaliga avlopp"  "småskaligt avlopp"  "småskaliga avloppssystem"  "småskaligt avloppssystem"  "småskaliga avloppsanläggningar"  "va-anläggning"  "va-anläggningar"  septiktank  septiktankar septitank  septitankar markbädd  markbäddar markretention markrening  fosfor | **0**  **0**  **0**  **0**  **0**  **0**  **0**  **0**  **0**  **0**  **1**  **0**  **0**  **0**  **2**  **0**  **0**  **0**  **0**  **0**  **3**  **0**  **0**  **0**  **6** |
| Tidskriften Vatten (Water, a Swedish journal)  https://www.tidskriftenvatten.se | November 12, 2020 | **Used Article search**  **https://www.tidskriftenvatten.se/ptb-search/ Search field: Title**  on site wastewater phosphorus on site waste water phosphorus onsite wastewater phosphorus  onsite waste water phosphorus small wastewater phosphorus  small waste water phosphorus on site sewage phosphorus onsite sewage phosphorus small sewage phosphorus septic wastewater phosphorus column  wastewater phosphorus columns waste water phosphorus column  waste water phosphorus columns  sewage phosphorus column  sewage phosphorus columns  enskilda avlopp  enskilda avloppssystem  enskilda avloppsanläggningar  enskilt avlopp  enskilt avloppssystem  enskild avloppsanläggning  små avlopp  små avloppssystem  små avloppsanläggningar  småskaliga avlopp  småskaligt avlopp  småskaliga avloppssystem  småskaligt avloppssystem  småskaliga avloppsanläggningar  va-anläggning  va-anläggningar  septiktank  septiktankar septitank  septitankar markbädd  markbäddar markretention markrening  fosfor | **5**  **0**  **0**  **0**  **3**  **2**  **0**  **0**  **0**  **5**  **1**  **0**  **0**  **0**  **0**  **0**  **0**  **0**  **0**  **0**  **0**  **0**  **4**  **0**  **0**  **0**  **0**  **0**  **0**  **0**  **0**  **0**  **0**  **0**  **0**  **0**  **0**  **0**  **0**  **0**  **7** |
| DEFRA (Department for Environment, Food & Rural Affairs)  https://www.gov.uk/government/organisations/department-for-environment-food-rural-affairs | December 3, 2020 | **Used the regular search box for the entire website**  **Limit to Content type: "Research and statistics"**  wastewater  “waste water”  sewage  septic  phosphorus | **0**  **0**  **3**  **0**  **6** |
| SEPA (Scottish Environmental Protection Agency)  https://www.sepa.org.uk | December 3, 2020 | **Used the search box on the publications page**  **https://www.sepa.org.uk/library/** wastewater sewage septic  phosphorus | **1**  **1**  **1**  **1** |
| INRS (The Institut national de la recherche scientifique, in Canada)  http://www.inrs.ca | February 3, 2021 | **Used Advanced search in their digital repository http://espace.inrs.ca/cgi/search/advanced**  **Search field: Résumé (abstract)**  "on site" wastewater  "on site" “waste water”  onsite wastewater  onsite "waste water"  small wastewater  small "waste water"  "on site" sewage  onsite sewage  small sewage  septic phosphorus  wastewater phosphorus column*  "waste water" phosphorus column*  sewage phosphorus column* | **1**  **0**  **0**  **0**  **1**  **0**  **1**  **0**  **0**  **0**  **0**  **0**  **0** |
| EPA U.S. (Environmental Protection Agency, United States) <https://www.epa.gov/> | February 19, 2021 | **Used Fields Search on National Service Center for Environmental Publications (NSCEP) http://nepis.epa.gov/Fields.html Search Field: Title Results Precision: Exact match**  **Limits: Only PDF-publications**  on site AND wastewater  on site AND waste water  onsite AND wastewater  onsite AND waste water  small AND wastewater  small AND waste water  on site AND sewage  onsite AND sewage  small AND sewage  decentralized AND wastewater  decentralized AND waste water  decentralized AND sewage  septic  phosphorus | **14**  **0**  **12**  **0**  **23**  **0**  **2**  **1**  **1**  **4**  **0**  **0**  **43**  **63** |
| NOWRA (National Onsite Wastewater Recycling Association)  http://www.nowra.org/ | February 22, 2021 | **Browsed through the conference publications** | **4** |
| CSIRO (Commonwealth Scientific and Industrial Research Organisation, in Australia)  https://www.csiro.au | February 3, 2021 | **Used Advanced Search on the NSCEP publication site**  **https://publications.csiro.au/publications/advancedSearch Setting: Search in Title or Abstract**  "on site" AND wastewater AND phosphorus  "on site" AND “waste water” AND phosphorus  onsite AND wastewater AND phosphorus  onsite AND "waste water" AND phosphorus  small AND wastewater AND phosphorus  small AND "waste water" AND phosphorus  "on site" AND sewage AND phosphorus  onsite AND sewage AND phosphorus  small AND sewage AND phosphorus  septic AND phosphorus  wastewater AND phosphorus AND column*  "waste water" AND phosphorus AND column*  sewage AND phosphorus AND column* | **0**  **0**  **0**  **0**  **0**  **0**  **0**  **0**  **0**  **1**  **0**  **0**  **3** |
