# Supplementary material for: Existing evidence related to soil retention of phosphorus from on-site wastewater treatment systems in boreal and temperate climate zones: a systematic map
Source: Environ Evid. 2023 Apr 3;12:6. doi: 10.1186/s13750-023-00300-7 (PMC11378865; doi:10.1186/s13750-023-00300-7)
Supplement: Supplementary file 3 — Additional file 3. Benchmark studies. [file 13750_2023_300_MOESM3_ESM.docx]

*README*

Title: Benchmark studies

Description: This additional file describes a list of published studies that is relevant to the subject matter of the systematic map. These studies are called "benchmark studies" and this list was used during the development of the search strings and to test the comprehensiveness of the search.

**Benchmark studies**

It should be noted that it is not ensured that all of the listed benchmark studies fulfill the criteria and will be included in the systematic map. However, they are all sufficiently relevant to be used to test the comprehensiveness of the search.

**Benchmark field studies**

Batista Seguí MDM, Tyrrel S, Hess T, Sakrabani R, Knaggs D. Long-term phosphorus accumulation and removal efficiency in a land-based wastewater treatment system in the UK. Water and Environment Journal. 2019;33(4):589-98.

Eveborn D, Gustafsson JP, Elmefors E, Yu L, Eriksson AK, Ljung E, et al. Phosphorus in soil treatment systems: Accumulation and mobility. Water Research. 2014;64:42-52.

Eveborn D, Kong D, Gustafsson JP. Wastewater treatment by soil infiltration: Long-term phosphorus removal. Journal of Contaminant Hydrology. 2012;140-141:24-33.

Gill LW, O'Luanaigh N, Johnston PM, Misstear BDR, O'Suilleabhain C. Nutrient loading on subsoils from on-site wastewater effluent, comparing septic tank and secondary treatment systems. Water Research. 2009;43(10):2739-49.

Humphrey C, Serozi B, Jernigan J, Iverson G, Pradhan S, O'Driscoll M, et al. Phosphate treatment by onsite wastewater systems in nutrient-sensitive watersheds of North Carolina's piedmont. Water Science and Technology. 2016;74(7):1527-38.

Jones RA, Lee GF. Septic tank wastewater disposal systems as phosphorus sources for surface waters. Journal of the Water Pollution Control Federation. 1979;51(11):2764-75.

Liu W, Cui LH, Zhou YP, Lei ZX. Removal of total phosphorus from septic tank effluent by the hybrid constructed wetland system. Procedia Environ Sci. 2011;10:2102-7.

Ptacek CJ. Geochemistry of a septic-system plume in a coastal barrier bar, Point Pelee, Ontario, Canada. Journal of Contaminant Hydrology. 1998;33(3-4):293-312.

Robertson WD. Development of steady-state phosphate concentrations in septic system plumes. Journal of Contaminant Hydrology. 1995;19(4):289-305.

Robertson WD. Irreversible phosphorus sorption in septic system plumes? Ground Water. 2008;46(1):51-60.

Robertson WD. Phosphorus retention in a 20-year-old septic system filter bed. Journal of Environmental Quality. 2012;41(5):1437-44.

Robertson WD, Schiff SL, Ptacek CJ. Review of phosphate mobility and persistence in 10 septic system plumes. Ground Water. 1998;36(6):1000-10.

Robertson WD, Van Stempvoort DR, Schiff SL. Review of phosphorus attenuation in groundwater plumes from 24 septic systems. Science of the Total Environment. 2019;692:640-52.

Roy JW, Spoelstra J, Robertson WD, Klemt W, Schiff SL. Contribution of phosphorus to Georgian Bay from groundwater of a coastal beach town with decommissioned septic systems. Journal of Great Lakes Research. 2017;43(6):1016-29.

Sawhney BL, Starr JL. Movement of phosphorus from a septic system drainfield. Journal of the Water Pollution Control Federation. 1977;49(11):2238-42.

Schellenger FL, Hellweger FL. Phosphorus loading from onsite wastewater systems to a lake (at long time scales). Lake and Reservoir Management. 2019;35(1):90-101.

Speed CD, Fretwell BA, Davison PS. The role of septic tanks in the dissolved phosphorus budget of the Upper River Nar and possible implications for other catchments. Quarterly Journal of Engineering Geology and Hydrogeology. 2019;52(1):23-37.

Withers PJA, Jarvie HP, Stoate C. Quantifying the impact of septic tank systems on eutrophication risk in rural headwaters. Environment International. 2011;37(3):644-53.

Zanini L, Robertson WD, Ptacek CJ, Schiff SL, Mayer T. Phosphorus characterization in sediments impacted by septic effluent at four sites in central Canada. Journal of Contaminant Hydrology. 1998;33(3-4):405-29.

**Benchmark laboratory soil-column studies**

Mechtensimer S, Toor GS. Fate, mass balance, and transport of phosphorus in the septic system drainfields. Chemosphere. 2016;159:153-8.

Pell M, Nyberg F. Infiltration of wastewater in a newly started pilot sand-filter system: I. Reduction of organic matter and phosphorus. Journal of Environmental Quality. 1989;18(4):451-7.

Zurawsky MA, Robertson WD, Ptacek CJ, Schiff SL. Geochemical stability of phosphorus solids below septic system infiltration beds. Journal of Contaminant Hydrology. 2004;73(1-4):129-43.
